# International transfer and translation of an end of life care intervention: the case of the Liverpool Care Pathway for the dying patient

**DOI:** 10.12688/wellcomeopenres.16321.1

**Published:** 2020-10-27

**Authors:** David Clark, Hamilton Inbadas, Jane Seymour

**Affiliations:** 1 School of Interdisciplinary Studies, University of Glasgow, Dumfries, DG2 0XY, UK; 2 Health Sciences School, University of Sheffield, Sheffield, South Yorkshire, S10 2HQ, UK

**Keywords:** palliative care, end of life care, Liverpool Care Pathway, policy transfer and translation, boundary object

## Abstract

We explore how and why the Liverpool Care Pathway (LCP) for the dying patient was transferred to 20 countries beyond the UK, and with what consequences for policy and practice. Our paper synthesises findings from 95 publications contained in a historical narrative literature review on the implementation of the LCP outside the United Kingdom, alongside data from 18 qualitative interviews with 19 key actors involved with the LCP in 14 countries. We use the review to explore the timelines and patterns of development and implementation in the specific countries, to consider what forms of research and evaluation about the LCP were undertaken to establish its effectiveness, and to summarise the resulting findings and their consequences. We use the interviews to gain insights into the elements, processes and dynamics that shaped the transfer and translation of the LCP from one location to another, across national boundaries. Using six questions from the policy transfer literature we then explain who were the key actors involved; what was transferred; from where lessons were drawn; the different degrees of transfer that took place; what restricted or facilitated transfer; and how transfer was related to ‘success’ or ‘failure’. We conclude that the spread of the LCP took place mostly in prosperous countries, and was sustained over around 15 years.  It took in differing geographies and cultures, and a variety of linguistic, policy and practice contexts. If it did not succeed in a wider transformational goal, it appears to have been well received and perceived as beneficial in many contexts, largely avoiding accusations of mis-use and harm that had occurred in the UK, and in some cases fostering a sustained international collaboration and ongoing use of local variants, even after withdrawal in its country of origin in 2014.

          “… human characters emplotted in a story of (in the early stages) pioneering endeavour and (later) systematic puzzle-solving, variously embellished with scientific dramas, surprises and ‘twists in the plot’”

          (Greenhalgh *et al.* 2005 ^1^)

                                                                      *****

          “We’ve worked with the LCP programme not just in England, but in over 20 countries in the world, we have translations in six languages and it’s recognised around that best care for the dying patient”

          Q. Have they done it better in other countries? Have they been tougher about training and monitoring?

          “I think they are more systematic in implementation systems in other countries.”

          Q. That’s a yes …

          (Professor John Ellershaw interviewed on BBC Radio 4 Report programme: The Liverpool Care Pathway, 15 August 2013 ^2^)

## Introduction

It is estimated that 80% of those who die could benefit from palliative care to relieve physical, mental, spiritual and social problems at the end of life. Yet despite demonstrable need, there have been difficulties in identifying appropriate, evidence-based interventions that are scalable across jurisdictions and settings and which might ameliorate suffering when a person is dying. Developed by Professor John Ellershaw and colleagues, the Liverpool Care Pathway (LCP) showed great potential as such an intervention when first formulated in the United Kingdom (UK) in the late 1990s, and where initially it was widely endorsed by policy makers and became a key element in government strategy for end of life care. During the following decade and beyond, this led to further interest in and implementation of the LCP internationally. Over the course of its development, the LCP went through 12 versions in total. In the light of growing professional and public concern about its widespread use in the UK however, including claims that it denied access to treatment or even hastened death, a government appointed review, led by Rabbi Julia Neuberger, recommended that it should be discontinued, with effect from 2014.

The circumstances surrounding the discontinuation of the LCP make up perhaps the most significant *cause celèbre* in the field of modern palliative care. At the time of its withdrawal and subsequently, most commentators within the UK addressed themselves to the local situation ^3– 7^. Conversely, some commentaries were focussed on the UK context, but were generated elsewhere ^8, 9^. In addition, a trickle of UK research studies focussing on the workings of the LCP continued to appear, from the point of its abandonment and after it had ceased to be used ^10, 11^. 

In this extended paper, we explore one dimension of the LCP story that has attracted no attention from commentators or researchers, but is of considerable significance to policy and practice. We refer to the international spread of the LCP in the years before its withdrawal in the UK and also its continuing saliency in countries other than the UK since that time. This issue is not only a vital component in the ongoing international growth of palliative care, but illustrative of how and with what consequences a specific end of life care intervention can be transferred and translated into a variety of contexts, some of them strikingly dissimilar to its place of origin and initial implementation. It therefore adds to our growing understanding of the reverberations surrounding the LCP and also offers wider lessons about policy transfer in end of life care.

The initial spread of LCP beyond the UK, as we shall see, was mainly dependent on personal contacts and contingent circumstances. Building on this, a European funded initiative, known as OPCARE9 ^12^. and led by John Ellershaw ran from March 2008 to March 2011 ^13^. It aimed to ‘optimise research and clinical care for cancer patients in the last days of life’ ^14^. Then, bolstered by the perceived benefits of LCP implementation outside of the UK and the success of the OPCARE9 Collaborative, an LCP International Reference Group (IRG) was launched in March 2011 to support the further development of the pathway internationally. It consolidated a process that had begun over a decade earlier, but it also came in the face of mounting concern about the LCP and the imminent announcement of the Neuberger review. To that end the IRG, meeting in November 2012 to review the implications of the growing debate around the pathway, produced a consensus statement and identified a way forward. It set out a belief in the need to improve the care of the dying, along with three specific goals (best possible care, effective communication, robust education and training) and called for more organisational oversight and accreditation of end of life care. But it made no reference to the LCP.

The IRG had members from 13 countries. Despite mounting problems in the UK, confidence in the LCP approach was underpinned by the experience of IRG members, all of whom had been involved in its implementation in their own country, and through which ‘the LCP has been shown to be transferable for use in other languages and very different cultural contexts’ ^14^. Ellershaw and his colleagues further observed that: ‘… as the debate continues in England, the LCP’s country of origin, could an international perspective provide the next steps in improving care of the dying?’ Here making no reference to the Neuberger report, they took a positive view of the ‘recent media’ reporting, concluding that it may yet prove a helpful contributing factor, if it were to help ‘drive up’ research and clinical excellence for the care of the dying. This is a well-known policy trope that suggests positive things can emerge from ‘scandals’ ^15^.

In November 2013 the group met again and set out its response to the Neuberger review ^16^. It also agreed to establish the International Collaborative for Best Care for the Dying Person ^17^, and issued ‘The Liverpool Declaration’ with a vision ‘for a world where all people experience a good death as an integral part of their individual life, supported by the very best personalised care’. The Marie Curie Palliative Care Institute Liverpool would be the co-ordinating centre for the Collaborative ^18^. In this manner the impetus that had been generated by the LCP internationally was maintained, albeit by a small number of protagonists, and with moderate published output.

Building on our earlier research scrutiny of the rise and demise of the LCP in the UK ^19^, we seek here to stimulate critical reflection on the risks and benefits of disseminating an end of life care intervention across international boundaries, in contexts where there are uncertainties about the original evidence base, and where key actors may not be in a position to anticipate the wider ramifications of what they do.

## Theoretical frameworks

To make sense of this issue, we adopt ideas from the concept of ‘policy transfer’, to shape our research questions, help interpret our findings and also to inform the wider discussion that results. The significance of policy transfer in this context was recognised by Professor Sir Howard Newby, then Vice-Chancellor of Liverpool University in introducing the work of OPCARE9. The work, he stated, was ‘about knowledge transfer - not just from the laboratory to the bedside but from one country to another. It is vital that we continue to share our experience and expertise among European colleagues and further afield to help improve care of the dying globally’ ^20^.

Policy transfer, when first fully articulated as a concept in the 1990s, was largely restricted to political jurisdictions and state actions across boundaries ^21^. Over time it broadened to include ‘voluntary’ as well as more ‘coercive’ forms of transfer and in particular to capture the involvement of a wider range of participants and settings, including non-state actors, pressure groups, supra-national agencies and advocacy organisations. This wider focus is well described by Benson and Jordan ^22^ in 2011, who also discuss the motivations of those involved in policy transfer, including a sense of frustration about policy development in specific areas, and attempts to rectify this through persuasion and voluntary transfer from one setting to another. Likewise, they show how the emphasis has shifted from ‘hard’ forms of transfer involving institutions, policy goals and measureable outcomes, towards ‘softer’ forms that involve ideas, concepts, and where lines of transfer are horizontal rather than vertical.

Ideas about the ‘transfer’ of policies, processes, systems and actions are closely linked to the question of translation. Challenges in the ‘transfer’ and ‘translation’ of palliative care models from one setting to another have been highlighted by Zaman *et al.* ^23^ who ask: ‘Should we focus on the transfer of palliative care narratives, assumptions, policies and practices from developed to developing countries, or should our emphasis be on the translation of these things in both directions?’ (p76). An exploration of policy translation by Freeman ^24^, is helpful in this regard. He notes that commentators often use the phrase ‘lost in translation’ to indicate that things have gone wrong, but he also suggests that translation may be the ‘lubricant’ or ‘key’ to transfer.

When multiple social actors are engaged in the processes of translation, we have found it useful to draw on the idea of the ‘boundary object’ and have ourselves argued that the LCP can be seen in this light. A boundary object needs to be malleable enough to work in specific local contexts but rigid enough to maintain its integrity across settings. As the originators of the concept of ‘boundary object’ note, ‘protocols are not simply the imposition of one world's vision on the rest; if they are, they are sure to fail. Rather, boundary objects act as anchors or bridges' (Star and Griesemer 1989 ^25^, quoted in Freeman 2009).

We consider that translation of policy thus conceived, becomes a complex, dynamic, multi-lateral affair, full of pitfalls, potentialities, opportunities, road maps and *culs de sac*. Taking an idea or practice from one place to another will therefore depend on far more than its integrity or robustness. Such transfer will require negotiation, interpolation, bargaining and flexibility in the rules of encounter. Freeman captures precisely the ground we are interested in here, and which links in turn to our earlier paper on the LCP in the UK:

Translation is something like a boundary object. It is not an object, of course, but a practice and vocabulary within which the nature of research, policy and practice and the relationship between them is being rethought. It is the means by which an array of actors, including international organizations both public and private, governments, sponsors, researchers, policy makers and practitioners have come to communicate about a problem even in the absence of any fully shared conception of it. These debates about translation are themselves instances of it (p445).

Two major protagonists in the field, Dolowitz and Marsh (the former interestingly a member of staff at Liverpool University, from which John Ellershaw led his programme of work on the LCP), in a classic paper of 2000, set out their conceptual framework for the analysis of policy transfer ^26^. The framework is organised around six questions: 1) who are the key actors involved? 2) what is transferred? 3) from where are lessons drawn? 4) what are the different degrees of transfer? 5) what restricts or facilitates transfer? 6) how is transfer related to ‘success’ or ‘failure’? Towards the end of this paper, we map these questions on to our data about the international spread of the LCP.

Circling around this central theme of policy transfer, the analysis framework and the role of the boundary object within it, we therefore want to find ways to recognise that making sense of the international spread of the LCP also involves a deeper understanding of knowledge construction and interpretation across differing jurisdictional and cultural settings ^27^. Our two main sources for this (published literature and interviews with key actors) point to the relationship between formal knowledge claims and their consequences and implications at a local level, and are bolstered by the kind of theoretical frameworks described here. They help us to make sense of a rich and extensive experiment in the transfer of ideas and practices designed to work in many locations with the goal to improve end of life care, even in the absence of any fully shared conception of what that is, to paraphrase Freeman.

## Aim and research questions

Our aim in this paper is to construct a detailed case study of how the LCP, an end of life care clinical intervention that had been developed in the UK, was adopted in 20 other countries and the consequences that resulted from this, including when the intervention was withdrawn from use in its country of origin. We have taken as our research questions, those posed by Dolowitz and Marsh and we weave these in with a number of LCP-specific questions and themes that emerged from our analysis.

## Methods

We made use of two principal approaches 1) a historical narrative review of published and grey literature relating to the LCP in the international context and 2) qualitative interviews with key actors involved in LCP implementation, research or discussion in countries outside the UK.

### Ethical statement

The study was approved by the Research Ethics Committee of the University of Glasgow, College of Social Sciences, on 2 April 2017, project number 400160110. Prior to data collection, the purpose of the study was explained to all participants and written consent for ‘on the record’ participation in the study was obtained from each participant at the time of interview. Participants were informed that they had the right to withdraw from the study at any point in time, without any repercussions.

### Historical narrative review

The historical narrative review of the literature and associated commentaries (2000–19) on the use of the LCP outside of the UK is presented as part of the *Underlying data ^28^* to this paper and is intended to serve as a resource for further study. Our inclusion criteria for the review were: studies (and wider commentaries) on the use of the LCP in countries outside of the UK, including published articles, conference abstracts and presentations, reports and grey literature. Our exclusion criteria were: studies (and wider commentaries) on the use of the LCP within the UK, including published articles, conference abstracts and presentations, reports and grey literature; we also excluded all studies referring to ‘pathways’ from the UK and elsewhere, which did not refer explicitly to LCP.

A baseline English language PubMed ‘all text’ search for ‘Liverpool Care Pathway’ was conducted in May 2019. It generated 39/211 outputs, which met the inclusion criteria. Further searches were conducted in January 2020, with the following results: CINHAL 5/22; PsychInfo 3/53; SCOPUS 2/252; Proquest 0/47. The searches identified 49 outputs in total. Hand searching of this material for further relevant references, along with Google searches relating to the use of the LCP outside the UK and personal communication with other researchers, plus examples given to us from those we interviewed, together yielded a further 46 outputs. This process involved judicious use of Google translate as well as assistance from colleagues with particular linguistic skills (Mandarin, Japanese, Norwegian, German, Spanish). Some non-English outputs contained abstracts and summaries in English.

The total number of outputs contained in this review is therefore 95, covering 20 jurisdictions ^29^. We realise of course that this will not be comprehensive and we welcome suggestions for further outputs that might be included in revised versions of the review. We are much in agreement with Greenhalgh and Peacock when they state:

Systematic review of complex evidence cannot rely solely on predefined, protocol driven search strategies, no matter how many databases are searched. Strategies that might seem less efficient (such as browsing library shelves, asking colleagues, pursuing references that look interesting, and simply being alert to serendipitous discovery) may have a better yield per hour spent and are likely to identify important sources that would otherwise be missed ^30^.

### Qualitative interviews

***Sampling.*** Purposive and snowballing sampling techniques were used to identify potential participants for qualitative semi-structured interviews about the international spread of the LCP. This approach to sampling facilitates the choice of respondents who are strategically located in a situation from where they are able to shed light on the subject of study at hand ^31, 32^. The target group included: clinicians in leading roles with experience of LCP implementation, researchers who had studied the LCP outside of the UK, policy makers involved in LCP introduction, and global experts in palliative care with knowledge of LCP introduction in particular non-UK settings.

The initial sampling frame consisted of those individuals reporting on the use of the LCP in the 2011 LCP handbook ^33^, totalling 11 countries (Argentina; Slovenia; India; Norway; Italy; Switzerland, Germany and Austria [the DACH German speaking collaborative]; Sweden; Netherlands; New Zealand). Everyone we approached agreed to take part, with the exception of one person (Slovenia). Following leads from the linked literature review and recommendations from interviewees, we then invited potential interviewees from seven further countries where there was evidence of LCP implementation; people from four countries accepted (Australia, Belgium, Denmark and Japan), whilst three (from Hong Kong, Ireland and Spain) declined to take part. In two instances (New Zealand, Belgium) two people took part in the same interview. For some countries we had interviews with more than one person: in two countries we interviewed two separate individuals (Australia, Japan), and in one country we interviewed the same person twice (Netherlands). We thereby completed 19 interviews with 20 people from 14 countries in total – though these figures are each reduced by one, following the withdrawal of one interviewee from the study. The analysis here is therefore based on 18 interviews with 19 people. The interviews took place between August 2017 and December 2019.

Unfortunately, we had to proceed in this work without the involvement of Professor John Ellershaw, leader of the LCP international initiative. We had hoped he would be our first interviewee, in the manner of an ‘index case’, setting out his perspective on the international spread of the LCP and guiding us towards others who could assist with our research, but he declined to participate in our study ^34^.

***Recruitment.*** We sent introductory emails to potential interviewees, explaining the purpose of the study and enclosing an information sheet. Individuals were invited to a telephone or SKYPE interview at a mutually convenient time. We asked individuals to consider participating in an ‘on the record’ interview (although this was not mandatory), since interviewees were likely to be easily identified by colleagues in the palliative care field from our resulting reports and publications. Individuals who agreed to take part in an interview were asked to complete a consent form, and to indicate on the latter whether they were willing to participate ‘on the record’. All of them agreed to this.

**Figure 1.**
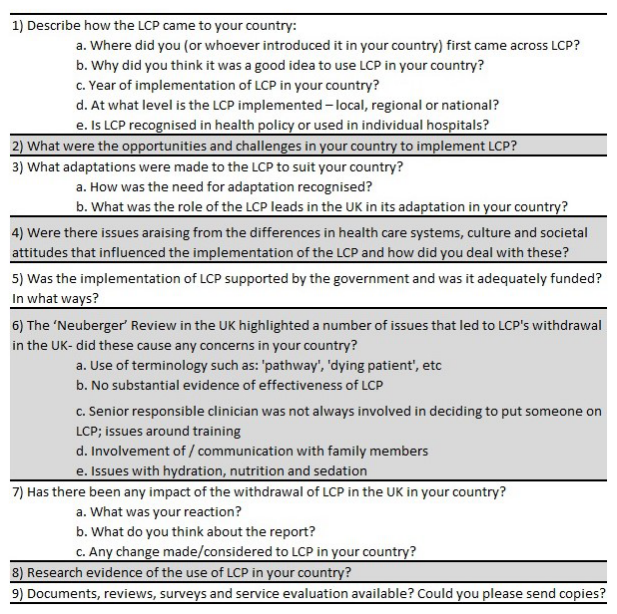
Interview *aide memoire*.

***Conduct and analysis of the interviews.*** We developed an *aide memoire* (see Figure 1) based on the aims of our project and themes in the literature review. Interviews were audio recorded and transcribed by a specialist agency, bound by a confidentiality clause. The *aide memoire* was adapted according to the context of the interview and the background of the interviewee. Where appropriate, we sometimes included more than one person in the interview. In one case (the Netherlands), we carried out a repeat interview to clarify material and to bring our understanding of research developments up to date. In another case (Belgium), the interview was conducted in two parts because of technical challenges and poor sound quality in the first part of the interview. Interviews ranged in length from 36 to 66 minutes. All three authors were involved with the interviews (Inbadas, 12; Seymour, 5; Clark, 2).

Our analysis followed the principles of the framework approach to qualitative data, as described by Gale and colleagues ^32^. Framework analysis sits within the broad range of thematic approaches to qualitative analysis and is especially appropriate where interviews have followed a similar structure or line of discussion. Its key feature involves the development of matrices to enable systematic comparison of content between ‘cases’ or interviews (sometimes called ‘charting’). It can complement the use of other more analytical strategies: for example, in this study we also undertook a preliminary coding exercise using NVIVO 11 and, as a step towards interpretation, subsequently developed a detailed narrative ‘write up’ for each interview. 

## Results

### International literature review

Two key dimensions were identified in the review.

**Figure 2.**
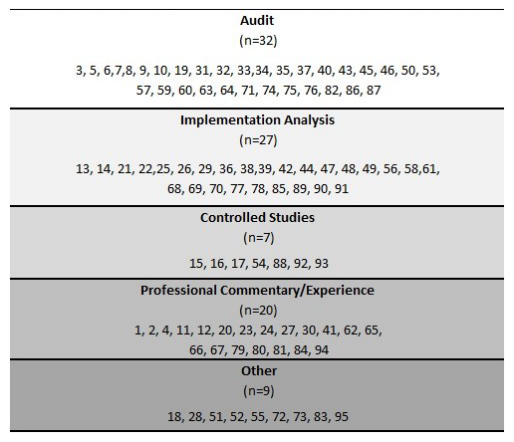
Studies and publications by type and referenced to the literature review (n=95).

First is a story about the international spread and publication activity associated with an end of life care intervention. Its parameters are the countries and timelines and intensity of production ( Table 1). Second is a story about the evidence generated to shed light on the use of the intervention in jurisdictions outside the UK. This relates to the frames of evaluation that were adopted to study the LCP, the principles and designs that were used, the settings in which they were deployed, the results that emerged from these endeavours and the forms of adaptation made to the LCP, contingent upon its use in settings outside the UK ( Figure 2). 

**Table 1.** LCP publication by country and year.

	2003	2004	2005	2006	2007	2008	2009	2010	2011	2012	2013	2014	2015	2016	2017	2018	2019	No date	Total by country
Argentina								1			1								2
Australia				1			1	1	1	1		1	1						7
Austria							1		1	1	1								4
Belgium													2	1	1				4
China									1										1
Denmark														1					1
Germany							3				1		1		1				6
Hong Kong						1		1											2
India								1	1										2
Ireland								1											1
Italy									4	1	1	5	1						12
Japan						1			8	1		2	2	2	1				17
Netherlands	1	1		3		2	1	1		1	1		1			1			13
New Zealand					1	1			3	3									8
Norway													1	1	1				3
Singapore										1			1						2
Slovenia																			0
Spain										1								1	2
Sweden												1		2		2		1	6
Switzerland					1					1									2
Total by year	1	1	0	4	2	5	6	6	19	11	5	9	10	7	4	3	0	2	95

***International spread and publication activity***

Publication patterns reveal international and local diffusion

The literature search produced outputs from 19 countries, to which the LCP had been transferred in some way. To this can be added a commentary on Slovenia, from the LCP handbook of 2011 ^33^. If we add the UK to the total, this brings us to 21, or the figure of ‘more than 20 countries’ that is frequently cited in LCP literature.

The diffusion from the UK to other countries was most evident in parts of western Europe; it was significant in parts of East Asia and Australasia; confined to one country per region in Eastern Europe, the Indian subcontinent, and South America; and non-existent in Africa the Middle East, the USA, and Canada. LCP was therefore a phenomenon essentially of the Global North, albeit without North America (Table 1). Only two countries to which is was transferred were in the low and middle income category (India and Argentina).

The LCP first appeared beyond British shores in 2001 in a Dutch translation based on LCP Version 4 that was piloted in three palliative care settings (hospital, nursing home and hospice). In total, 13 publications about the LCP appeared from Dutch researchers between 2003 and 2018, gradually spreading beyond the original three settings to cover the whole of the country. By 2012 the 'Zorgpad Stervensfase', as the Dutch version was known, had been implemented in over 100 healthcare institutions in the Netherlands

Belgium, by contrast, whilst working with Dutch and Italian researchers, produced no studies on LCP as such, but conducted work leading to four publications (2015–17) on an intervention that had grown out of the LCP, and in particular was developed in light of the critique of LCP that had arisen in the UK. Implemented in 10 hospitals in the Flanders region, it was focussed on the end of life care of older patients in acute geriatric settings.

Just one relevant study was identified from Ireland. This may seem surprising given the proximity to LCP origins in Liverpool, and a shared language. It does appear however that the Hospice Friendly Hospitals programme ^35^, which got underway in 2007 and adopted an all-systems approach to improving end of life care in acute and community hospital settings in Ireland, saw LCP and other related pathways as useful resources for hospital end of life care improvement, but was reluctant to advocate for the LCP specifically.

German language clinicians and researchers formed a three-country group (Germany, Austria and Switzerland) to promote their shared interests in LCP and this resulted in 13 publications between 2007–2017, including one journalistic commentary. Although LCP gained traction in Switzerland and had an influence on federal planning, coverage in the other two countries was much more limited.

Among the Nordic countries, Norway was a significant early adopter in 2005–6 (especially in nursing homes) but produced almost no primary published work on LCP, though it did generate wider commentary and review. The 2011 chapter in the LCP handbook refers to a ‘flying start to implementation’ in Norway, where the Regional Centre of Excellence for Palliative Care in Western Norway took a co-ordinating and facilitating role. Sweden was more active in pursuing research work on LCP adoption and also established a national centre for co-ordination and knowledge exchange based at the research and development unit of Stiftelsen Stockholms Sjukhem. By 2014 over 200 services in Sweden were using the LCP, including specialist palliative care units, home care, hospital wards and nursing homes and that experience was feeding into the development of a national plan for the care of the dying. In Denmark, implementation was localised to one setting and dissemination restricted to conference presentation. Ten outputs emerged from these three Nordic countries, including one of the few controlled studies, which was conducted in Sweden.

Colleagues in Spain and Argentina collaborated with each other on their interest in implementing the LCP into local services, resulting in four outputs, with a fifth that came to our attention after the analysis was compete, consisting in total of three journal articles and two conference abstracts. Again, the use of LCP was localised in a small number of areas.

Interest in LCP in Australia and New Zealand was evident from 2007 onwards and led to 15 published outputs. In Australia, the work was concentrated in acute hospitals at first and later shifted to include aged care facilities also. In New Zealand, Dedicated Ministry of Health funding for LCP implementation was made available to all 20 Health Boards from 2008, prior to which some Boards had already shown interest in LCP. By these means LCP was adopted to some degree across New Zealand, in four settings – hospices, hospitals, aged residential care facilities and patients’ own homes.

Interest in LCP first appeared in Hong Kong, China, Singapore and Japan from around 2007–8, when the first of 21 publications began to appear, the large majority from Japan, where various studies were conducted in hospital and home settings. In 2010, a special issue of the Japanese Journal of Clinical Nursing was published that focussed entirely on the use of LCP in Japan. Again, specific hospitals were involved in LCP development work, but in the absence of a co-ordinating centre. The LCP initiative in India was endorsed by the national association for palliative care, and a local version was agreed with Liverpool and known as LCP-IICP (Indian Integrated Care Pathway). Enthusiastically supported by collaborators in and outside the country, the initiative was nevertheless small scale, confined to four specific locations (Kerala, in two centres; Chandigarh; and Karnataka) and published only through conference abstracts.

The work on LCP in Italy is not only substantial in terms of published outputs, but, as we shall see in the next section, is arguably the most robust in terms of scientific rigour, and includes the only example of a randomised controlled trial (RCT) of the LCP itself. It is therefore very closely tied to the overall LCP narrative, since the results of the RCT appeared after, and not before, a decision was taken to withdraw the LCP in the UK. Twelve papers on LCP by Italian authors were published between 2011–2015, the penultimate one a review of pathways and evidence. The work was mainly conducted by a team of researchers and clinicians associated with a regional palliative care network of the National Cancer Research Institute and located in Genoa, Italy. It was led by Dr Massimo Costantini in a three-year programme of research funded by the Italian Minister of Health from 2006, to determine whether the implementation of LCP in a hospital setting could be effective in improving end of life care for cancer patients.

Translation, cultures and systems

The LCP international strategy adopted a well-tested and recognised linguistic translation model, based on the principles used by the European Organization for Research and Treatment in Cancer (EORTC). These include forward translations into the target language, reconciliation, back translations into English, proofreading by an independent linguist, pilot-testing and finalisation of the translation ^36^. Several of the reviewed publications make reference to this and to how it was applied to a specific numbered version of the LCP. Occasionally there is reference to some point of linguistic detail where a word such as ‘secretion’ (in the Netherlands) is a verb but cannot be a noun, or in Spain and especially Argentina, there is reference to a cultural translation, which in some unspecified way adapts the LCP to the local culture. In New Zealand and Australia there is also reference to the translation of LCP into the local health care system. Nowhere do these accounts delve more deeply. ‘Translation’ is therefore essentially seen as a technical process, with occasional cultural dimensions, which can be accomplished by careful use of process.

The LCP handbook is not specific about the health care systems into which transfer would be most effective or needed. In general, it takes a somewhat local perspective, focussing on the specific ‘institution’, ‘organization’ or ‘local health economy’ to which LCP can be applied. Yet this can be contrasted with an approach which encouraged the creation of a national or jurisdictional ‘central office’ to co-ordinate and direct LCP implementation and liaise with the ‘Liverpool Central’.

***Frames of evaluation.*** Some form of implementation research, however basic, was conducted in almost all 20 countries that took up the LCP. But in several jurisdictions, implementation research was un-sustained and lacking in strategy; the exceptions to this were Belgium, Italy, and the Netherlands, and to a lesser extent Australia, Japan, Sweden, and New Zealand.

It was possible to assign all 95 outputs in the literature review to a particular category of research or commentary These are shown in Figure 2, in which the numbers given for the publications in each category are the original numbers used in the full literature review that appears in the *Underlying data ^28^* to this paper. In this section we discuss three categories of evaluation that we identified: 1) audit, 2) implementation analysis, 3) controlled studies, along with 4) related commentaries and other works. It should be noted that the following section includes direct mention of 62 references from the literature review, compared to the total figure of 95.

Audit

Most common (n=32) were studies using an audit design and assessing aspects of LCP usage, sometimes with and sometimes without baseline, pre-implementation assessment. Of the 15 countries that undertook these types of study, the most active were the Netherlands (n=7), Japan (n=4). Australia (n=3), and New Zealand (n=3). Some of these audits quickly raised questions about adaptation to local circumstances.

The baseline work was usually undertaken as part of a requirement on the part of ‘LCP Central’, in Liverpool, which favoured pre-implementation case audits of around 20 deceased patients as part of the process of adopting the LCP. For example, the earliest published research on the use of LCP in Australia took place in a network of four hospitals, three hospices and one nursing home in the state of Queensland ^i^. No dates are reported for the period of data collection, but case notes of 20 consecutive patients who had died in each of the institutions were reviewed against the 18 goals that made up the LCP gold standard of care. Each audit was carried out according to LCP protocols, using its standard baseline pro-forma and the work was registered with and supported by the LCP project team in Liverpool. The authors noted that the British-designed audit had not been altered in any way to fit with the Australian context and indicated that aspects of it might not translate to other places, but concluded that, with suitable local modification, care pathways for the dying represented a way forward to improved care and they proposed a network approach to implementation across institutions.

A similar message about local modification came from Hong Kong. Lo and colleagues ^ii^ provided an early commentary on the use of an end of life care pathway in a Chinese population and describe how a group was established in the Tuen Mun Hospital in Hong Kong, to review the work of the LCP and develop a new pathway, modified according to the local situation. Accordingly, the number of goals on the new pathway were reduced from 18 to seven. For example, communication with the general practitioner was removed as most patients in the local context do not have a regular primary care doctor. Likewise, informing relatives of the impending death was not considered necessary, on the grounds that in the Hong Kong context relatives must be told immediately that a person has died, in order to facilitate after-death rituals. In addition, due to workforce pressures, the review periods on the revised pathway were eight hourly, against four hourly in the LCP. The authors describe how this modified pathway was introduced between November 2007 and August 2008 into a designated palliative care ward of the hospital. An audit of the patient records for the period, drew on ‘success criteria’ identified in a Dutch study by Veerbeek *et al.* ^iii^ Using the results of the Veerbeek study, the Hong Kong audit made direct comparisons with data from the Erasmus Medical Centre in Rotterdam (October 2001- January 2003) and the Marie Curie Hospice in Liverpool (April 2002 – July 2003). Patients were on the pathway for a similar average time in all three settings (24–29 hours). The proportion of patients in the palliative care unit in Hong Kong that was enrolled on the pathway was low at 10% in the pilot, reflecting clinicians’ uncertainties in the diagnosis of dying. But one year on this had risen to 40%. The authors concluded that good end of life care could be delivered to Chinese patients using a pathway approach that had been modified from the original LCP.

There was a marked tendency in some of the early audit studies to ascribe considerable benefit to what might seem like very small improvements in pre- and post- results or from very small samples. An example of the former occurred in the Netherlands, where one study compared the level of documentation, symptom burden and aspects of communication before and after the introduction of LCP in 220 patients ^iv^. It found ‘modest but evident’ improvement in the amount of documentation of the patient’s dying phase post-implementation, a ‘small but significant’ reduction in symptom burden, but no difference in relatives’ reported views about communication. Nevertheless, the authors considered this to be ‘a remarkable result of using a care pathway that mainly introduces a structured registration method, rather than a new intervention or therapy’. Similarly, in Switzerland in 2005, although a hospital pilot revealed the need for considerable support in completing the LCP documentation, the authors reported tangible benefits across the pilot stations: faster switching to comfort therapy; greater recognition of the dying process with a more shared language between staff; fewer oversights, due to a more structured procedure; patients and their relatives receiving more comprehensive care; and despite doctors’ scepticism about the value of time spent in the associated round table discussion meetings, the meetings themselves were said to be calmer ^v^. Some substantial claims for improvement were therefore built on a modest platform - in this case of 16 LCP examples.

Audit work of this kind continued until late into the LCP cycle. For example, after the Neuberger report recommendations, an audit of the Care of the Dying Clinical Co-ordinated Pathway (CDP), the local variant of LCP which had been developed with funding from the Health Quality Improvement Fund of the Singapore Ministry of Health, was carried out in the Singapore General Hospital to determine if the use of such a pathway should be continued ^vi^. The audit was conducted in early 2014 and included 740 patients who died on the oncology and renal wards of the hospital from July 2011 to June 2013. A total of 90 oncology patients had been placed on the CDP (12%), compared to 129 renal patients (22%). Most died on the CDP. The authors found no documented compromise in medication safety, clinical monitoring and provision of nutrition and hydration of those placed on the CDP. But documentation of important end of life decisions and conversations was poor, and the proportion of patients placed on the pathway was considered low in relation to figures from the UK. The paper was silent on the direct question of whether the CDP should be withdrawn from use in the hospital, but concluded that an alternative tool, encouraging systematic discussion and documentation of individualized end of life care plans should be considered.

In addition to palliative care units, some general wards in hospitals in Japan also adopted the LCP for their terminally ill patients. A study by Nobuhisa Nakajima, a doctor in a palliative care unit in Sapporo Minamiseisyu Hospital, unlike other empirical studies described here, used a direct Japanese translation of the LCP to provide care for dying patients on a general ward ^vii^. The care pathway was introduced in two phases. Positive outcomes were gained to some extent in the first phase, although the variance rates were relatively high; this was attributed to practitioners’ limited knowledge of symptoms at the end of life and the lack of communication with patients’ families. To improve the practice, the team integrated the Support Team Assessment Schedule (STAS) to enhance knowledge exchange and communication between different parties involved in the care ^37^. As such, the results from the second phase were significantly better.

Although audit studies of this type were often of simple design, in some cases where there was significant attention to process issues, it was difficult for us to separate them categorically from studies that had a stronger emphasis on aspects of implementation. We categorised studies as ‘audit’ when pre and/or post implementation measurement was the main objective; but some studies added other elements to this, such as focus groups with staff or surveys of opinion among professionals. 

Implementation analysis

Where this tendency to mixed methods was stronger and focussed directly on the acceptability as well as the effectiveness of LCP or a local variant, then we classified the study as a form of process or implementation analysis. These studies (n=27) were almost equal in number to the more narrowly executed audit work. The most active of the 10 countries where they took place were New Zealand (n = 5) Japan (n = 5) Germany (n= 4), Italy (n = 4), Australia (n = 3).

A very small minority of studies (the best examples were in Italy and Belgium) drew on the foundations of implementation science, specifically, the principles of complex intervention evaluation. Beyond these, most studies in this group were descriptive in character and concentrated, for example, on issues of LCP acceptability to staff or used the views of lay carers to assess the benefits of the intervention. From the year 2000, the LCP Central Team in Liverpool worked with colleagues from several countries to implement the use of the LCP and focussed on four phases of activity: 1) Induction, 2) Implementation, 3) Dissemination, and 4) Sustainability. In each case there were clear requirements and prescriptions for how the work should proceed. Implementation into pilot sites should ideally follow an algorithm of ‘plan’, ‘do’, ‘study’, ‘act’ – in order to foster continuous learning and some measure of whether improvement occurred. In total, 80% of local staff should take part in an education programme about LCP before first introduction. Periodic status reports should be supplied to LCP Central. The ‘study’ component in the algorithm sometimes led to aspects of research that went beyond audit and captured the views of actors involved in the process, the dynamics and day to day realities of using the LCP, as well as the direct measurement of clinical data.

In a paper on the Dutch pilot process ^viii^ adaptation and translation processes are well described, based on Version 6 of the LCP and adopting principles established by the European Organisation for Research and Treatment on Cancer (EORTC). Within these principles, the ‘forward translation’ (English to Dutch) was undertaken independently by two native Dutch speakers (a doctor and a nurse) and then a third person (a doctor) compared and reconciled the two versions. All three translators worked in palliative care. This version was then subjected to a process of ‘back translation’ when two native English speakers (a palliative care nurse and a professional translator) independently translated the provisional Dutch version back into English. These versions were in turn reconciled by a third person who had been involved with the original development of the LCP, and who verified that the goals of care had not been changed in the translation process. The Dutch version that resulted was given the name ‘Zorgpad voor de Stervenfase-RotterdamZS-r(lcp)’, with the acronym added as a confirmation of its authenticity.

A similar level of rigour accompanied the pilot implementation in a university hospital, where there were interviews with staff to evaluate their perceptions of the pathway. Regular meetings were held to review how the document was working and staff were surveyed on their views, one year after the pilot was completed. If the linguistic translation had been relatively straightforward, the transition into practice was not. There were issues around the meaning of ‘spiritual’ (now replaced by the phrase ‘important values’). ‘Secretion’ was understood in Dutch only as a verb and not as a noun. Adjustments were also required to align the document with procedural practices specific to the Dutch healthcare context, for example concerning information-giving after death.

The follow up hospital questionnaire was administered to a total of 20 nurses and 15 doctors. In total, 22 people responded (63%). The authors reported the item responses in percentage terms and found 72% considered the LCP helpful in structuring patient care and 55% felt the same was true for family and proxy carers. A large majority considered the LCP was helpful in anticipating problems (82%), facilitating multi-disciplinary communication (73%) and contributing to better care in the last days and hours of life. The three Dutch and two English authors could end on an optimistic note: ‘In this way, the potential for promoting optimal care of the dying and comparing outcomes across geographical borders is promoted, and the opportunity for continuous quality improvement for care of the dying in an international sense is a tangible prospect’ (p.159). It was a claim made, however, on the feedback from just a couple of dozen health professionals.

A further study, already mentioned, from the palliative care unit of the department of medical oncology at the Erasmus MC-Daniel den Hoed Centre drilled down into how the LCP was working in the Dutch context, using an anonymous retrospective audit methodology ^ix^. Here the aim was to assess experience in the new setting and compare it with a matched group of patients in Liverpool, cared for using the LCP in a free-standing hospice environment. The choice of contrasting settings (hospital, non-hospital) is not explained, but there were similar results across a number of important dimensions, with most care goals being met for the large majority of patients. LCP was activated however in only 50% of those who died in Rotterdam, compared to 85% in Liverpool.

In Germany, the Ev Hospital in Oldenburg was the first to use the LCP. The setting was a specialist palliative care unit. From May 2007, the LCP was deployed over a period of five months, using a version that had been prepared for use in St Gallen, Switzerland, under the leadership of Professor Steffen Eychmüller. In a paper, which includes Eychmüller as a co-author, Simon *et al.* ^x^ describe the results of a focus group with 10 members of staff on the ward, conducted after 24 patients had been cared for using the LCP. During this period, a total of 36 patients died in the palliative care unit; the 12 who were not placed on the pathway all died suddenly. All members of the palliative care team were invited to participate in the focus group (10 nurses, three doctors, a caregiver, a social worker, a physiotherapist, an art therapist), but just seven nurses and the three doctors took part. All the participants had experience of looking after patients (the average was seven) on the LCP.

The results of the discussion are described at length in the paper. Overall, the participants were extremely positive about LCP. Echoing the discussions in St Gallen, the timing of when to start LCP (‘diagnosing dying’) was described by the focus group participants as an intense and important process. As ‘Nurse 5’ put it, the ‘moment when we decide to start with the LCP, now that is somehow a very special one and reconsidering it consciously, is something I experience as very positive’. After this decision is made, and based on open exchange in the team, all further measures could then be coordinated together, for example: the discontinuation of investigations or therapies that are stressful and unnecessary for a dying person; the use of on-demand medication for common symptoms; or the support of relatives.

Participants reported unanimously that the LCP enhanced communication between nurses and physicians, which in turn encouraged patient interaction and family caregiving. The structure of the LCP provided reassurance (especially around shift hand-over) that everything was being thought through and essential questions clarified. The flowcharts for drug-related symptom control attached to the LCP were found especially helpful for younger doctors with less experience in the field of dying. Likewise, the schematic structure of the LCP was a positive attribute, as the objectives to be achieved were well explained. Practical hints, such as informing the family doctor about the patient's situation were also described as helpful and as something that could often be forgotten in daily practice.

At the same time, there were some concerns that ‘dying people would be ticked off’ (Doctor 3) and that individualised care would be threatened. Working with the LCP was initially considered more time-consuming but despite that, the staff felt better, ‘because everything had been thought of and you were not just drifting’ (Nurse 4). Importantly, the participants also felt that the LCP was well-suited for staff on wards that are less likely to care for dying patients, as it provides a checklist to think about everything in this situation, particularly if back-up was also available from a specialist team: ‘The LCP provides an opportunity to ensure a certain basic care for the dying’ (Physician 1).

The authors of the Oldenburg study concluded that the LCP is a helpful and pragmatic tool for implementing palliative care in everyday clinical practice, but must always be supplemented and accompanied by qualitative guidance and palliative care training. There were however some limitations to the study, noted by the authors. First, only one focus group was conducted, with just 10 professionals, which could limit the scope of the results, albeit most of the team took part. Second, the LCP was implemented in a palliative care unit, though the target is the general ward of a hospital. Third, the study provided only the impressions of the health professionals involved in the pilot and did not provide evidence of the measured effects of implementation.

Colleagues in Argentina and Spain worked together on the use of LCP in palliative care services in the two countries and described the LCP translation and implementation processes and the initial piloting with 60 consecutive patients in two hospitals and one palliative home care setting and then the subjective perceptions of health professionals before and after the introduction of LCP ‘in a Latin American cultural context’ (Tripodoro 2013:2) ^xi^. Their focus was on the meanings assigned by professionals to the care of the dying, and on communication, teamwork, documentation, and particular attitudes. Here, LCP (Version 12) had been re-named, as in the Netherlands, but now with a much more culturally specific acronym: PAMPA (Program Asistencial Multidisciplinario Pallium). The study had two components. The first comprised a focussed ethnography within a hospital based palliative care team that had started training in PAMPA. The second comprised a questionnaire survey about professionals’ views on the implementation of PAMPA in Argentina (n=112) and Spain (n=23). The ethnography revealed favourable expectations about the of the value of LCP, doubts and fears concerning its applicability, and an acknowledgment of the role of intuition in end of life interventions. The survey respondents in both countries demonstrated high agreement on the choice of quality of care indicators (73.7% in Argentina, 91.4% in Spain), despite the fact that neither country had a national plan for palliative care from which such indicators could be drawn.

In Argentina, a paper (discovered after our analysis here was concluded) reported on the use of PAMPA in five health centres, where between 2008 and 2018 a total of 1237 adult patients in the last days of life were included and cared for by palliative care teams trained in PAMPA. The median range of follow up in the five centres from the beginning of the pathway until death varied from 16 to 178 hours. It was concluded that PAMPA demonstrated its feasibility as a model of end of life care for patients and families, based on international quality standards ^38^.

Dedicated Ministry of Health funding for LCP implementation was made available to all 20 New Zealand Health Boards from 2008, prior to which some Boards had already shown interest in LCP. By these means LCP was adopted to some degree across four settings in New Zealand – hospices, hospitals, aged residential care facilities and patients’ own homes. But as early as 2007, staff at Arohanui Hospice, the recognised lead collaborating centre for LCP, had begun to recognise some inconsistencies in how LCP was being implemented around the country. These included: a lack of consultation with specialist palliative care services, inappropriate and sometimes unsafe symptom management algorithms, the absence of general practice teams from LCP education and training, variability in LCP registrations, and the development and use in some places of modified, non-compliant LCP documents. Thus informed, the Arohanui Hospice made a successful bid to the Ministry of Health to establish a national co-ordinating office to oversee LCP implementation in New Zealand, with support from the Liverpool team. The goal was to develop a robust support infrastructure that would minimise the risk of the kind of *ad hoc* implementation and dissemination of LCP that would dilute and compromise its effectiveness and sustainability over time. The core approach to achieving this was the 10-step continuous quality improvement programme, developed by LCP Central in Liverpool. New Zealand was thus the first country outside the UK to formally establish a National Office with responsibility for promoting the sustainable implementation of LCP within its own borders. A paper by Mackenzie *et al.* ^xii^ presents the results of a mixed methods study to evaluate the role and value of the New Zealand office, from the perspective of key stakeholders, and also provides useful context on the local adoption of LCP.

Data collection for the evaluation took place in 2009, just six months after the New Zealand LCP office had been established. Committed to principles of dependability, credibility and trustworthiness, the evaluation was designed to provide useful information to inform development. It drew on the perspectives of a purposive sample of key stakeholders across New Zealand through interviews (n=28) and questionnaire surveys (n=36). The results were positive. The goals of the LCP office were deemed important, the service quality was rated good or very good, its ongoing links with LCP Central were considered important, it was leading to better quality use of LCP by linking closely with local facilitators in ways that connected theory to practice, and it was serving as a voice for palliative care in New Zealand. The authors concluded that the New Zealand office was proving successful in mitigating the risks of LCP implementation in a country ‘geographically isolated and culturally distinct from the UK’ (p260).

In Sweden there was a substantial engagement with the LCP initiative, albeit in a series of publications that did not begin appearing until after the withdrawal of the LCP in the UK. Ekeström and colleagues ^xiii^ sought to explore family members’ experiences in a palliative care unit and in a general geriatric ward in Sweden, before and after implementation of the LCP, which had first been introduced into Sweden in 2007 as part of a national project monitored by a palliative care competence centre, with the documents translated according to EORTC guidelines and implemented in collaboration with LCP Central, in Liverpool. The study places a particular focus on the perceptions of family members relating to LCP, citing only a few examples of this from elsewhere in contrast to the large number of studies on the perceptions of staff. The design was a non-controlled, before-after evaluation of the impact of LCP on family members’ experiences in a palliative care unit and in a general geriatric ward, with special attention to the goals of the intervention.

The settings for the study, each of which had introduced the LCP in 2009, were in the urban area of Stockholm and data was collected by means of self-complete postal questionnaire sent to a relative 3–6 months after the patient’s death. In total 108 family members agreed to participate (85%) and response rates and the before/after numbers were roughly equal across both clinical settings. Satisfaction with care was high in both settings pre-implementation, and family members were confident that staff had done everything possible to prevent suffering. Satisfaction on measures relating to existential issues and information on bereavement support was lower in the hospital ward, where relatives also considered that the patient had been more likely to experience breathlessness in the last three days of life. Post-implementation, only one aspect of care showed better results and this was in the Palliative Care Unit, where physicians’ ability to listen to questions and requests had improved. But post-implementation family members were more likely to state that the patient was worried or anxious. The authors considered that more information may have made family members more observant of symptoms, hence the increase in reported anxiety.

In 2016, Høgnes *et al.* ^xiv^ used the three phases of LCP implementation as a research tool to assess Swedish healthcare professionals’ documentation of end of life care for people with dementia in nursing homes. The study made use of the three phases (initial assessment, continuous assessment, and after death follow up) as a framework to sort the documentation. The study did not concern the implementation of LCP, but focussed on 50 sets of nursing records and 50 sets of medical records relating to deceased patients with dementia in two nursing homes. Through the lens of the LCP, it revealed that the great extent of the documentation focussed on physical symptoms, with almost nothing recorded on existential issues or follow up with relatives after death.

A descriptive qualitative study also emerged from the implementation of LCP in the Skellefteå municipality of Sweden ^xv^. It complemented the main evaluation by examining care professionals’ experiences of using LCP in the residential care homes of the municipality. The work was conducted through five focus groups and two individual interviews, comprising a mixture of nurses and nursing assistants working in the care homes, as well as local GPs. The line of questioning focussed on how the participants had experienced using LCP, its influence on their practice, as well as their perceptions of its strengths and weaknesses and their views on using it in the future.

The participants considered that using LCP had fostered a team approach that increased individual confidence, and this had been generated through the supporting educational programme and the new way of documenting care. Agreement about whether the resident was dying could prove difficult to establish, and this was exacerbated by the short amount of time spent in the homes each week by the GPs (who took the decision in each individual case about LCP use). But there was also an enhanced sense of individualised care when using LCP. The paper-based document kept in the patient’s room provided a focus for this, but was also seen as less practical than the usual computerised record keeping system. LCP was seen to have fostered earlier and greater involvement of family members in end of life discussions with relatives. Care staff were also more attentive to the care environment and gave more attention to creating a sense of comfort and welcome for family members when a resident was dying. Appearing late in the LCP ‘cycle’, the authors were able to contextualise their study in the findings of similar work from the UK and elsewhere. They concluded cautiously that the LCP might be a useful tool for use in residential care homes and could increase attention to the goals of care, the individual needs of residents, and the involvement of relatives.

Another Swedish study by Andersson *et al.* ^xvi^ attended to family members’ experiences of care of the dying in residential care homes where the LCP was in use. A total of 15 family members of deceased residents took part, drawn from 10 different residential care homes. Interviews were conducted January-March 2014 and began with the question: ‘Please tell me about your experiences of your relative’s last hours/days of life’. Three themes emerged: 1) being confident in a familiar and warm atmosphere; 2) being involved *versus* not being involved in end of life care; 3) being consoled by witnessing the health professionals’ endeavour to relieve suffering. The results showed that family members (most of whom were daughters of the deceased) had an overall positive experience of the care provided, felt involved in the caring process and found that LCP clarified the decision about moving to end of life care, and gave structure to what was happening. Subsequent to the study the authors note (and in the post-Neuberger context), a new care pathway was developed and implemented ‘in the early and late phase of palliative care in Sweden, not only in EoL care’ (p200).

Also in Sweden, Olsson *et al.* ^xvii^ focussed on residential care homes and home care settings and examined the perceptions of nurses on the impact of quality of care resulting from use of the LCP. A total of 142 registered nurses and assistant nurses working in a single Municipality in mid-Sweden, where the LCP had been introduced in the period October 2011 – December 2013 and completed a structured study-specific questionnaire containing 50 items. Implementation of the LCP was considered to ensure systematic assessment and alleviation of patients’ symptoms and needs, though assistant nurses were more positive in their views. Both groups considered that communication with patients and families and the information exchange between team members was facilitated. The areas for improvement concerned psychological and existential support, as well as patient and families’ participation in care. Publishing their results sometime after the widespread withdrawal of LCP, the authors note the creation of a new pathway in Sweden, highlight the importance of education of staff if pathways are to be more than a ‘tick box exercise’ (p.1596), and in particular draw attention to the complexities involved in diagnosing dying, the need for flexibility and the importance of continually assessing the status of the patient.

We identified just one item (a conference poster) on the use of the LCP in Denmark, presented in June 2016, in Australia ^xviii^. Here the intervention was described as the ‘Danish modified edition’ (mLCP) and was presented as ‘a tool to provide relief of bothersome symptoms in imminently dying hospitalized cancer patients’. The primary endpoints of the study were relief of symptoms, and correlation between symptoms and use of medication. The chosen method was an audit of mLCP records from 45 cancer patients who died in the integrated Palliative Care Unit, of the Oncology Department of Roskilde University Hospital, Denmark in 2014 – so some time after LCP withdrawal in the UK. In total 77% of the dying patients experienced good and immediate symptom relief, whereas 14% presented partly refractory but manageable symptoms. The most common symptoms were pain (56%) and anxiety (42%). Time on the mLCP was on average 48 hours. There was no correlation between presented symptoms and time spent on mLCP, nor was there a correlation between presented symptoms during the first four hours and during the last four hours before death. There was a significant relief of pain using syringe drivers, and a significant correlation between the use of analgesics and symptom relief. The authors concluded that integrated care pathways for best care of the dying person could be a valuable tool for providing good symptom relief.

Kaori Ichihara, a doctoral researcher at the Medical School of Osaka University, reported evidence of the use of what had come to be known as LCP-J (see below in the *Commentaries and other publications* section) in two Japanese palliative care units ^xix^. Ichihara’s article illustrates a whole process of LCP-J implementation, including training for practitioners, collecting information from patients and operationalising the pathway. Forty nurses were involved in the experiment and more than half considered the LCP-J useful, believing that it could contribute to providing standardised criteria for multi-disciplinary healthcare teams, as well as developing consistent and continuing care and support for patients and their families. Educational outcomes for healthcare professionals were also highly praised.

Kanno *et al.* (2015) ^xx^ report on a study to examine the burden of LCP-J when introduced onto two wards (oncology and respiratory medicine) in Tohoku University Hospital. Making use of audit data and interviews with two doctors and eight nurses, the study found that in a series of 22 patients placed on the pathway (38% of the total study group), there were no significant differences in the medications used in the two groups, but benefits were seen in a more structured approach to preparation for and care in the dying phase. At the same time LCP-J was felt to increase the burden on professionals in relation to the task of diagnosing the dying phase and the need for associated training. The authors concluded that the requisite support and training should come from the hospital palliative care team.

In Italy, the LCP studies were mainly conducted by a team of researchers and clinicians associated with a regional palliative care network of the National Cancer Research Institute and located in Genoa, Italy. The team was led by Dr Massimo Costantini in a three-year programme of research funded by the Italian Minister of Health from 2006 to determine whether the implementation of LCP in a hospital setting could be effective in improving end of life care for cancer patients. To this end, LCP version 11 was first translated into Italian for hospital use in 2007 and for hospice use in 2009.

An initial paper by Costantini, Beccaro and di Leo (2011) ^xxi^ draws attention to the issue of improving end of life care for patients dying in hospital, and their families. It refers to continuous quality improvement programmes as a vehicle for achieving this, highlighting the development of care pathways in this context, and singling out LCP as ‘the most structured and proficient’ (p.229) example of its type. Noting its use in over 20 countries, the authors also acknowledge that evidence for its effectiveness remains weak. Crucially, and in contrast to all previous efforts in this regard, they adopt the MRC Framework for the Evaluation of Complex Interventions ^39^ as the organising principle for their endeavours, in a focus on cancer patients dying in the hospital.

The researchers recognised that the LCP is a typical complex intervention, involving multiple components interacting with each other as well as with the local implementation setting. Accordingly, they first conducted a literature review to inform the development of the Italian approach, ‘Un percoso integrato per le cure di fine vita in ospedale’. They named their intervention LCP-I Program, and it contained 10 constituent steps that followed the continuous quality improvement programme recommended by the LCP Central team in Liverpool. Leo *et al*. ^xxii^ describe the process. After obtaining consent from the institution, steps 1–3 (‘Development’) involved evaluating the context, the development of the documentation and a retrospective evaluation of end of life care on the ward. As reported in other studies elsewhere, the translation followed EORTC guidelines. Steps 4–8 (‘Implementation’) involved a detailed programme of intensive training, the introduction of LCP with added support and coaching, evaluation of the education programme, and establishment of LCP as an indicator of quality for all dying patients. Through steps 9 and 10 (‘Sustainability’) the intervention was endorsed by the quality improvement programme and discussions were initiated about its wider use regionally and nationally.

LCP-I was led by an experienced palliative care team comprising two physicians, three nurses and two psychologists. This contrasts with the use of ‘facilitators’ in the UK. The Italian group placed a very strong emphasis on training, with a mandatory programme of 12 hours for ward staff prior to implementation. The setting was three medical wards (72 beds) and one respiratory disease ward (24 beds) of the Villa Scassi Hospital, in Genoa during 2007. In total over 400 deaths occurred on the four wards each year, about half from cancer. The implementation was assessed using a mixed methods approach. Pre- and post-implementation focus groups conducted with doctors and nurses showed perceived benefits, particularly in pain management and in communication with families. Process and outcome measures were tested using more quantitative approaches. The results were encouraging and began to demonstrate the possibility of undertaking studies aimed at assessing complex interventions in end of life care.

Di Leo and colleagues describe the carefully designed staff focus groups held at the Villa Scassi Hospital. Two focus groups with nurses and physicians were conducted both at step 4 and at step 8 and were moderated by a psychologist. A researcher took field notes at each session and audio recordings were made. Although the groups were less well attended than planned, the results indicated that the LCP-I Program may have improved the subjective perception of participants’ knowledge on the management of physical symptoms, awareness of emotional problems and information needs in end of life care, and matters of communication between medical and nursing staff. Nurses perceived some resistance to change on the part of medical staff as a barrier to the introduction of LCP-I. All participants became more aware of their own limitations in communicating with patients and families, in ways that heightened their own uncertainties – an outcome seen by the authors as potentially positive and as a first step towards improvement. Overall, there was significant agreement that the implementation of LCP-I had improved and intensified communication between the medical and nursing staff on the ward. The researchers were reassured that, although undertaken in a different culture to the one in which LCP was first developed, LCP-I appeared to be acceptable and valid in the Italian context.

The Genoa team then engaged in careful methodological testing of how the intervention could be evaluated ^xxiii^. This involved an uncontrolled before/after intervention trial within the four hospital wards at the Scassi Hospital and included all patients age 18 and over who died of cancer on the medical wards in the four months before and after the introduction of LCP-I (two months before and after on the respiratory ward). The ‘intervention’ group included those patients who had been assigned to LCP-I, as well as those that had not (though none of the papers give details of the numbers in each category or distinguish between them in the analysis). This was described as an ‘intention to treat’ methodology, which means all patients are included and are analysed in the groups to which they were located. The researchers used a quasi-experimental before/after design characterised by two measurement points, one before and one after the intervention, and without any external control group. They acknowledged that this design has the potential to exaggerate the effects of the intervention. The researchers contacted the informal care giver most closely involved with the patient, two months after the death, and invited them to take part in an interview. Quality of care was assessed using a Toolkit of items developed in the USA by Teno *et al.* ^40^ and translated into Italian and which measured the extent to which care at the end of life met the expectations and needs of the dying person and the family members; deriving a total score from 0 (poorest) to 100 (excellent). Some items from the Italian version of the post-bereavement survey, first developed in the UK and known as ‘VOICES’ ^41^, were also used.

A total of 115 patients was identified, 65 before and 50 after LCP-I implementation; four of these were excluded as they were related to staff members on the wards, leaving 111. There were differences in the under-lying characteristics and clinical dimensions of the eligible and the assessed samples, notably fewer cancer deaths in both the eligible and the assessed ‘after’ group, perhaps due to a temporal effect. There were also differences in carer compliance at assessment (interviews in the pre-intervention group took place longer after the death) and in the characteristics of the interviews (post-implementation interviews were significantly more likely to be by telephone), suggesting a selection bias and interviewer variability. In addition, the researchers identified a cluster effect associated with patterns of scores on the toolkit scale that correlated strongly with particular wards in the four that were included in the study. They concluded that the design they adopted had substantial limitations, and noted how this was reflected in the 2010 Cochrane Review of end of life pathways, which indicated potential benefits, but could not ascertain measureable effects from the available studies ^42^. At the same time, they were encouraged that it had proved possible to implement LCP-I, that staff had responded positively to the programme, and that insights had been gained for a future, and more robust evaluation. Nevertheless, the question remained whether this intensive method of LCP implementation, focussed on a single hospital and just four inter-related wards, could be replicated at scale across multiple settings.

The results of the pre- and post-trial relating to the experiences of family members and the possible effects of LCP-I were reported separately ^xxiv^ in a paper using the term ‘cluster phase II trial’ in its title, denoting that each hospital ward in the study constituted a ‘cluster’. An interview with a family member was obtained for 46 (73%) of the pre-intervention patients and 33 (69%) of the post-intervention patients. The second group showed higher scores on four out of seven dimensions of the Toolkit, relating to: respect, kindness and dignity; family emotional support; family self-efficacy; and co-ordination of care. There was no improvement in scores relating to symptom control. 

The group also explored the potential for LCP-I outside the hospital, in the context of the Italian in-patient hospice ^xxv^. Now an adapted version of the LCP-I, with approval from LCP Central, was introduced into seven hospices from three Italian regions, where the proportion of patients who died on it ranged from 36–89%. The interpretation by staff of its value varied widely across the seven settings: two hospices reported a positive impact, two took the opposite view; in three others, opinions were mixed. There was an overall concern about the lack of knowledge to underpin the use of LCP in the hospice and also about the methods of implementation that had been used.

There was also a comparative assessment of the feasibility of the pre- and post-implementation research design in the two settings of hospice and hospital, reported in a methodological article ^xxvi^. Here the primary aim was to evaluate the feasibility of using a combination of assessment methods, directed at different respondents, to create a measure of the quality of end of life care. The two cluster trials took place in eight hospitals and five hospices. Only cancer patients were included in the analysis. Overall, the method seemed to work effectively in both settings, with high levels of compliance and adherence to the study instruments. But the main reservation related to the use of proxies (rather than patients) as the main data source, ‘with all that this entails’ (p.6).

Another study examined the views of staff involved in the hospital implementation of the LCP-I, and who had shown reservations about it ^xxvii^. It was claimed as the first of its type. Six nurses and five physicians from six out of eight hospital wards that had used the LCP-I were interviewed. The authors themselves confirmed that the eligibility criteria for inclusion in the study were subjective and not insufficiently detailed. They identified ‘real’ concerns with the pathway but were also said to have identified mistaken interpretations of LCP-I among the respondents. Conducted before the Neuberger review had been reported, the authors took the view that their results were nevertheless similar to concerns raised by Neuberger.

Verhofstede and colleagues ^xxviii^ broke new ground by addressing the effects of the LCP in older patients and at the same time sought to develop a new programme of care for those dying in acute geriatric wards in the Belgian context. Their work began before the publication of the Neuberger review of the LCP, but in some ways foreshadowed its outcomes. They started by conducting an overview of LCP programmes in the UK, the Netherlands and Italy. This led to the identification of three common elements: the LCP document (and here the authors identify the substantive changes made in the translation process from the relevant English language version into Dutch); the supporting documentation; and the implementation guide. Then a literature review of successful LCP implementation strategies revealed five key factors: the importance of a dedicated training facilitator; the provision of initial and ongoing training; the organization of an audit and feedback opportunities; a central co-ordinating office to support local LCP facilitators; funding and available staff time. This led to an analysis of the concerns raised about the use of the LCP in the UK, specifically 1) improper or poor implementation leading to inadequate care, 2) unacceptable communication with patients and carers, 3) the ‘tick box’ orientation, and 4) the use of the term ‘pathway’. The understanding developed from these actions was then used to model a care programme for the last days of life among older hospital patients. This comprised a care guide, supporting documentation, and an implementation guide. The care guide involved translating LCP Version 12 into Flemish and then comparing it with the Dutch translation. ‘Liverpool Care Pathway’ became ‘Care Guide for the Last Days of Life’, thereby dropping the protocol orientation of ‘pathway’. Adaptations were made to the care goals, with older hospital patients as the focus. In addition, the Care Guide was made shorter than the LCP. Colour highlights were also introduced to improve readability of the care goals.

The authors located these elements of activity in stages 0-1 of the MRC Framework for the design and implementation of a complex intervention ^43^. The paper concluded with the intention to proceed to phase 2 – to evaluate the feasibility of the implementation. As described, it was a process strikingly different to those which shaped the original development of the LCP in the UK. 

Controlled studies

We identified just seven studies of this type, from just three countries: three each from Italy and Belgium, and one from Sweden.

The earlier work in Italy had provided enough evidence to justify the use of a randomised trial to evaluate LCP-I effectiveness. The team had then published a protocol for their new design ^xxix^. They argued that the ‘only feasible method’ to adopt was a cluster trial, where hospital wards are randomised to receive (or not receive) the intervention. This constituted a Phase III trial within the MRC Framework. In a departure from their previously highly localised work, they proposed that the intervention and control wards should be made up of ‘pairs’ from participating hospitals, which would be drawn from regions across Italy. The chosen patient group would remain those with a diagnosis of cancer. Inclusion criteria were carefully defined, the primary end points and sample size were described, and a detailed account was given of the administrative and implementation arrangements. The study was approved by the National Cancer Research Institute of Genoa in September 2009.

The key paper, published in *The Lancet* on 18 January 2014, was that which presented the results of the RCT in the 16 hospital wards, involving 147 patients who had been cared for on the pathway and 161 from control wards, who had received ‘standard care’; all had died from cancer ^xxx^. The results showed no differences between the intervention and control groups in relation to the overall quality of care (the primary endpoint). But two of nine secondary outcomes reported by family members showed better results in the intervention group – improvements in respect, dignity and kindness and in the control of breathlessness. The authors acknowledged that the study was under-powered – they had enrolled just 80% of the planned hospitals and slightly over-estimated the expected number of deaths. There were no differences in the medicines prescribed to the two groups, in the survival time after admission to hospital, or in the management of symptoms like pain, nausea and vomiting. In general, the beneficial effects were lower than in the phase 2 trials in the Netherlands and in Italy. But the die was cast. Neuberger had reported the previous summer and the verdict was that LCP should be discontinued ^xxxi^.

As in Italy, the work in Belgium included the development and publication of a protocol for an intervention study ^xxxii^. The design was again that of a cluster randomised control trial, to be conducted in 10 hospitals in the Flanders region, each with one or more acute geriatric units. In year one a baseline assessment would take place of usual care, based on completion of a questionnaire by relevant physicians, nurses and family members concerning each patient who died on the unit. Thereafter the hospitals would be randomised to receive the intervention, or not. This cluster randomisation, operationalised at the ward level, was thought likely to reduce the potential for contamination, since the comparisons would be between hospitals with and without the intervention. Subsequently in the intervention hospitals, the new Care Programme for the Last Days of Life would be implemented over a period of six months. A post-intervention assessment would be performed immediately after the baseline assessment in the control hospitals and after the implementation period in the intervention hospitals. The primary outcomes to be measured were symptom burden and frequency among patients in the last 48 hours of life.

A process evaluation was also proposed to assess the quality of the implementation of the new programme, to which the Belgian researchers then turned their attention ^xxxiii^. Their aim was (1) to determine the feasibility of implementing the Care Programme for the Last Days of Life in the acute geriatric hospital setting, and (2) to explore health care professionals’ perceptions of the effects of the Care Programme on end-of-life care. They undertook a phase 2 mixed methods study, according with the MRC framework, in the acute geriatric ward of Ghent University Hospital between 1 April and 30 September 2013. The approach included observation, interviews and the use of a quantitative tool, which measured the success of implementation using several indicators, such as whether a steering group was formed, whether and how many of the health care staff were informed and trained, and how many patients were cared for according to the Care Guide for the Last Days of Life. The process evaluation tool showed that implementing the Care Programme for the Last Days of Life in the geriatric ward was successful and thus feasible; a steering group was formed consisting of two facilitators, health care staff of the geriatric ward were trained in using the Care Guide for the Last Days of Life, which was subsequently introduced onto the ward and approximately 57% of all dying patients were cared for in accordance with it. Nurses and physicians experienced the Guide as improving the overall documentation of care, improving communication among health care staff and between health care staff and patient/family, and improving the quality of end-of-life care. Barriers to successful implementation of the Care Programme included difficulties with the content of the documents used within the Programme and the low participation rate of physicians in the training sessions and audits. The results were encouraging and suggested feasibility and favourable effects. Based on the identified barriers during the implementation process, the authors were able to make recommendations for future implementation and to further refine the Programme before its deployment in a phase 3 cluster randomized controlled trial for the evaluation of its effectiveness.

Again echoing the Italian study and with Costantini as a co-author, the results of the trial were published in *The Lancet* in 2017 ^xxxiv^. The authors included online a 22-page supplementary appendix to the published paper. The Care Programme for the Last Days of Life now had an acronym – CAREFul. The Belgian authors noted that in a Cochrane review of end of life pathways, updated in 2015, the Italian study had been judged to be statistically under-powered and at high risk of bias, mainly because patients were not masked to treatment allocation and there were high levels of attrition. The Belgian study avoided some of these weaknesses, though again the nurses knew about and took part in the CAREFUl intervention with the patients for whom they gave ratings. Nevertheless, the results still seemed equivocal. There were 118 patients in the control group and 164 in the CAREFul group who were eligible for assessment. Of these 92% and 80% respectively were assessed by nurses; but only 19% and 29% respectively were assessed by family members.

Nurse assessed scores were significantly increased by CAREFul, but there were no significant differences between the intervention and control group in family members’ ratings. The study also found a negative effect on satisfaction with care among family members, described as ‘a serious concern that needs to be investigated further’. The authors concluded that their results ‘suggest’ implementation of CAREFul - an intervention based on the LCP - ‘might’ improve care during the last days of life for patients in acute geriatric wards. In an accompanying editorial, Aslakson and Lorenz ^44^ praise the ambition of the study, but home in on the involvement of nurses as both the unmasked study interventionists and the study assessors, creating a high risk of unconscious bias leading to more favourable reported outcomes. They conclude that the results of the CAREFul study are welcome ‘but critical questions remain unanswered about the study itself and the contextual and implementation issues that the experience with LCP revealed’ (p98).

A randomised study of an end of life pathway had therefore been conducted in both Italy and Belgium, and published in a world class medical journal, but in both instances the results were equivocal.

Researchers in Sweden recognised the lack of controlled studies of LCP outside of cancer settings and focussed on its use in care homes and in residential care homes ^xxxv^. In Skellefteå municipality, they assessed the effects of the LCP on patients’ symptom distress and wellbeing, when compared to usual care. The design was an exploratory, controlled before and after study. During a 15 month baseline period (June 2009 – August 2010), usual care was assessed in two areas of the municipality, containing 10 and 9 care homes, respectively. In the following 14 months, staff introduced LCP in one area and usual care continued in the other. A translation of LCP Version 11 was used, in collaboration with the Swedish LCP co-ordination centre based at Stockholm Sjukhem, and the process included a structured education programme to support the implementation. In both areas in the pre- and post-intervention periods, care was assessed through the completion by relatives after the resident had died of two structured instruments (Edmonton Symptom Assessment System and Views of Informal Carers – Evaluation of Services, known as VOICES), sent by post. The study covered residents (referred to by the authors as ‘patients’) who died in all 19 residential care homes in one Swedish municipality.

A total of 837 patients died during the whole study period. The LCP was completed for 132 (60%) of those who died in the intervention area; two thirds of the remainder died suddenly in the care home or died in hospital. Cardiovascular disease and dementia were the main causes of death. The total response rate for completion of the questionnaire was 46.2%, there was a significantly higher response rate in the intervention area. The main result was a reduction in two symptoms among residents in the intervention group – shortness of breath and nausea. Reductions in both of these symptoms were reported by the symptom assessment measure and for shortness for breath only by the carer evaluation tool. On such a basis, and following a detailed reflection on the practical and technical limitations and potential biases of the study, the authors felt able to conclude that the LCP may be a useful tool for providing end of life care for elderly people in non-cancer settings. Interestingly they note that, based on the positive experience of conducting the study (as much as its outcome), the municipality involved made a policy decision to implement the use of LCP in all its residential care homes.

Commentaries and other publications

Beyond these audit, implementation and controlled studies we identified 20 commentaries of various kinds and also nine miscellaneous pieces that could not easily be classified – almost a third of the total number of publications on the use of the LCP outside the UK.

The early Dutch papers set the scene for the introduction of LCP into the Netherlands, the link with Ellershaw and the processes being adopted, including the translation-back translation approach ^xxxvi– xxxviii^. By 2012, Geijteman, Dekkers and Zuylen ^xxxix^ could observe that 119 institutions, including 35 nursing homes were working with the LCP in the Netherlands and that a digital version of the LCP was under construction, which would make it possible to integrate it in electronic patient files, as well as serving the purposes of quality assurance and scientific research. 

A paper by Raijmakers *et al*. ^xl^ acknowledges the withdrawal of the LCP in England and the rest of the UK and also gives a detailed account of the wider ‘roll out’ of LCP in the Netherlands. This was built on the perceived positive research results in the pre and post implementation Dutch studies. Critical to the scaling up was endorsement of the LCP by the Comprehensive Cancer Centre of the Netherlands (CCCN). This led to ‘roll out’ in 66 regional palliative care networks – groups covering specific geographical areas and committed to ‘intensive collaboration and synchronisation’ (p.260). This had involved advocacy for the LCP, training programmes of two days’ duration for project leaders, and support on implementation through a helpdesk.

Raijmakers *et al.* also note that implementation research to bridge the gap between evidence and practice in palliative care is not widespread. They decided to address this through a study of LCP implementation by interviewing CCCN consultants involved in LCP training in each of its eight regions. These people were asked to nominate organisational examples of successful implementation, 10 of these led to follow on interviews with project leaders from 25 examples given. Perspectives from one palliative care network from each of the CCCN regions were also obtained and all interviewees in the study were invited to take part in a summative focus group by way of conclusion.

No studies of LCP or similar pathway implementation were identified for Ireland, but one case note review, given ethics approval by the Beaumont Hospital, Dublin, focussed on dementia patients during acute hospital admission, and used LCP as a standards benchmark for quality palliative care in a comparison with patients who did not have dementia ^xli^. Data were collected between January and June 2008 on a total of 50 patients, a condensed version of the LCP was used to focus on nine aspects of good quality care – rationalization of medicine, discontinuation of unnecessary invasive interventions, prescription of palliative drugs, referral to specialist palliative care, documentation of resuscitation decisions, communication with primary care, supporting caregivers in bereavement, and assessment of religious and spiritual needs. The researchers found that whilst both groups of patients had similar levels of invasive treatment, those with dementia were less likely to be referred to palliative care, were prescribed fewer palliative medications, and had less involvement of relatives in dialogue about treatment. The authors drew no conclusion about the value of LCP implementation, in a study where it was used simply as part of the research design.

As late as 2012, a reflective piece by a Swiss internist on experiences of palliative care concluded that, among a list of shortcomings, there was still a lack of standardization across settings of care in the final phase of terminal care, and used comparison with the guidelines contained in the LCP as an indication of this ^xlii^.

Around the time of publication of the Oldenburg study, in 2009, the German journalist Florian Rötzer, wrote a critical article for *Heise Online* about the LCP, entitled ‘Sentenced to death by palliative care? Physicians warn of a guideline for the care of dying patients’ ^xliii^. Rötzer could see the value of clinical guidelines for end of life care. These would avoid acting arbitrarily or criminally *,* provide security to medical staff and advice on what to look for while helping the dying to leave life in a dignified and pain-free way *.* But taking his cue from controversies emerging in the UK, he argued that the LCP also has the potential to be dangerous in a context where the matter of diagnosing dying can be imprecise. This could mean nothing short of the potential for a ‘national crisis’ in which relatives witness the discontinuation of treatment, apparently driven by economic expediency. The remarks neatly anticipated the subsequent events and discussions in England.

It was then four years before further work was published on the use of the LCP in Germany, in a textbook chapter by Voltz, Nübling and Lorenzl ^xliv^ on care of the dying for neurologic patients, which set out the rationale, development and perceived benefits of LCP and asserted that whilst LCP had been withdrawn that year in the UK, it remained a good model for structured end of life care.

Meanwhile, commentary on LCP emerged from Austria in 2013 in the form of a published undergraduate dissertation in advanced nursing practice, by Zinner ^xlv^. Her focus was on how the LCP could be implemented in hospitals in the German-speaking area and how the quality of life of the dying can be preserved or improved by using the LCP. Zinner identified a nursing textbook chapter on the LCP by Müller ^xlvi^ that describes LCP version 11 as a well-functioning, multidisciplinary procedure and a very useful tool in caring for the dying. Addressing repeatedly voiced fears that guidelines for care at the end of life run the risk of ‘standardising’ dying, Müller asserts that any guideline or tool can only be as good as its users (Müller, p. 102, quoted in Zinner 2013).

After the withdrawal of the LCP in England, senior clinicians from Australia commented on the implications for the continuing use of end of life pathways in their country (Chan *et al.,* 2014) ^xlvii^. They acknowledged that, across Australia, several end of life care pathways had been adapted from the LCP, though with no precise numbers of the institutions involved. In particular, they highlighted the recommendation for a national roll out of an integrated end of life care pathway through primary, acute and aged care sectors, that had been included in the Australian National Palliative Care Strategy of 2010. Despite the widespread experimentation with and policy endorsement of the end of life pathway in Australia however, the authors took the view that (as elsewhere) there had been insufficient evaluation of its net effect. The withdrawal of LCP in the UK had created a dilemma for Australia. The authors made a plea for more rigorous, randomised, studies of end of life pathway outcomes and implementation, but acknowledged that this would take time. Meanwhile they recommended that the shortfalls and adverse effects identified in the Neuberger review should be carefully assessed in the Australian context, and concluded that ‘if the LCP is to be replaced, there needs to be systematic measurement of the benefits and harms generated by such a process’ (p573).

Norway’s part in the LCP narrative includes reactions to it which took place after the recommended withdrawal in the UK in 2013, though it was not until 2016 that the commentary emerged in a rapid review produced by the Norwegian Institute of Public Health and requested by the Norwegian Directorate of Health ^xlviii^. The authors explained the goals and background to the LCP and noted its ongoing use in Norway since 2005, though with no details of extent or setting. The aim of the review was to assess the effectiveness of the LCP and also its relevance to patients with dementia. Only two studies met the inclusion criteria (from Sweden, in 2014 and Italy, in 2015). The results (as we have seen above) showed small or no differences between LCP and standard palliative care at the end of life. The available evidence indicated that LCP possibly can improve breathlessness in dying patients, however there was no evidence that LCP was superior to standard palliative care with respect to quality of care, management of most of the dying patients’ physical symptoms, and prescription of palliative medications. At the same time the authors had very low confidence in the evidence they reviewed, mainly due to a high risk of bias in the studies, which also enrolled few participants and contained shortcomings with regard to the implementation of the intervention. For example, in the Italian study, only 34% of cancer patients in the intervention wards received LCP, and in the Swedish study, only 60% of patients in the intervention homes received LCP. The authors therefore noted that the various weaknesses of the available studies limit confidence in the then current evidence and restricted the potential to draw firm conclusions.

The following year a systematic review of the use of LCP in nursing homes was produced by a team in Norway ^xlix^. It included the provocative subtitle ‘discarded in cancer patients but good enough for dying nursing home patients?’ and described the history and purposes of the LCP, emphasising that the approach was designed for use with cancer patients and ‘presumed an open and timely communication between the treating physician, nursing staff, patient and relatives’ (p1). It noted that LCP was in use in 17 countries outside the UK, including Norway. Describing the media and public concerns that had arisen about LCP and which led to the Neuberger review, the authors observed that in contrast to the UK, no open and critical debate had taken place about LCP in Norway or other Scandinavian countries. The aim of the 2017 review was therefore to assess the evidence for the use of LCP, but specifically in the nursing home context and in relation to people with dementia. It focussed on how LCP had been validated and tested in these contexts, with which study designs and methods, the implementation strategies in use, and how they had been described, along with the main outcomes.

Twelve papers, one purely methodological, were selected for inclusion in the Norwegian review, based on nine clinical studies; seven studies were from outside the UK and are discussed elsewhere in the present paper. None of the selected studies used randomized, blinded or prospective designs. Only one was controlled. The reviewers concluded that evidence for the use of LCP in nursing homes was virtually absent and when present was weak and the results not definitive. Acknowledging the problems of randomised designs in these populations, the authors were nevertheless critical of the absence of alternatively designed studies that document the development and testing of the instrument by including elderly, multi-morbid patients and those with dementia. They concluded that the LCP had not been adapted to the individual needs of people in nursing homes and those with dementia. In Norway almost 50% of deaths take place in nursing homes, where 80% of patients have dementia. Husebø, Flo and Engedal close by stating: ‘After changing the name of the procedure, the LCP is still in use in many countries, as a low-cost camouflage of the real need for education and competence in nursing homes’ (p.12).

The one paper we located on LCP in China ^l^ took the form of a professional commentary and looked at the potential of LCP in relation to the development of hospice nursing in mainland China. The article explains how LCP was developed, reviews the research on its implementation and benefits, but also acknowledges the critical commentary on LCP that had occurred in the British press and from some clinical commentators. With reference to the experiences of the LCP in other countries, the authors argue for strategies to improve Chinese nurses’ knowledge of palliative care and also for consideration of how the advantages of the LCP and other models of care, could be used to develop a more appropriate model of palliative care for the Chinese context.

In a 2008 paper on the status of palliative care in Japan, a group of authors ^li^ commented that standardised frameworks, guidelines and clinical pathways for end of life care, available in the English-speaking world, had not been developed or were inadequately disseminated in the Japanese context. They indicated that a validation study of several such interventions was underway to modify the originals and make them suitable for Japanese culture. The list included the Liverpool Care Pathway. 

Two years later, in 2010, a special issue of the Japanese Journal of Clinical Nursing was published that focussed entirely on the use of LCP in Japan ^lii^. Edited by Mitsunori Miyashita, a professor in palliative nursing at Tohoku University, the special issue aimed to provide a comprehensive picture of the introduction and implementation of the LCP in Japan, at that time. The editor introduced the issue with some background on end of life care in Japan, addressing the importance of promoting and adapting the LCP to the Japanese context, and pre-viewing the main articles in the special issue ^liii^. This one special issue contributed eight of the 17 articles on LCP in Japan within our review, though most were commentaries of one sort or another. 

Karoi Fukuta from the Seirei Mikatahara Hospice offered an insight into LCP-J by reporting an individual case of a terminally ill cancer patient ^liv^. The implementation is recorded in great detail by showing how the patient and her family were supported at different stages of the dying process. By referring to the criteria of the LCP, the author provides a clear picture of how the patient’s symptoms were assessed and how the techniques of the LCP-J were employed to support her various needs and those of family members. The author concludes that the LCP-J improved the quality of palliative care for the patient and her family. 

The last article in the special issue pays attention to an evidence-based booklet produced for family members of dying patients ^lv^. Hiroyuki Otani, from the Kyushu Cancer Centre, argues that the booklet can help family members to better understand end of life care, reducing anxiety and empowering them to give improved support to their dying family member. The author also points out the benefits of using the booklet with the LCP.

A short paper by Yoshikazu (2016) ^lvi^ gives an overview of the rise and fall of the LCP in the UK, in relation to end of life care in Japan. It acknowledges the usefulness of the LCP as a guideline for end of life care practices and for the education of medical professionals. It then reviews how the LCP was introduced and developed in Japan from 2003, leading to the production of LCP-J which began in the same year. LCP-J and its manual were completed in 2009, and the home-care version, LCP-H was introduced in 2012. While highlighting the increasing influence of the LCP in Japan, the paper also documents the wider debates in the UK on the effectiveness and appropriateness of the LCP. Given the criticisms and the withdrawal of the LCP in the UK, the author suggests that the promotion of the LCP in Japan should stop and an alternative pathway that fits Japanese contexts needs to be developed.

In a further paper from 2016, Chinone ^lvii^, a palliative care doctor in charge of developing the Japanese version of LCP-J, also sets out the wider historical background of the LCP, its contents and some examples of its implementation elsewhere, before describing the reasons for introducing the LCP to Japan, why it should be adapted to the Japanese context, and the continuing possibilities for its implementation. 

Tanaka and Satoshi ^lviii^ in 2017 also provide an overview of end of life care in Japan, with a particular focus on developments in the UK, including the LCP, and their relevance in the Japanese context. Reviewing the debates surrounding LCP in the UK and its eventual withdrawal, the authors suggest that end of life care in Japan needs to 1) pay more attention to individualised care and communications, 2) develop education programmes as well as institutional and home-based services, and also 3) enhance the involvement of local authorities and communities for the benefit of patients and carers.

The Italian researchers Costantini and di Leo ^lix^ went on to describe the debate that took place in Italy, following the recommendations of the Neuberger report. They used the term ‘malpractice’ to describe some of the cases that found their way into the mass media in the UK, described and summarised the Neuberger report process and findings and noted the subsequent creation of the Leadership Alliance for the Care of Dying People in England, its aim of developing policies and processes to ensure high quality, consistent care for people in their last days, and its production of five priorities for care at the end of life. They observe that ‘The story of the LCP is also Italian’ (p266) and describe its development in the Italian context, as detailed here in our own paper. Early results in Genoa had been positive and supported the design of a cluster randomized Phase III study, to assess the effectiveness of the programme in improving the quality of care of end of life care for patients dying of cancer in Italian hospitals. Despite the standardized implementation process and carefully planned support from the palliative care teams, the findings of this study were less encouraging than the Phase II studies and did not reach significance for the primary outcome, but did demonstrate some secondary benefits. In light of all these factors, over 50 clinicians from six Italian regions came together to share opinions and experiences about LCP-I. A list of 12 strengths and 13 weaknesses of the LCP programme in Italy was generated. There was a strong emphasis on coping with the risks associated with the intrinsic limitations of LCP and its inappropriate dissemination. The group took the decision to ‘phase out the LCP in Italy’ (p266–67) and noted that the same approach would be taken in all countries where the LCP had been disseminated. But di Leo and Costantini observed ‘... the problem remains. The quality of care for the dying patients in hospital is suboptimal’. The LCP studies had demonstrated the feasibility of conducting high quality research in this field and this strategy would continue.

In the same year, 2014, Costantini, Alquati and di Leo published a review on the evidence for pathways in end of life care ^lx^. They acknowledged that two separate Cochrane reviews, last updated at June 2013, did not find any studies of end of life care pathways that met the inclusion criteria, though their unique Italian study, albeit with its negative results, post-dated this. In a somewhat ambiguous sentence in the abstract, they concluded that ‘the overall amount of evidence supporting the dissemination of end-of-life-care pathways is rather poor’ (p1741). Two major drawbacks could be seen in the analysis of the quantitative studies on end of life pathways. First, poor internal validity of design: uncontrolled before and after studies have intrinsic weaknesses and are vulnerable to changes in the external environment that might lead to improvement, they are at risk of the Hawthorne effect, regression to the mean and are likely to overestimate the effects of interventions. Second: the piecemeal character of the studies meant they lacked a comprehensive strategy for research, in line with the MRC framework for the evaluation of complex interventions. The pool of studies undertaken in Italy, and proceeding through the three phases of the MRC framework, seems to be the only example of such a comprehensive strategy, though the authors note developments in Belgium and Sweden (at the time unpublished, but described above here) that might merit inclusion in a future Cochrane review of end of life pathways. In noting the outcome of the Neuberger review, the Italian authors also observe that none of the published studies report any adverse effects, relating to patients, family members or involved professionals. They called for more appropriately designed studies of end of life pathways, as well as studies taking account of the LCP ‘debate’ and the outcomes of the Neuberger report.

***Conclusions to literature review.*** We identified 95 publications covering the period 2003–09 and referring to 20 countries in which LCP interest and implementation had been considered or tried.

Just over half (n=11) of the countries identified in the literature review produced three published outputs or less: Slovenia, Ireland, Switzerland, Spain, Singapore, India, Denmark, China, Argentina, Norway. These countries generated 18 outputs in total, making just 19% of the total reviewed. It is difficult to see how most of these countries reveal a high-level commitment to LCP transfer, with the exception of Switzerland, which was part of a three country German language group and Norway, where there was significant in-country take up of LCP. Beyond those, publications from these countries arose mainly from small scale developments, only occasionally based on wider collaborations and which had little impact on wider transfer or diffusion (for example colleagues in Spain working with others in Argentina). However, this is not to undermine the value of this work to the actors involved, who gained local experience of the translation of an intervention into a new context, who sometimes adapted it with imagination and flair to local cultures and healthcare systems, and who used it to audit and benchmark their own end of life services.

The remaining nine countries, just under a half of those identified in the review, produced 4-17 publications each: (Japan, Netherlands, Italy, New Zealand, Australia, Sweden, Belgium, Austria, Germany). This accounted for no less than 77 outputs, making 81% of the total. These were countries where, in some cases, LCP transfer gained significant momentum, sometimes operating at scale and in some instances being subjected to rigorous testing in robust research studies. This in turn produced impactful publications in international journals, greater visibility in professional conferences, and in the case of Japan, a journal special edition devoted to LCP.

The authors of the papers reviewed were overwhelmingly clinicians, some with a strong research orientation and holding senior academic positions. The leading authors were mainly medical, and several of them took part in our interviews (see below). There were few papers that involved nurses or other health professionals, and almost none that included social scientists or implementation experts. This is reflected in the character of the published outputs.

The largest single group of outputs, making one third of the total and completed in 15 countries, comprised descriptive audit studies, drawn from clinical records. These had often been undertaken following the guidance received from LCP Central in Liverpool. Some were baseline, pre-implementation assessments, others compared outcomes pre- and post- implementation.

After these, comprising 28% of the total and from 10 countries, were mixed methods studies that examined *acceptability* and *effectiveness* of LCP, drawing on perspectives from process or implementation analysis. 

Just three countries, making up only 7% of the outputs, produced controlled studies, of which only two were RCTs.

The remaining one fifth of the outputs comprised commentaries or descriptive pieces of some kind, all without a research design.

The nine countries with the most outputs, were also those that produced the most robust research results, albeit with only three of these conducting the sort of controlled studies that would stand up to critical review and be published in leading journals. The 95 outputs we reviewed were therefore long on commentary and the sharing of experience, or focussed mainly on descriptive audit methodologies, sometimes with added process measures to shed light on acceptability and feasibility. 

Only a tiny minority used rigorous designs, albeit with some flaws, and equivocal results. In Sweden the key study had modest results – a reduction in two symptoms in the intervention group – shortness of breath and nausea. In Italy, in an under-powered RCT, there were no overall differences in quality of care between the intervention and control group, though the latter, as described by family members, showed better results in the intervention group on two counts: improvements in respect, dignity and kindness and in the control of breathlessness. In the key Belgian study, there were generally no significant differences between the intervention and control group in family members’ ratings but a *negative* effect on satisfaction with care among family members was observed in the intervention group.

### Interviews

Table 2 lists the 19 interviews we conducted with 20 people from 14 countries. One interviewee subsequently withdrew from the study and is not included in the analysis. Six countries with some measure of LCP activity identified in the literature review were not included in the interviews. In Spain, Ireland and Hong Kong those we approached declined to participate. In Slovenia our desired interviewee was unavailable for interview. In China and Singapore, we failed to track down potential interviewees who could be approached. All interviewees agreed to be interviewed ‘on the record’ after signing the informed consent form and approving the full analysis of the interviews ( *Extended data* ^28^) for this part of the study; they are thereby identifiable in our reporting. Interviews ranged in length from 36 to 66 minutes. Only three interview participants (Boughey, Douglas, Medicus) were not among the authorship of papers we identified for the literature review.

**Table 2.** Interviewees by country.

Country	Respondents’ names and roles	Interview number
India (1 interview)	Dr Stanley C. Macaden, Palliative Care Consultant Physician, Bangalore Baptist Hospital and Christian Medical Association of India (CMAI)	1
New Zealand (1 interview with two people)	Dr Simon G. Allan, Consultant Medical Oncologist and Palliative Care Physician and Bridget Marshall, Clinical Nurse Specialist and National LCP Lead	2
Argentina (2 interviews)	Dr Vilma Tripodoro, Chair Palliative Care Department, Lanari Institute of Medical Research, Buenos Aires	3
	Dr Gustavo De Simone, Director Pallium Latinoamérica	6
Germany (1 interview)	Professor Raymond Voltz, Director of the Center of Palliative Medicine, University Hospital Cologne	4
Norway (1 interview)	Professor Dagny Faksvåg Haugen, Professor of Palliative Medicine, University of Bergen	5
Sweden (1 interview)	Professor Carl Johan Fürst, Professor of Oncology and Pathology, Lund University	7
Australia (2 interviews)	Associate Professor Dr Carol Douglas, School of Clinical Medicine, University of Queensland and Director of Palliative and Supportive Care Service, Royal Brisbane and Women’s Hospital	8
	Dr Mark Boughey, Deputy Director, St Vincent’s Hospital Melbourne, Centre for Palliative Care, Associate Professor, The University of Melbourne	12
Netherlands (2 interviews with the same person)	Dr Lia van Zuylen, Consultant Medical Oncologist, Department of Medical Oncology, Erasmus MC Cancer Institute, Rotterdam (From January 2020 Medical Oncologist and Professor of Clinical Palliative Care, Department of Medical Oncology, Amsterdam UMC	9 and 14
Japan (2 interviews)	Professor Mitsunori Miyashita, Department of Palliative Nursing, Health Sciences, Tohoku University Graduate School of Medicine, Sendai	10
	Dr Ai Oishi, PhD student, Primary Palliative Care Research Group, Centre for Population Health Sciences, Medical School, University of Edinburgh, and GP trained in Japan	11
Belgium (1 interview with two people)	Dr Tinne Smets, senior researcher, Vrije Universiteit Brussel (VUB) & Ghent University End-of-Life Care research group and Dr Kim Beernaert, FWO post-doctoral fellow at Ghent University and chair of the “Palliative care for people with cancer” Research Programme at the End-of-Life Care Research Group of the Vrije Universiteit Brussel (VUB) & Ghent University	15
Denmark (1 interview)	Dr Svend Saalbach Ottesen, medical oncologist and a specialist in palliative medicine, Roskilde	16
Switzerland (1 interview)	Professor Steffen Eychmüller, Institute of Social and Preventive Medicine, University of Bern	17
Austria (1 interview)	Dr Elisabeth Medicus, retired palliative medicine physician	18
Italy (1 interview)	Dr Massimo Costantini, Azienda USL-IRCCS of Reggio Emilia	19
Confidential	Interviewee withdrew from study	13

We set out six dimensions that resulted from our analysis of the interviews: 1) context and motivation for engaging with the LCP; 2) translating and adapting the LCP for a new context; 3) deployment and diffusion of the LCP within countries; 4) perceived benefits of the LCP; 5) challenges and drawbacks associated with the LCP; and 6) perspectives on the withdrawal of LCP in the UK and its consequences. 

***Context and motivation for engaging with the LCP.*** LCP adoption varied in its organisational focus, from specialist palliative care settings, to general hospital wards or care homes, but those involved shared a common enthusiasm for what they saw in the LCP as a structured approach to improve care of the dying, and they came to it through a variety of networks.

In Norway, Professor Dagny Faksvåg Haugen recalled the potential of the LCP as a means to optimise care and make quality less dependent on the individual practices of clinicians:

... there was really nothing new in the LCP. But it structured what we already did or wanted to do in a very good way. We saw it as a good checklist and a framework securing a certain quality of care … no aspect was forgotten. So we thought that providing this framework for clinical decision making provided a standard for good care. So it would be more uniform everywhere, not so much dependent on the individual professional. (Professor Dagny Faksvåg Haugen, Interview 5)

Interviewees from 10 countries recalled how they were drawn to the pathway because of pre-existing networks with the LCP team in the UK or the worth of its reputation, and especially the relevant publications that were emerging. Dr Svend Ottesen, an oncologist who had led a palliative care unit in Denmark between 2004 and 2015, recalled that his introduction to the LCP came through a specialist course in palliative care for the Nordic countries and then the opportunity in 2007 to attend a course on the LCP, in Liverpool.

For others, the involvement came through an international LCP interest group that took opportunities to meet at annual conferences of the European Association for Palliative Care and which, in 2008, broadened into a formal collaborative of nine countries known as ‘OPCARE 9’ and was funded by the EU:

Well, we met with the Liverpool Institute and John Ellershaw's group in 2008, because of participation in OPCARE9. You know the international research group? Argentina was one of the countries participating in the project with another eight countries, and we met the group on this occasion. So, since 2008 we start working on the best care of the dying, in this research group. (Dr Vilma Tripodoro, Interview 3, Argentina)

In Queensland, Australia, the genesis of the LCP introduction was described by Associate Professor Carol Douglas as a study in the Royal Brisbane hospital, where she was appointed Director of Palliative Care in 2006 and asked by the head of the hospital ‘to do something about the very poor state of dying in this facility’. She was subsequently approached by a senior clinical colleague (who had worked in London) to collaborate on a study mapping the last 24–48 hours of life of patients and leading to an adaptation of the LCP:

… that’s what first triggered my interest in having a framework to support junior medical staff and nurses. I was successful in getting some Commonwealth funding in about 2007, to try and develop a pathway for dying. (Associate Professor Carol Douglas, Interview 8)

In some cases, notably Italy, Belgium, Switzerland, the Netherlands and Japan, interviewees reported a research-oriented rationale to the introduction and assessment of the LCP. In Italy, Dr Massimo Costantini recalled first hearing about the LCP around 2008/9 at the same time as an opportunity presented itself to apply for research funding from the Italian Ministry of Health. He decided to join the LCP international reference group with the intention to immediately embark on a research study of the pathway:

…when I heard about LCP, to be honest I can’t remember who spoke to me about that, I looked for it on the web and I found the Liverpool group. I asked for information and they replied giving me information about that and saying that they didn’t have any reference person in Italy. I had to decide what to do, so my decision to join the international group was a consequence of my decision to start with a research trajectory in Italy because it was really clear to me that LCP had to be assessed before implemented. The evidence in my opinion was not strong enough to justify implementation without research. In the meantime, I joined the international group and I started the process of research in Italy. (Dr Massimo Costantini, Interview 19, Italy)

Interviewees from Belgium, whose main roles were as University based palliative care researchers rather than clinicians, also reported a primarily research-oriented rationale to the introduction of the LCP, and the experience of linking with other researchers elsewhere. In 2011, opportunities for palliative care research funding via the Flemish Government Agency for Innovation by Science and Technology resulted in resources for six palliative care studies. A cluster-randomised trial of an adapted version of the LCP (the CAREful intervention) was one of these. It focused on whether the adapted version of the LCP improved levels of comfort at the end of life among patients in geriatric wards in ten hospitals in Flanders:

… there was some evidence that [LCP] was effective in cancer patients but not in patients dying from other conditions and especially not in older patients, so I think they saw an opportunity to test it in this population and setting, and so in 2011 we started with this … talking to people in the Netherlands and also to Dr Costantini in Italy; we knew he was doing a big trial in hospital with cancer patients, so we went to Italy to talk to him and we went to the Netherlands to talk to those people and we also went to Professor Ellershaw’s group in the UK and we started with the various documents and the program and thought, well, what can we use, what is suitable, do we need to adapt this and how can we make this work in Belgium? (Dr Tinne Smets and Dr Kim Beernaert, Interview 15, TS speaking)

A strong research focus and rationale was also discernible in the accounts of Dr Lia van Zuylen from the Netherlands and Professor Steffen Eychmüller from Switzerland, in both cases building on their personal knowledge of Professor Ellershaw and his work.

I remember very well my first talk with John about this LCP. And I remember also very well that I went to the Head of our Department and that I had a discussion that we would like to work with this document … But he said, we don’t know if patients have benefit from it. In the UK there was already a big rush around implementation. But there was no scientific evidence of benefit. So the Head said, if you want to introduce this it will take time and energy. You have to know if it gives benefits for the patients. So that was the moment that I said, okay, now I have to do something else, I have to go into research and I was thinking about it because I was not familiar with this kind of research. (Dr Lia van Zuylen, Interview 14)

In Japan, Professor of Palliative Care Nursing, Mitsunori Miyashita described how his initial introduction to the LCP was related to his knowledge of an early attempt at implementation by a medical doctor, which had started in 2004. The attempt failed for two reasons: scale of the task and the difficulty associated with translation into the Japanese clinical context. This highlighted the importance of preliminary research testing. Professor Miyashita describes how he assumed the role of principal investigator of a pilot study in two in-patient palliative care units in 2008/9:

At first, Dr (name) was principal investigator. Then he discussed with the LCP centre team UK and proceeded to translate. But this project [did] not work well. The implementation was delayed because … one reason is … he’s a clinical doctor. He was so busy. The second reason is … it was difficult to agree with the translation especially on this algorithm … [and]…the usage of medicine … the progression was very slow. Then I entered the team. And I became principal investigator … in 2008 or 2009. Then I completed the translation and pilot… [and] did pilot tests at two inpatient palliative care units. (Professor Mitsunori Miyashita, Interview 10)

***Translating and adapting the LCP for a new context.*** In 11 countries the LCP required linguistic translation and a variable degree of cultural or contextual adaptation. In the other three countries (New Zealand, Australia and India), there was no need for translation, but other adaptations were necessary.

Interviewees gave variable accounts of their recollection of the translation and adaptation process. Their emphasis varied from a focus on precise and exact translation of the UK documents (often referring to the use of translation guidelines published by the European Organisation for Research and Treatment of Cancer) to the use of the LCP as a framework or set of principles for care of the dying.

For example, Dr Mark Boughey talked about the process of slightly adapting the LCP with language ‘pertinent to the Australian environment’ whilst at the same time trying to ensure congruence with the ten key principles of the LCP promoted by the Liverpool innovators. In Norway, Professor Dagny Faksvåg Haugen recalled using LCP version 12 in a ‘formal translation … based on EORTC principles’; very few changes were made and the document was subsequently used in a range of care contexts. Similarly, in Sweden, Professor Carl Johan Fürst recalled that the process of translation into Swedish was relatively unproblematic. Dr Elisabeth Medicus in Austria described both the use of the LCP documentation already developed and translated in Switzerland, and the process of registration required both with the Liverpool ‘home’ team and the German speaking ‘DACH’ collaborative. However, she reported that the term ‘pathway’ caused an issue in Austria for reasons that were not solely cultural or linguistic, recalling publication of a book called ‘Dying in Peace’ that provided a critical perspective on pathways. The book garnered attention at the time the LCP was introduced in Austria, creating some sensitivities around use of the term.

Interviewees from Argentina, Denmark and the Netherlands described how they engaged in a process of both linguistic translation and cultural adaptation. In Argentina, cultural perceptions about the meaning and temporal associations of ‘dying’ and ‘death’, as well as the lack of significance and meaning of ‘Liverpool’ or ‘pathway’ for Argentinians, led to a completely new term being used. Dr Gustavo De Simone emphasised the pre-eminent importance of the Spanish concept of death as a ‘moment in time’, and the cultural difficulty Spanish speakers might therefore have with the northern European notion that dying is a process. The solution was to use the acronym PAMPA, which stands for ‘Programa Asistencial Multidisciplinario Pallium’ and also brings to mind the Pampas grass of rural Argentina. A secondary descriptor was then added, using the words: ‘integrated care plan for patients in the end of life’.

In Denmark, a cultural difference in the meaning of the term ‘dying’ led to an interesting ‘work around’, which Dr Svend Otteson explained:

So, when we’re talking Liverpool Care Pathway and the last 48 hours we had a problem with the terminology there. So, we used the terminology for a kind of making it easier for us - talking about the dying-dying patient. You have the dying patient [the] imminently dying or the dying patient as a definition. The dying patient has hours or a few days or a few weeks left. But when you’re talking about Liverpool Care Pathway you have probably two days left. So, there was a confusion around terminology using dying patient and the Liverpool Care Pathway using dying patient. So, to stress or to highlight that it was the dying patient in the Liverpool Care Pathway we used the term dying-dying patient. (Dr Svend Otteson, Interview 16)

A similar issue occurred in Japan, where for cultural and linguistic reasons the term ‘Mitori’ –which has broad resonance in Japan and evokes the notion of being with a dying person - was used instead of a literal translation of the word ‘dying’. Translation and adaptation was further complicated in Japan because the flow chart for pain management in the UK version of LCP was considered unsuitable for Japanese practice. Accordingly, in Japan an emphasis was placed on the concept of the LCP, rather than on the detail. The solution was to encourage clinicians working in different areas to develop their own flow chart based on the LCP example:

…I’d say it was difficult to agree with the translation, especially on this algorithm … (about) the usage of medicine. But at that time we did not have clinical guidelines of pain management (and) … they could not agree with this flow chart … After I became principal investigator, we didn’t emphasise the flow chart. (We said) this is simply just an example. (Professor Mitsunori Miyashita, Interview 10)

In the Netherlands and in New Zealand careful attention was paid to the suitability of all the goals of care in the UK version of the LCP, and some revisions were made for cultural reasons. In the Netherlands this became necessary after the changes made in the UK version LCP 12 introduced what was perceived as an unnecessary focus on clinically assisted hydration and nutrition:

We had a second version and that's based on version number 12 in the UK. But the difficulty between version number 11 and version number 12 is that there was already a lot of problems in the UK and therefore there were two new goals about fluid and feeding. It is a discussion we can't understand ... really understand in the Netherlands. … so we didn't make it two different goals … we put it together with our judgement about, for example, oxygen and antibiotics, so it's part of another goal and not one itself. (Dr Lia van Zuylen, Interview 9)

In New Zealand the view was that cultural and spiritual care goals should be separated:

In consultation with the Liverpool team, we had another goal added to the list of goals of care on the LCP and that was around cultural support and cultural care. Here in New Zealand we felt that putting spiritual care and cultural care together wouldn’t be appropriate … There were some language differences ... and that did have an impact on how we taught people to use the documentation. So it did have some limitations around some of the language. But the [Liverpool] team allowed us to modify some language, but not all language and that did cause some confusion at times, I think. (Dr Simon Allan and Bridget Marshall, Interview 2, BM speaking)

In Italy and Belgium, a very different set of circumstances prevailed. The research-based approach taken there to the development, adaptation and trialling of a complex intervention based on the LCP meant that considerable changes and adaptations were made to the original LCP programme, and these went far beyond a strict translation of the core document.

Dr Costantini in Italy recalled that while translation of the paperwork was relatively straightforward, the development of a detailed implementation manual - which he saw both as essential and lacking in the original UK model - was more complex. He describes how such a manual was developed based on the core principle that the implementation of the LCP in Italy must be led by a specialist palliative care team:

We started with the idea of implementing the LCP as part of a research framework, as part of a research project. The first thing we did was the translation of the document, it was required by the international LCP group. They revised our translation and they accepted our adaptations. To be honest it wasn’t completely different from the original. In my opinion a care pathway is not just the document but also the way to implement the document. We didn’t change the document a lot just small adaptations, which I have put in context, but we probably changed a lot [in] the way we implemented the LCP. First we wrote a manual for its implementation. We did all these things before receiving the answer from the Ministry of Health. The manual for implementation was based on the idea that the LCP had to be implemented by a specialised palliative care team. The palliative care team was responsible for the process of implementation and the appropriateness of the procedures, of training about end-of-life of care and correct application of the LCP. So in our approach the specialised palliative care team was a necessary condition for doing that. (Dr Massimo Costantini, Interview 19)

This approach was mirrored in Belgium, where the research team closely collaborated with the Italians, as well as the UK and Dutch teams, in the development of an intervention for use in geriatric wards. As in Italy, the main challenge was seen as the development of a guide for implementation:

So we started by talking to people in the Netherlands and also to Massimo Costantini in Italy, we knew he was doing a big trial in hospital with cancer patients so we went to Italy to talk to him and we went to the Netherlands to talk to those people and we also went to Professor Ellershaw’s group in the U.K. We started with the various documents and the programme and thought, well, what can we use, what is suitable, do we need to adapt this and how can we make this work in Belgium? I think the main changes were in wording, but the main challenge was working out step-by-step the implementation guide. For the document itself, we did not make very many changes. We started from the LCP process (‘Zorgpad voor de Stervenfase-RotterdamZS-r(lcp)’) used in the Netherlands, and we mainly adapted the language and added some extra things for older people and some care goals. (Dr Tinne Smets and Dr Kim Beernaert, 15, part 1, TS speaking)

***Deployment and diffusion of the LCP within countries.*** In all countries except Belgium, deployment of the LCP was initiated by specialists in palliative care. However, there was considerable variation according to where the LCP was first used, and whether this was solely in a specialist palliative care context, a generalist care context, or in both. Similarly, there was variability in the patient population targeted: in some countries (for example the Netherlands) the primary target was oncology, in others (such as Belgium) the target extended into broader categories of patients approaching the end of life, such as older people with end stage frailty or dementia. There was also variation according to whether the use of the LCP remained confined to one or two small local areas, or was diffused regionally or nationally, and the extent to which this was organic and unplanned, or strategic and systematic.

In this section we look at interviewees’ accounts of the care setting they targeted when they first starting using the LCP and the associated patient groups with which they sought to employ the LCP. We also examine their accounts of diffusion within the countries where LCP was deployed. Insights into the factors and mechanisms influencing the different levels and types of diffusion emerge from these accounts and are summarised in Table 3 They include the presence or absence of: some form of nationalised health care system, a national policy for palliative care into which the LCP (or a version thereof) could be inserted, and the extent to which there was some level of integration of palliative care services into mainstream health care; funding for a programme of research on the LCP or its implementation; and a wider quality control or governance structure onto which the LCP could be grafted. 

**Table 3.** Levels and mechanisms of diffusion.

	Local	Regional (limited)	Regional (extensive)	National	Mechanisms
India		x			• Professional networking/ endorsement • Training
New Zealand				x	• Professional networking/ endorsement • Government endorsement • Funding by Government • National coordinating office • Network of facilitators • Training
Argentina		x			• Professional networking/ endorsement • Government endorsement • Training
Germany		x			• Professional networking/ endorsement
Norway				x	• Professional networking/ endorsement • Government endorsement • Funding by Government • National coordinating office • Network of facilitators • Training
Sweden				x	• Professional networking/ endorsement • Government endorsement • Funding • Training
Australia			x		• Professional networking/ endorsement • Government endorsement • Funding by Government • Regional coordination • End of life care champions
Netherlands				x	• Professional networking/ endorsement • Government endorsement • Funding by Government • Training
Japan		x			• Professional networking/ endorsement
Belgium			x		• Professional networking/ endorsement • Government endorsement • Funding by Government / health agency • Training
Denmark	x				• Professional networking
Switzerland			x		• Professional networking/ endorsement • Government endorsement
Austria	x				• Professional networking/ endorsement
Italy			x		• Professional networking/ endorsement • Funding by Government / health agency • Training

In those countries where there was no ‘national’ or centralised mechanism for the uptake of health care interventions, including elements of LCP in professionally endorsed guidelines for palliative care was seen as the most effective way to encourage its use. In these cases, not only were numerous versions of the LCP forthcoming, contrary to the UK originators’ intent that LCP should retain a standardised format, but the ‘spread’ of the LCP was relatively organic in form. Moreover, in these cases, LCP uptake was dependent upon clinicians to make an active choice to ‘opt in’. There was no element of compulsion, as was the case in those places where its use was mandated under a national strategy.

Local diffusion

At one end of the continuum in terms of target of use and level of diffusion were Denmark and Austria, where in each case use of the LCP was narrowly confined to the initiator’s own specialist palliative care unit.

In Denmark, Dr Otteson described local implementation of the LCP, focused first in his own specialist palliative care unit, where it became a standard protocol for the care of the dying between 2009 and 2015, and then in limited use in the general oncology wards of the same hospital. In his account, he provides interesting insights into some of the factors limiting further spread:

We started preparing, introducing the implementation in 2005 or 2006, something like that. But we were a rather new palliative care unit and that was the reason why it took some years before we started the Liverpool Care Pathway. We were in fact the first I think and also the only department or unit in Denmark at the time with the Liverpool Care Pathway … We don’t have the same organisation as you have on a national basis … so if you are a private or local unit, you need to introduce, by example … I had done a lot of teaching at Roskilde hospital and I had of course spoken about the Liverpool Care Pathway all over Denmark. So, many people knew about the Liverpool Care Pathway. We thought about making a national centre for Liverpool Care Pathway or for the care of dying patients, but Britain I think has the culture or time or what you call it to do so … we don’t have an organisation where we just say now we do it on a national wide basis. (Dr Svend Ottesen, Interview 16)

In a similar account from Austria, Dr Elisabeth Medicus described how she used the LCP in her own inpatient specialist palliative care unit, working with some nursing colleagues and as part of the German speaking countries ‘DACH’ collaborative, which was led by Professor Eychmüller who was working in St Gallen, Switzerland:

In 2008 we had a kind of a study group visit in St Gallen … two nurses and me. Then we applied [for] the registration in Liverpool, also in 2008 and … then we started, in 2009, to implement it in our institution, only in the inpatient palliative care, in the inpatient ward and we started with version 10 at that time. (Dr Elisabeth Medicus, Interview 18)

Dr Medicus went on to describe how she tried to collaborate with staff in an Austrian nursing home once she became aware that they were using the LCP for residents with complex long-term conditions, but the link did not develop and she was unaware of the extent of any wider use in Austria. 

Limited regional diffusion

Limited regional diffusion was described by interviewees from India, Argentina and Japan.

India and Argentina were the only low and middle-income countries represented in our study and in each case interviewees described attempts to introduce the LCP into both hospices and general palliative care settings (with an emphasis on oncology patients), but in contexts where these efforts were geographically limited. The introduction of the LCP to India in 2006 was described as an initiative prompted by a UK doctor who had an elective in India, and as a collaboration between the Institute of Palliative Medicine (IPM) in Calicut and the Indian Association of Palliative Care, which endorsed the proposal. Despite these endorsements, lack of funding meant that use of the LCP in India was limited to four discrete areas. 

In Argentina, a dearth of funding and of a wider infrastructure for palliative care similarly circumscribed efforts to ‘spread’ the PAMPA programme beyond its original site in Buenos Aires. This was despite considerable efforts to engage practitioners from all over Argentina and from other countries in South America, through education and training about end of life care and the use of the LCP:

So we have professionals, students, professionals from Argentina, the different provinces and also other countries from South America. And when we teach around this programme they like to implement it in their institutions. They like it very much but don't have the structure, or the political decision or the way to implement these kinds of programmes. Because again, the palliative care … probably they don't have a team, multidisciplinary team or it's just a doctor and a nurse for a whole province. So it's difficult to think at this time to implement this kind of programme …We had at the time the illusion that it would be a more spread programme. But till now we have one more hospital, one more home based care team or programme, and a hospice. The last to join is a hospice, a little hospice. But we have now five institutions involved in the programme. (Dr Vilma Tripodoro, Interview 3)

Dr Tripodoro’s colleague Dr Gustavo de Simone also gave an account of the process of spread in Argentina, describing how it was linked to the education of physicians and referring to a plan to expand the implementation of the LCP to Patagonia, though at the time of the interview this had not yet occurred.

In Japan, limited regional use of the LCP was also reported. One of our two Japanese interviewees, Professor Miyashita, referred to a pilot study carried out following earlier piloting of the LCP in two specialist palliative care units, and which drew attention to a number of problems associated with use of the LCP in general oncology wards. He reported that the ‘explanation and training were insufficient’ - medical staff did not wish to use it, nurses were worried about recognising whether someone was dying and there was a lack of necessary resource to support staff and sustain systematic implementation. The work was abandoned and modifications were considered, but then the LCP was withdrawn in the UK and everything came to a halt.

Professor Miyashita estimated that around 20–30 individuals from ‘maybe four or five hospitals’ had used the LCP at some point in Japan, although it was difficult for him to be sure of the extent of wider use. There was also a circumscribed attempt to develop a home care version of the LCP. This was described by Dr Ai Oishi, a Japanese general practitioner who collaborated with Professor Miyashita. Dr Oishi had spent time in the UK and, on her return to Japan, found information about the Japanese version of the LCP on the internet, prompting contact with Professor Miyashita to request a copy of the document. Dr Oishi’s intention to adopt the LCP in general practice in Japan came to partial fruition, as she gained experience of its use in the course of her homecare training:

Well the challenge was to implement it with nurses and then other professionals [but] without support from other people … I knew I couldn’t implement it. (Dr Ai Oishi, Interview 11)

Professor Miyashita (LCP 10) described how he was asked by a member of the Japanese government about the potential use of the LCP nationally to improve end of life care but had advised against its incorporation into government policy because, in his view ‘it’s immature … You cannot incorporate it in national governmental policy’. (Professor Mitsunori Miyashita, Interview 10).

Extensive regional diffusion

More extensive regional diffusion was reported from Switzerland, Germany, Belgium, and Italy. In Switzerland, extensive regional spread followed the work of Professor Steffen Eychmüller (Interview 17) of the Cantonal Hospital of St Gallen and founder of the DACH collaborative, who recalled his intent from the outset was to use the LCP to improve the care of the dying in general palliative care contexts. The translation of the LCP, and the development of an associated training programme, proceeded with this goal in mind. An opportunity then came to establish a quality improvement initiative in oncology, in which care of the dying became one of the standards:

I used to work in St. Gallen in the beginning and we decided to become like a collaborative centre for the German speaking regions. We translated everything, the whole document and we also established the training programme in German for health professionals. We had also, in early 2003 I think we started, we got the opportunity to establish in the whole hospital a programme for oncology improvement and one of the seven standards became care for the dying. So this was, for us, something like a lap to establish and to test the dissemination programme with a tertiary hospital ... and also to use the quality management circle that has been established in many surroundings in our German speaking world as a vehicle to improve care for the dying … if you combine quality management, quality improvements in hospitals together with such a topic, it works. (Professor Steffen Eychmüller, Interview 17)

The subsequent broader regional diffusion that occurred in Switzerland was dependent on voluntary collaboration or ‘opt in’ by other providers of palliative care. Uptake was encouraged through work led by Eychmüller to include elements of the LCP in national guidelines for palliative care, which clinicians were encouraged to use to develop their own version of the LCP:

In our country, we worked out and developed together with the other language regions [in Switzerland], a national guideline for how to deal with people and their family carers during the time of dying. This was mainly based on the competencies and knowledge we had from the Liverpool Care Pathway but it served more as a framework document so that different institutions, from hospitals to community care to nursing homes, could derive their own versions from this framework version. (Professor Steffen Eychmüller, Interview 17)

In Germany, through the DACH initiative, initial use of the LCP also took place in specialist palliative care units. Professor Raymond Voltz reported that although some spread then occurred in general palliative care settings in Germany (especially in hospitals) its extent was limited, as in Switzerland, by the lack of centralised organisation of the health care system and no precedents for national implementation projects of a similar type. He observed that the recommendations from the Liverpool team for national implementation would ‘never have worked’ and that its use depended on the personal initiative of ‘active’ individuals. As in Switzerland, diffusion was dependent on the inclusion of aspects of the LCP in German national guidelines for palliative care:

Well, at the moment it's implemented in several … as far as I know in individual institutions around very active people. And once they leave the Liverpool Care Pathway is also dying in its use. I would say it's not more than maybe 30 [hospitals] or something like that. So it's not really implemented by institutions I would say. Because we don't have that national level at all as in the UK … It's very fragmented and regional over here, and so it would have never worked …We have national … our national guidelines for palliative care. One of the components is on the care of the dying ... our national guidelines are built around the content of LCP. So actually this is the best as we can get on the national level. (Professor Raymond Voltz, Interview 4)

In Belgium, implementation of the CAREful programme, based on the LCP, was reported to have extended to around 70% of hospital geriatric wards in Flanders, following the completion and reporting of a cluster randomized trial. Implementation in each case was preceded by the requirements of registration and attendance at a two-day training programme. The process was formally supported as an implementation and evaluation programme by the National Cancer Society, as described by our Belgian interviewees:

So we aimed for 30% coverage but now we are already at 70 or 80% coverage. Just hospitals. You see it’s not a funded programme it’s an implementation project funded by the Cancer Society … all 'control’ wards after the intervention was finished also got the training, and could use the implementation. (Dr Tinne Smets and Dr Kim Beernaert, Interview 15)

A similar process took place in Italy, where the LCP was evaluated in a programme of research that culminated in a cluster randomised trial on general medical and respiratory hospital wards and was followed by a time-limited period of broader implementation. This took place in hospitals in a number of regions in Italy. Implementation in hospices in one region, Liguria, also occurred after the formal trial. As we have seen, the research and implementation programme was led by Dr Massimo Costantini from his workplace in Genoa, under the auspices of the palliative care network of the Italian National Cancer Research Institute. Dr Costantini had gained funding from the Italian Ministry of Health and describes here the early days of the research:

When we did phase two we implemented LCP in three medical wards of Genoa and one respiratory disease ward in Genoa, in four wards, and we assessed the impact of the LCP before and after the implementation of the LCP. We published three papers in *Palliative Medicine*. One methodological, one with the results of before and after and one where … we interviewed the professionals, physicians and nurses, before and after the implementation about expectation and the perceived efficacy of implementation, and problems of course. The goal of phase two, as [reported] in these three papers, was to decide if we could start with a randomised trial. In the meantime, we received a positive answer from the Minister of Health and the project was funded. In phase two at the end of implementation we decided to slightly change the programme. We started with phase three but the structure was the same. (Dr Massimo Costantini, Interview 19)

A distinct characteristic of the process of wider implementation in Italy was that Dr Costantini strongly advised against use of the LCP in those circumstances where a specialist palliative care team was not in place in the hospitals requesting use of the intervention:

…our LCP was different from other LCPs. The document was the same but I stressed a lot the way that a care pathway is not just defined by the document you use but also the way you introduce the document because it can make the difference. For example, after the publication of phase two we received requests for LCP documentation from different hospital wards in Italy. The first thing we asked them was ‘Do you have a palliative care team in the hospital?’ If no, our advice was before trying to introduce LCP introduce a palliative care team and then we can discuss about the LCP, that was our vision in Italy. (Dr Massimo Costantini, Interview 19)

National diffusion

Norway, New Zealand, Australia, Sweden and the Netherlands all experienced a degree of national spread of the LCP, with a comprehensive range of patient groups and care settings targeted. 

Interviewees from Norway, New Zealand, and Sweden described how the LCP was used first in hospices and then in a range of other general palliative care settings, such as hospitals and care homes. While the degree of centralisation of their health services varied, with New Zealand having a comparatively centralised system, compared to Norway, Sweden and the Netherlands, in all cases the broad extension of the LCP to general care settings was facilitated by a national body.

In Sweden, the LCP was introduced in 2007 as part of a national project monitored by a palliative care competence centre. Professor Carl Johan Fürst recalled that, even though Sweden (like Switzerland and Germany) was a ‘very decentralised country’, the LCP was used widely, known about on a national scale, and included in Swedish national guidelines about palliative care:

…. there are services all over the country using it in palliative care, but also in some nursing homes or care homes. It is actually used and I think, as far as I know, also in a few hospital wards … not everybody is using it, but everybody knows what it is. It’s also … you can read about it in the national guidelines, it is recommended there, although we have changed the wording a little bit in the later editions … you need to know that the government in Sweden is not very … it’s not a very centralised country, it’s a very de-centralised country. So, the government, they can make some recommendations, but they cannot tell you what to do. (Professor Carl Johan Fürst, Interview 7)

Professor Fürst also explained that in addition to the guidelines, another mechanism of diffusion was via the quality indicators or parameters in the National Palliative Care Registry that were based on the LCP:

We have a national registry for palliative care, which is actually a registry where you register every patient after death … This registry is covering about 70 per cent of all expected deaths in the country. The quality indicators or parameters in the registry are very much taken from LCP. (Professor Carl Johan Fürst, Interview 7)

By these means, in 2014 the competence centre could estimate that the LCP was in use in over 200 units in Sweden, including specialist palliative care, home care, hospital wards and nursing homes.

In New Zealand and Norway, national co-ordinating offices were set up which enabled emulation of processes of implementation that had occurred across general care contexts in the UK. In New Zealand, introduction of the LCP into a hospice in Palmerston North following participation in a meeting of an international interest group for the LCP in 2008 and sparked a process of national diffusion, marked by the establishment of a national LCP office in 2011:

…we managed to persuade the Ministry of Health that this was a good and useful, positive assist for good dying, if you like, in all settings and I suppose the question of quality … rang a good sound with them because there was some Ministry support for leadership and palliative care at the time. We argued strongly from our hospice that we should set up a national coordinating office as the best means of getting a unified and bench-markable process across all of New Zealand. For four years or so we managed to succeed in that process … (Dr Simon Allan and Bridget Marshall, Interview 2, SA speaking)

New Zealand had an extensive existing infrastructure for palliative care and employed nurse practitioners sourced from district nursing services or specialist palliative care services to liaise with GPs in the community to enable the use of the LCP in residential care settings and the domestic home. They called this the ‘palliative care partnership’:

… and it was through that mechanism of partnership and leadership from general practice that we were able to get a good uptake by general practitioners working with our specialist nurses from hospice and the district nursing service to apply a lot of pathways to home death as well. (Dr Simon Allan and Bridget Marshall, Interview 2, New Zealand, SA speaking)

A similar level of infrastructure existed in Norway, where the LCP had been initially introduced in the early 2000s by a leading palliative care physician in the first palliative care unit in Norway. The LCP was then translated for wider use by our interviewee’s palliative care centre in Bergen and first used in 2007 in Bergen hospital and in a nursing home, before being taken to the larger University hospital in Bergen. A process of nationwide spread then started, driven mainly by requests from other institutions for help. This involved the Bergen municipal area initially, and then other regions.

So first we started in the first hospital, then we took it to the main University hospital in Bergen, and we presented it to the management and they were very positive. And we decided on some wards where we wanted to introduce it first, and try it out. And we applied for money from the Norwegian Medical Association and from the health authorities. And they were all very supportive, and we started a project. And then we had all these requests from the rest of the country. ‘We have heard of the LCP somewhere and we want to start using it, can you help us? We've heard that you have a translation’. So it just added on. And we never really promoted it. We never really went out to advocate it; it spread by itself. (Professor Dagny Faksvåg Haugen, Interview 5)

Professor Faksvåg Haugen described the diffusion of the LCP in Norway as national, although she pointed out that, as in some of other countries, there was no compulsion for its use at a national level, preferring the term ‘a national spread’, resulting from endorsement by the government as an option for good care of the dying, captured within the National Action Programme for Palliative Care in Cancer. This supported a loose implementation infrastructure around the LCP built on an existing palliative care system and bolstered by grants gained from a variety of sources:

We have networks of palliative care nurses and cancer nurses in most parts of the country. So we have these resources in every nursing home, in every home care district. So we had sort of a good basis for the spread. There were people who could be ambassadors and advocates and also who could do the training and education. We had in really many places a very good structure to use. And then there have been a lot of funding opportunities. I already told you that we had funding from different sources, the Medical Association, the healthcare authorities, the Directorate of Health and the hospital. Many projects all over the country received money from the state for implementation. And we did a survey and we found that about 20 per cent of all users had had specific funding to implement the LCP… the funding also gave us the possibility for a position for a network coordinator who worked on a national level. (Professor Dagny Faksvåg Haugen, Interview 5)

In Australia, a scenario occurred that was very similar to New Zealand and Norway. The LCP was widely used, especially in the states of Victoria and Queensland, with some aspects of it disseminated nationally in residential aged care. In Melbourne, Victoria, Dr Mark Boughey recalled that soon after he took up his post at St Vincent’s Hospital he had contact with a nursing colleague who shared his interest in finding ways to improve care of the dying outside of specialist palliative care contexts. The nurse had set up a special interest group with representatives from the state of Victoria. The group considered the LCP as a key means to achieve its goals and worked over a three-year period to develop an Australian version of the LCP that was ‘congruent’ with the principles of the UK intervention. This quickly drew the attention of state health policy makers in Victoria, where a quality improvement initiative focused on acute care settings was under development:

That’s when the LCP really came to the table … about 2009 … and it really got the attention of our policymakers in our state government, who saw this as a very key part of Victoria being ahead of the game. (Dr Mark Boughey, Interview 12)

Dr Boughey goes on to describe the LCP project in Victoria as a ‘clinician-driven initiative supported by the government’ that quickly spread throughout generalist services, with a sole emphasis initially on acute care, including the stroke clinical networks (Interview 12). Boughey perceived that the LCP project in Victoria had a synergy with the wider focus of a state-based quality improvement programme in acute care. This ultimately resulted in the introduction in hospitals of a series of quality measures for end of life care:

In 2016 it was actually signed off by the health minister that the acute health service had to demonstrate how they were implementing care plans for the dying into their health services. It was a measure that was directly reported by the CEO of the hospital to the health minister. They had a series of quality measures that they had to report against in end of life care. (Dr Mark Boughey, Interview 12)

Dr Boughey also considered that the project in Victoria both shaped and reflected similar work in acute care contexts in other Australian states, although he reflected that Australia never achieved a full national approach or mandate for the use of the LCP. This was recognised in the Australian National Strategy for Palliative Care 2010, where a call was made for an integrated approach to end of life care across all care sectors. Later in his interview, Boughey reported that a ‘pared down’ version of the LCP, developed without reference to the Liverpool team of original innovators, was widely introduced across Australia in residential aged care. This was supported by funding from the Commonwealth Government in Australia, which is responsible for aged care. In a similar account, our other Australian interviewee, Associate Professor Carol Douglas, reported how in Queensland the LCP was used across general hospital settings in 17 service districts:

Queensland is a very large place and it was decentralised to 17 Health and Hospital Services. In consultation with Queensland Health, and given that we had not had any reports of problems, you know, I mean significant problems, in relation to the use of it, they sanctioned the continuation of what was then the Care of the Dying Pathway given that each hospital committed to appropriate governance, education, et cetera. So, it rolled on. (Associate Professor Carol Douglas, Interview 8)

In the Netherlands, as we have seen, Dr Lia van Zuylen was first introduced to the LCP in the early 2000s and was encouraged to take a research-based approach to its adaptation and use. This started with a pilot study in the Erasmus MC, a nursing home and a hospice and was followed by a larger scale study in eight institutions. By the time of her second interview with us in November 2018, Dr van Zuylen reported that since 2009, the Dutch Comprehensive Cancer Centre Netherlands (CCCN) had become the national implementation ‘machinery’ of the LCP via 67 regional networks, using training and telephone links to support interested clinical teams. She also described how the LCP was an opportunity to test out whether an intervention could be implemented on a national basis as the structures of the CCCN gradually evolved.

This adoption of the LCP by the Netherlands Cancer Institute resulted in national spread, but the voluntary engagement of clinical teams (and some uptake outside of the structures of the Institute) meant that it was not possible for Dr van Zuylen to be entirely sure about the its extent:

We are trying to get some feeling about it and I think that it is around 200 organisations using it now, but I can't give you the exact figures … We are working with it and new organisations are starting it and mostly they have contact with the Comprehensive Cancer Centre, but not all of them. (Dr Lia van Zuylen, Interview 14)

***Perceived benefits of the LCP***

A systematic approach

We have seen that most respondents anticipated that the introduction of the LCP would lead to a more systematic approach to end of life care. This was an aspect of the LCP that went on to be highly valued once it was introduced into practice. For example, in Austria, Dr Elisabeth Medicus described positive impacts in a specialist palliative care unit on the process of decision-making, especially in relation to the diagnosis of dying and of symptom control, and on communication with patients’ relatives:

I think that, in our team, which was composed of really committed people, everyone liked that it gave us a, kind of, security and it was also … so that clear decisions, it brought us clear decisions about ‘this patient is dying or not’. The symptom control was better, I would say, and we didn’t overlook anything of importance. Also, I would say that there was advance care planning for frequent symptoms in the dying process. This was especially helpful for nurses and also for us as doctors, because then the nurses didn’t need to call us to withdraw an oral medication or something like this. So, for everybody it was easier and, sort of, valuable. (Dr Elisabeth Medicus, Interview 18)

Some interviewees described how they came to realise that the LCP also provided a systematic framework for teaching students about end of life care, even where there were limited opportunities for its wider implementation as a clinical practice ‘tool’. Thus, again in Austria, Dr Medicus recalled how useful for her teaching she had found the ten principles of the LCP and then later, the recommendations of the Neuberger review, providing a ‘very compact message for many professionals’ (Dr Elisabeth Medicus, Interview 18).

In Argentina, implementation and diffusion of the LCP was limited but education was a key part of wider efforts to build palliative care in the whole of South America, this element was particularly important, as Dr Simone explained:

… on courses we've done specific sessions on the LCP, as a way of teaching about end of life care. It doesn't mean that all the students will implement it, because they need to have the systematic approach, all the phases, et cetera. But they learn how to deal with end of life care, through the LCP, or the PAMPA. (Dr Gustavo de Simone, Interview 6)

In contrasting circumstances, in those countries where the LCP was implemented on a larger scale, the process often provided opportunities for targeted education in ethically challenging areas of end of life care, such as clinically assisted hydration and nutrition. This was the case in New Zealand:

When we were implementing the LCP we were using that as a time for educating clinicians on the importance of communication around hydration and nutrition. So it was a wonderful tool and it did give people a wonderful opportunity, [to] give increasing knowledge about the benefits and burdens of artificial hydration or nutrition and to look at the ethical issues around that and then to look at the real importance and need for communication around that. (Dr Simon Allan and Bridget Marshall, Interview 2, BM speaking)

Interdisciplinary communication and positive impacts on nursing work

Many respondents described an unexpected benefit from using the LCP, in the form of a positive impact on communication and interdisciplinary team working. The value of LCP in helping nurses to work more effectively and on an equal footing with medical staff was also emphasised by some. For example, Dr van Zuylen in the Netherlands perceived that LCP gave nurses confidence (because of its structure) and a new language to speak about transitions to end of life care with medical colleagues:

I think that gives them the possibility to ask the doctor, ‘don’t we have to start the dying pathway?’ because I think when they say to the doctor, ‘isn’t this patient dying?’, that it was more difficult for them to say than to ask ‘don’t we have to change our care?’ (Dr Lia van Zuylen, Interview 14)

In Denmark, Dr Ottesen described how association of the LCP with nurses’ work changed the power balance in terms of who directed patient care between nursing and medical staff:

The structure of the Liverpool Care Pathway [meant] they didn’t forget anything. So, they were very keen to use it. The doctors were not very happy because their wish for using Liverpool Care Pathway when the patient was dying was coming from the nurses who told the doctors that they had to use Liverpool Care Pathway now. Even so I had to teach the doctors throughout the whole period for the Liverpool Care Pathway, the new doctors and the old ones, but it was still the nurses who went in front and went, ‘now we have to use the Liverpool Care Pathway’. (Dr Svend Ottesen, Interview 16).

Dr Elisabeth Medicus in Austria found that the nurses on the palliative care unit in which she worked were the key to the implementation process: ‘… we did it by the engagement of the nurses’. Similarly, in Switzerland, Professor Eychmüller described how the LCP generated enthusiasm and competence in the care of the dying among nurses, one aspect of which was new procedures to ensure the prescription of ‘as required’ (PRN) medications. This drove the implementation process forward, in spite of a lack of enthusiasm from other stakeholders:

… nurses have been very welcoming and I think this is an international [experience]. They loved it. They felt very much prepared. They supported it very well also in terms of personal training. It was, in the end, really driven by the nursing competency. I think many of the evaluations we did later on also within the quality management brought up that the competency level of nurses was far higher compared to the ones of the physicians … The LCP established the framework and also established the rules that doctors have been obliged to prescribe many drugs for PRN medication for the last days of life. This was what they usually missed. [Before] it was this endless discussion about ‘please prescribe a little bit of this and this drug’ - and this really changed a lot. (Professor Steffen Eychmüller, Interview 17).

***Challenges and drawbacks associated with the LCP.*** Here we report on the most notable challenges discussed by interviewees. In addition, some practical issues were also highlighted mainly to do with the difficulty of adapting the paper-based LCP so it could be used in electronic records, or in the day to day management of the documents in clinical settings where staff were unfamiliar with the LCP.

Scale of education, training and workforce requirements

Interviewees perceived that aspects of the wider societal and clinical understanding of palliative care constrained the extent to which it was possible to introduce the LCP. This was difficult in all of the countries, but especially so in resource poor settings. For example, in India, Dr Stanley Macaden emphasised that neither patients nor clinicians had a clear grasp of palliative care principles, thus making the introduction of LCP very difficult:

… the main thing is palliative care is not well understood by our own colleagues …(and) …a lot of times patients don't know what palliative care is, they think it’s another way of some cure, so they’re willing to grab at any straw. (Dr Stanley Macaden, Interview 1)

At the other end of the continuum, most of the resource rich countries had seen a high profile given to palliative care across policy, practice and public spheres. This created fertile terrain into which to introduce the LCP. As Professor Dagny Faksvåg Haugen, from Norway put it: ‘… palliative care has had a high focus in Norway for many years and we have done a lot to improve skills and knowledge and influence attitudes’. These wider understandings influenced in turn the extent of the training and education challenge perceived to be associated with introducing the LCP. However, whether respondents came from resource poor or resource rich countries, they regarded this as both the most important determinant of scale or level of implementation and the most difficult aspect to sustain, with hospital settings identified as the most challenging environment. For example, in New Zealand, lack of confidence and training among hospital clinicians in communication skills related to end of life care were described as a ‘core challenge’ to the whole project of introducing the LCP:

When it came to hospital settings, the challenge there was...and I’m now looking retrospectively to some extent, it really was hitting to the core challenge of communication around death and dying and the lack of desire by clinicians to go there, the lack of confidence and training in that area and really reflecting the poor way in which death and dying was done and to some extent still is done in a hospital setting. (Dr Simon Allan and Bridget Marshall, Interview 2, New Zealand, SA speaking)

Turnover of staff was identified as a problem in many settings, shown here in comments from Argentina and India:

The challenge, first of all, is the way to train the team, the different teams. And when the training is done I think the challenge is to train more people the next year, or the next time when people change and new doctors come or a new nurse comes, and we have to start again. (Dr Vilma Tripodoro, Interview 3)

This is where the problem is because junior staff and nursing staff also, doctors and nurses at the junior or the middle level they keep changing and they keep going. And unless you are very knowledgeable about that and aware that this will happen, just because you’ve trained, done a fantastic training for one set of people you have to do the same thing again. Once you do that it’s a regular thing then you can get results. Training is key in this. (Dr Stanley Macaden, Interview 1)

Where funding was forthcoming from governmental sources, it was possible to ameliorate this challenge by creating facilitator networks, establishing dedicated funded posts for nurse practitioners or nurse consultants, or using the model of practice ‘champions’. As we have seen, it was sometimes also possible to capitalise on quality improvement programmes already in place that had a much broader focus than end of life care. Australia was a case in point:

One of the things that we did over time, was that I did identify a champion for Care of the Dying [in each ward]. We would pull together those individuals from the different wards once a month and provide in-depth education. Then, they would go back to provide that education to their nurses, because it’s just not possible to provide education to every nurse on the wards. That works very well. (Associate Professor Carol Douglas, Interview 8)

[The] acute health environment already had a very strong national quality framework built in, even though it didn’t have specific criteria for end of life care or dying. We were quite familiar with having national quality cycles, a four-yearly cycle of quality improvement and introduction of documents and care plans, pathways needing ongoing review. So, for the rollout and education and the orientation and adoption of these things … systems were already in place. (Dr Mark Boughey, Interview 12)

Involving senior medical staff in education and training initiatives was found to be difficult by all interviewees, even when funding was available for the purpose. An example of this was manifest in the large scale research-based implementation and evaluation of a version of the LCP in Belgium:

I think the main challenge was getting the physicians involved and especially in the training …The PhD student that was working on the trial had sleepless nights over it - she had had enough of it after four years … That’s always the hardest part, to find physicians. Nurses and other staff, they are mostly motivated, but to find the physicians to take two days’ time to come to a training, it’s often more difficult (Dr Tinne Smets and Dr Kim Beernaert, Interview 15 part 1, KB speaking)

Tensions between standardisation and variation

Some interviewees reported the tension that arose between the need they recognised to alter the LCP (so that it made sense in their context and culture or in the light of their experience) and a desire for standardisation. The latter came partly from their concern to align themselves with international colleagues by use of a standard LCP ‘tool’ and partly from the concern of the Liverpool ‘home’ team to maintain ‘quality control’ over the translation and adaptation process. Professor Raymond Voltz captured this tension in his recollection of the development and use of the LCP in the German speaking countries:

It’s very formalised and it used to be very UK dominated and driven. And so initially it was not possible to change any single word. I would say this is a real hindrance to using it as an instrument. It could not be locally adapted. You had to get registered in a very strict form. And so following all these steps. And sometimes I had the feeling that the emphasis of the group was more on the formalised technical aspects than on the content, and improving content. And that was personally for myself, but also for many people I know, it still is very counterintuitive … If this was used in an open way, just everybody could use it, and then we could collect and grow and learn from each other. It was not meant to learn from each other, the experience, this was just meant to get distributed 100 per cent as it is. (Professor Raymond Voltz, Interview 4)

The withdrawal of the LCP in the UK and the associated freedom from Liverpool copyright requirements led to quite considerable adaptations of the original documentation. In some cases, it was clear that variations of the LCP pre-dated the UK withdrawal. For example, in Victoria, Australia, Dr Boughey recalled that as the implementation process unfolded, extensive use of the core LCP idea was employed locally by service providers to develop a variety of end of life care plans (especially in residential aged care) that were relevant and useful:

… the LCP was pinched and rebadged and reimaged a little bit by a lot of services for their own usage. A couple of the tools that were developed were really the LCP, but, you know, pared down or a modified form. (Dr Mark Boughey, Interview 12)

Similar situations occurred in New Zealand and in Switzerland. In New Zealand, implementation spread from one locality through clinical networks to several areas. Our respondents described how each area made ‘their own mark on it… we had various documents that resembled LCP but I would think it would be fair to say there were at least a dozen in operation in New Zealand’ (Dr Simon Allan and Bridget Marshall, Interview 2, SA speaking). In Switzerland, the development of regional and local versions of the LCP was encouraged, provided these addressed the key principles and competences of a palliative care guideline produced by the DACH collaborative and inspired by the LCP. In this way, national spread was encouraged in spite of a regional government structure:

The adaptation to local factors and local behaviours and local guidelines they may already have in place, this needs to be offered. So if we [were to] come in with a national standardised document and [say] everybody needs to do it, this does not correspond with our Swiss idea of building up competencies. It's not a national health systems approach. It's a very much a local regional approach (Professor Steffen Eychmüller, Interview 17)

Misgivings about lack of an evidence base and understandings of optimal implementation process

The enthusiasm to adopt and adapt the LCP in many different settings was also matched by a concern, identified by our interviewees, about the strength of the evidence base for LCP and its wider roll out.

In Australia, Dr Mark Boughey recalled that while the rapid spread of the LCP throughout Victoria and other States was associated with the expression of some misgivings about a lack of research evidence, these were quickly overwhelmed as ‘… in practical terms, it was filling a gap that people recognised, demonstrating good care at the end of life’. Other respondents, such as those from New Zealand, acknowledged that if there was a lack of clinical evidence for the LCP from their own countries, they were reassured by awareness of research taking place elsewhere:

We had done some research around the implementation of the LCP in New Zealand initially. However, that wasn’t actually an issue in New Zealand in terms of robust evidence. I think because it was new and the evidence was emerging and there were still studies, and there was the Italian study as well that was going on. So we knew there were studies, yes, it wasn’t a big issue, there wasn’t an issue raised here. (Dr Simon Allan and Bridget Marshall, Interview 2, SA speaking)

Professor Dagny Faksvåg Haugen, described her perception of a critique that had emerged in Norway, concerning the use of LCP in nursing homes for people with dementia, focusing on the debate or conflict as she understood it about whether one category of dying people is similar to another, and describing an attempt to build a consensus position around this issue:

… but we've had some challenges from … well, what should I say, especially one physician and a small group of physicians later on. And that has really been our very main challenge. That said, they think that the LCP is not suited for persons with dementia. They are not opposed to the LCP and support its use in care homes for patients. But say that the plan has not been sufficiently validated in persons with dementia. And the leader is a former nursing home physician but now she is a researcher and head of a research centre for nursing home medicine …. But, then we had taken this, well, I don't know whether I should call it conflict, to the Directorate of Health. And we had a national meeting with this other group and with us, and we had a lot of discussion and the conclusion was there is really no reason to warn against the use of the LCP in persons with dementia. Because a dying patient is a dying patient. All dying patients need care. And we think that in the dying patient individual differences are really much greater than differences based on diagnosis. (Professor Dagny Faksvåg Haugen. Interview 5)

As we have seen, respondents from Belgium, the Netherlands and Italy, adopted a primarily research-based rationale as the motivation to develop the LCP for use in their own countries, recognising from the start that research evidence was a necessary precondition for use of the LCP. However, in each case, they came to the realisation from their studies that the processes of staff training in palliative care and in implementation of the LCP were just as important as any other types of data they might gather:

… you should really make them follow a training programme and I think the main issue in the UK was people can just use it without any training or implementation process to follow whereas here [in Belgium] people have to register to get all the materials and we educate them how to implement it. (Dr Tinne Smets and Dr Kim Beernaert, Interview 15, KB speaking)

I was always, I was very keen on saying [in the Netherlands] please be careful. It’s not about using a care pathway … it is about caring for people who are dying. And it is not about that you have to tick … ticking (the) box has to be done, (but) you have to know what are you doing. (Dr Lia van Zuylen, Interview 14)

Dr Costantini from Italy described how he was aware of the problem with regard to lack of guidance and knowledge about implementation from the start of his work with the LCP:

I didn’t understand very well the way the LCP was implemented in the UK … So the risk of the cooking manual, can you understand what I mean with ‘cooking manual’? The risk of the cooking manual is that ‘it’s very easy, it’s not a problem’ - just reach for the drugs, and so on. The risk was very high … I always asked for the manual for implementation but I realised that the UK group, the Liverpool group, didn’t have a structured manual for LCP implementation. That was in my opinion one of the big problems of the LCP, not just the documents but the way you implemented them. (Dr Massimo Costantini, Interview 19)

Dr Costantini recalled his realisation that the process of training the specialist palliative care team (he describes how he expected them to ‘drive the car’ of the process) in order to lead the implementation in the general wards was going to be something both lengthy and complex. It was this realisation (together with the additional implications highlighted by the UK wide withdrawal of LCP) that was a key determinant in the subsequent recommendation to withdraw the LCP from general use in Italy. Dr Costantini drew a stark contrast between the Italian insistence on the close involvement of the specialist palliative care team and the lack of emphasis that he perceived had been placed either on the detail of the implementation process or on the relationship between specialist palliative care team and general care context in the UK:

When we contacted a centre for the implementation of the LCP we had two kinds of contact. The first one with a palliative care team that we expected to ‘drive the car’ for implementation and the second contact for the hospital team for implementation, so two different subjects. We gave the palliative care team the document for implementation of the LCP of course but also, I can’t remember the number of hours, three fulltime days of training with slides about training a team to be familiar with implementation. We gave them the document for collecting information about the structure of the hospital team, a document for collecting information about palliative care professionals approaching the team and also the most important, the guide for implementing the LCP. This manual is the manual that we gave to the palliative care team and it required well-structured rephrasing, with training without LCP implementation. At the end of the training introducing LCP into the hospital ward, sort of intensive support to the hospital team so it includes for example revising together all patients’ data received into the ward. Then the second part of sub-intensive support and the last part of consolidation. The process of implementation of the LCP lasts six months. So in my opinion it’s completely different from the LCP as it was introduced into UK hospital wards for example, that’s the main difference. (Dr Massimo Costantini, Interview 19)

Professor Raymond Voltz from Germany expressed a similar view at some length, commenting that what was required was enquiry in the health services research paradigm, into the implementation process:

I always take the LCP story in the UK as a perfect example for the problem of getting the second translation. So the ‘second translation’ [is] from clinical studies into [the] real world. And so even if you have done good clinical studies, like Massimo was, of course trying with his randomised control trial in Italy, and he tried to get some more clinical study data. And that was of course…[but] even if in the UK you had done this you could have never… it would have never… and even if the primary endpoint would have been positive because you would've studied it in a warm, academic, palliative care environment, this is different from health service research going out into the field, into everywhere and rolling it out nationally. I think this has not been done … I think the problems around LCP were how it was perceived, how it was not implemented well everywhere, around this delicate and existential problem of caring for the dying. So it tells us a lot that we need health service research. Clinical study data would have helped. But it would not have prevented the LCP disaster in the UK. (Professor Raymond Voltz, Interview).

Dr Vilma Tripodoro from Argentina drew a parallel between what was required in implementing the LCP, and implementation of other aspects of clinical care such as the use of nutrition or antibiotics:

Of course, in general in medicine, well implemented, the use of antibiotics, et cetera. Well implemented, the use of nutrition, et cetera. So of course [when] badly implemented [this] is not a good tool or treatment or whatever. (Dr Vilma Tripodore, Interview 3)

Dr Tripodoro’s colleague from Argentina, Dr Gustavo de Simone, agreed with this standpoint, indicating that it was unrealistic to expect clinical practice in palliative care to be underpinned by narrowly research-based evidence, expressing the view that consensus, expert opinion and clinical experience were equally as important:

… we work in clinical practice, and we assume that most of our practice are not so strong in evidence base. And it's a mix of evidence based, and consensus, and expert, and experience. And we still consider the LCP to be an important document … But of course, it's not perfect, at all. It's true that we should improve our knowledge and approach in a topic that is not so easy to perform research, you know. (Dr Gustavo de Simone, Interview 6).

Professor Carl Johan Fürst from Sweden explained that trying to gather robust and comprehensive clinical evidence for a complex intervention like the LCP was both disproportionate and showed a lack of understanding of the context of practice:

But, to create evidence for a very complex intervention, so that the evidence is robust for positive effect of the whole thing … it’s so difficult and so resource consuming, that … I mean, it’s almost impossible. To demand that is, I think, a bit out of context. (Professor Carl Johan Fürst, Interview 7)

***Perspectives on the withdrawal of LCP in the UK and its consequences.*** Our interviewees were asked how they had felt when they heard about the withdrawal of the LCP in the UK following the recommendations of the National Independent Review, and what their reflections were on the implications for the use of the LCP in their own country. Many interviewees were deeply troubled and angered about the turn of events in the UK, for two main reasons. First, some felt that the conclusions of the independent review were irrational because they covered territory that was much broader than the scope of the LCP on the last days of life. Second, others took the view that Professor Ellershaw had been badly let down by his own colleagues, who sought to take advantage of the ballooning critique in the UK to further their own research portfolios. Professor Voltz in Germany and Professor Fürst in Sweden expressed particularly strong views:

Absolutely shocked and not understanding what was going on in the crazy UK. Because of course we thought ‘oh wow, this is a great thing, that a palliative care tool is now used nationally. Great. And this is an advancement of palliative care using it nationally, thank God that the UK has such a national healthcare system that they can do these things. We would love to have the same thing’. This was the initial approach. And when we heard, we were shocked. We were just shocked. Did of course not understand it at all what happened there. And poor John (Ellershaw) and his group, I mean they were really devastated and we felt very, very, very sorry for them. And it was just very unfair what had happened to them. And it was very unfair how it was treated, even from other groups within palliative care … they used it for argumentation of their own research projects, which was not fair at all. So [we] felt very, very sorry for the Liverpool group and this was absolutely unfair. (Professor Raymond Voltz, Interview 4)

What the hell is going on, what are they doing, what is this? I didn’t understand, at all. I understood, very well, the debate, the media and all that stuff. But, for me, it was like a blame game, where you had to blame somebody and that blame was on the LCP. I think that was very, very bad. I don’t understand how you can blame something that is made to promote good care of the dying and, if it is misused, you blame it. I mean, if morphine is misused, you don’t blame morphine, you blame the clinician or something like that. If an operation is done too much, it’s not the problem with the operation, it’s a problem with the surgeon. This is the same, I think. So, I was very upset and I didn’t understand how this was going on, how it was … that it was sort of accepted by the medical community and even, you know, driven by parts of the medical community. I don’t understand it. (Professor Carl Johan Fürst, Interview 7)

After expressing their initial reactions, interviewees became more reflective, seeking to communicate their thoughts on the underlying reasons for the problems in the UK and the actions they had taken to avoid these in their own countries. Many of these lessons had already become clear in their own activities over the years in working with the LCP and have already been described here; they concerned the realisation that training, implementation processes and strong governance were essential to a successful process and outcome. In some cases, our interviewees came to the realisation that a close and sustained relationship had to be maintained between specialists and generalists in palliative care. All these prerequisites required funding and sustained co-operation and collaboration. For example, in New Zealand, interviewees reflected that the ‘storm’ in the UK was surprising but not entirely unexpected. They reflected that as well as exposing the power of the tabloid press in the UK, it revealed a key weakness in the UK version of LCP, that it apparently prioritised ‘paper rather than principle’:

Well, to some extent, unbelievable and yet believable, you know, tabloid press believable. The storm, however, was a bit unbelievable. My reflection is that it exposed the lack of national and regional coordination of this very important tool, i.e. promotion of the paper but not the principles which I think we picked up on very well. (Dr Simon Allan and Bridget Marshall, Interview 2, SA speaking)

A similar emphasis on principle not paper was manifest in the accounts of other interviewees, as a key reason why they had managed to avoid the problems experienced in the UK. As we have already seen in the DACH collaborative countries, this emphasis, which was in any case necessary because of the lack of a centralised health care system, meant that the LCP was used as a framework of national guidance on palliative and end of life care, but was then subject to local interpretation on the ground. Professor Steffen Eychmüller reflected on the differences thus:

Actually, we had many discussions in our German speaking collaboration, with the Germans and the Austrian people. For us it was not very clear why there was this media led, we would say, hysteric reaction. You also can see that still in Germany and also in some parts of Switzerland people use the term Liverpool Care Pathway without any hesitations because it was well established and people thought why change the winning horse? I think the difference, when we looked to your country, we thought that possibly this idea of standardisation and following the rules is very strong in your country and this might really have side effects if you really follow the rules and the guidelines and possibly, at least in our country, we like rules and guidelines but we like also to adapt it individually. So there was not this fear that by giving guidance you would possibly exaggerate and put someone on the pathway to hell. I think we thought it's very black and white in your country. It's very much this idea once there is a standard approach and once there is a guideline, you need to follow it. Possibly this might be part of the problem. But it's very difficult for us to judge how strict you follow standards in your country. This is something that is difficult to know. (Professor Steffen Eychmüller, Interview 17)

Both Professor Eychmüller in Switzerand and Professor Voltz in Germany recalled robust discussions and disagreements with Professor Ellershaw about the apparent prioritisation in the UK of the ‘document’ over its underlying principles. For example, Professsor Eychmüller recalled ‘many fights’ over the extent to which the document should be taken literally, recalling that once the UK withdrawal occurred, this led to some welcome freedom of interpretation and better enabled adoption of the broad framework provided by the LCP as a basis for national policy in palliative care:

Actually I had many discussions with John and others in earlier times [about] how literally we should take the document. I had many fights like this because I thought it cannot be that we need to make the crosses every four hours and then we guarantee the best care. So I think it's really very much about how strict the document is taken as a guarantee for good quality. … So I think we had a huge discussion about how strict to follow the document and this came also together in terms of how strict translation of a document should be or could be on an international level. Because obviously your document from England represented the style and the attitude and the approach, how you work in your healthcare system. But if we translated it literally in our language, it was very unusual for us. So I think to discuss it on an international level actually opened the door for becoming a bit more, I would say, relaxed and to put it in a place where it is really helpful, so as a very good framework and as a very important area, that you really highlight how important this phase of life is but then leave it to the people to make the best out of it for their use. This is what we actually [do] in Switzerland now. (Professor Steffen Eychmüller, Interview 17)

In Queensland, Australia, Associate Professor Carol Douglas described her view of the events in the UK as a predictable ‘train wreck’ relating the unfolding disaster to a lack of emphasis on governance or overarching control of the implementation process, as well as to the key error widely reported at the time of incentivising the use of the LCP in NHS trusts in England:

I just thought … I mean, in a way, I could almost see it was like a train wreck, because LCP was just growing all the time and everyone was taking it up. But, it just seemed like there wasn’t an overarching control of the process. I think the fact that … there were Trusts that were paying per person that went on the pathway, was appalling. I mean I think that was the undoing … It’s a bit like our HHSs, the Trusts (in the UK) are a law unto themselves … But that it actually had to be completely withdrawn … I mean it just flies in the face of anything we’d ever heard of here in Australia. (Associate Professor Carol Douglas, Interview 8, Queensland)

Dr Mark Boughey in Victoria used the term ‘firestorm’ to describe what happened in the UK before going on to reflect that a key reason the same events did not occur in Australia was the presence of quality improvement structures and associated resources, including educational resources.

In almost all cases, the withdrawal of the LCP in the UK was considered to have had negative consequences for the mission of improving end of life care in the interviewees’ own countries. For example, in New Zealand it coincided and probably was a causative factor in cessation of funding for the national coordinating office for the LCP programme. Argentina was an exception insofar as there was little negative ‘fall out’; instead the Neuberger report was used as an opportunity to try to understand what had happened in the UK. They placed emphasis on the importance of implementation, perceiving that this was missing from the Neuberger report:

… it was mainly with us, in terms of discussing. I remember we had a meeting, ourselves, to reconsider, and of course, to read the document, because, not only the important impact, but also, what we are doing, in that sense. We knew about it, but that didn't change our minds. (Dr Gustavo de Simone, Interview 6)

So I was a bit angry about this report because the problem is implementation … The cost I think for the Liverpool team, also for the population, the cost of this situation with the media and this kind of discussion in the media, I think is very bad for people. (Dr Vilma Tripodoro, Interview 3)

A number of interviewees reflected on the ‘missing’ components of the LCP from their own experience of its use. A key aspect of this was the availability of specialist palliative care advice and help. Dr Costantini in Italy, following his work on the first randomised controlled trial of the LCP, described how a trajectory of research to try and understand and explain the interaction between the specialist palliative care team and the implementation of the LCP was stopped by the international fallout from the UK withdrawal of the LCP, which occurred as the first key publications were emerging from the RCT in Italy:

Well it’s a sad story because it was influenced by the scandal in the UK, the LCP affair. The decision to stop the LCP from your Minister of Health happened during the submission to *The Lancet*. *The Lancet* was rather severe in our conclusion of the results because *The Lancet* study, the phase three, was presented and left just at that and it is formally a negative trial because the P value is above 0.05, you know what it means of course. But in my opinion in the outcomes we have said we could observe a positive trend, a positive direction. I interpreted a negative trial just for the P but it was the result of six months’ hard work of the palliative care team in a ward and the results are not so big as we expected, it’s a little improvement probably but not so big. So it’s negative for the P-value greater than 0.05, and the observed improvement was not so big to justify the costs of the implementation of the LCP, in my opinion because there is an improvement but not so big. It’s not justified by the hard work of the palliative care team … It’s a pity because in my opinion it was a line of research that could go on but unfortunately what happened in the UK stopped any reflection, any possibility to go on in this line of research. This is an important point for me … What happened in the UK stopped any kind of research on the LCP and that was a problem for everybody. I would like to study which is the active component and the component not active of the LCP because there is something good and something bad in the LCP. Now it’s very difficult to do that. (Dr Massimo Costantini, Interview 19)

Others also came to a similar conclusion about the critical role of the specialist palliative care team, albeit from a different form of engagement with the LCP. In Denmark, Dr Otteson reflected on his experience of the work required to provide education for implementation and expressed a lack of surprise that the LCP ran into problems in the UK. He emphasised that it is essential to have specialist palliative care resource available immediately to help non-specialists:

I think education and teaching is of course necessary for implementation. But I think our experience is that there has to be a person present at least in the daytime in the department. Not a person you have to call, but a person you can get to, really getting access to information and guidance; that would be one of the major things I have thought would perhaps do something. Because if you’re working on a medical department and you have a dying patient and you should take the phone and make a call for the palliative care team there is a barrier there. So, letting the patient die without putting on a standard protocol would be much easier than calling for help … Yes, this accessibility of the competence of the experts, it should [not] be only a phone number; it should be a real person. (Dr Svend Otteson, Interview 16)

This was a point echoed by Professor Miyashita in Japan, when he described the extent of the training challenge and the lack of availability of specialist palliative care support, which meant that a pilot project to implement the LCP into general hospital wards had to be stopped:

…if (the) care team could support them and discuss or be contacted about the LCP every day, it might have worked. … And that education and support is … was very important … at that time, we stopped the pilot test at university hospitals there. (Professor Miyashita, Interview 10)

Interviewees from Norway and New Zealand offered a set of reflections about the infrastructural pre-requisites that they had come to realise were necessary to ensure the safe implementation of the LCP. In Norway, Professor Dagny Faksvåg Haugen described the development of an implementation protocol involving three key people: responsible manager, physician and nurse, who all sign the registration document and are responsible for the implementation process. She expressed the belief that in addition to the safeguards provided by the latter structure, the quality of health care provision in Norway is such that many of the problems experienced in the UK would be unlikely to occur. Norway modelled its implementation protocol on arrangements in New Zealand, as our respondents there made clear:

Yes, and the strategies that we put in place from a governance level, so we had governance over the work that we were doing and trying to keep those principles in mind. It’s probably worth pointing out as well that when you interview in Norway, Norway very much liked the way in which we had set up the national office and the coordinating and the LCP facilitators and very much copied that model very successfully to this day, I believe. (Dr Simon Allan and Bridget Marshall, Interview 2, SA speaking)

In most countries (except for Italy and Japan), the use of the LCP in some form continued after the cessation in the UK, but the terminology used to describe the intervention changed significantly. There was an overarching concern to avoid language used in the UK such as ‘putting patients on the pathway’. Moreover, in all cases, the term ‘LCP’ was abandoned. For example, in Austria, Dr Elisabeth Medicus reported that following the withdrawal of the LCP in the UK and her careful reading of the Neuberger report, she was at pains to ensure that the term ‘LCP’ was not used (as had begun to be the case) as a shorthand to categorise patients in the last days of life. Similarly, in Norway, a revised ‘plan’ was introduced called the ‘Last Days of Life’. Freedom from copyright obligations previously imposed by the Liverpool ‘home’ team also meant that such alterations were now possible:

But then after some years we revised our document. So at that time we made a lot of changes really. And I think that's a natural thing to do because you implement something and then you get feedback from users. And we’ve also done some studies … People have contacted us with comments. But we used all the experiences and evaluations from all the projects and studies, and made some changes to the new plan which is called the Last Days of Life. And then it was no longer a copyright document, so we were free to do that. (Professor Dagny Faksvåg Haugen. Interview 5)

Some interviewees offered final reflections on their hopes for the future. Many continued to work with the Liverpool team through new international collaborative ventures, in an effort to take forward their joint efforts to improve end of life care. We conclude with two examples here. Dr Stanley Macaden in India reported participating in the ‘International Collaboration for Best Care of the Dying Person’ initiated by the Liverpool team in 2014. He saw this as ‘the LCP in its new form’, with participation from India as an important aspect of wider efforts to improve end of life care on the sub-continent. Similarly, Professor Voltz from Germany looked forward to international collaboration with the Liverpool team, in spite of his clear criticisms of the LCP in its original form, concluding that:

… they have to be congratulated on putting up this difficult topic of care of the dying with their endeavours, despite everything I [have] said. It's really to be congratulated there and such an important topic. Which is why it still continues being in this group as well and trying to move things in, as I see, the right direction.’ (Professor Raymond Voltz).

## Discussion and synthesis

The second edition of the LCP handbook, published in 2011, contained a new final chapter, on international development ^33^. It gives insight into the principles that underpinned this programme of work and some of the settings where it was taking place. It is the only extended statement, from the proponents themselves, about the process and organisation of the international spread of the LCP.

The chapter begins by rehearsing the received-wisdom history of the modern hospice movement, its role in fostering the specialty of palliative care and the struggles of that speciality to establish an evidence base to underpin its work. It draws attention to areas where development has been slow and highlights the value of determining best practice for a well-defined group of patients over a well-defined period of time. The LCP is seen as a vehicle for this. Whilst it acknowledges the challenges of LCP adoption in relation to local cultural norms, policies and procedures, and clinical governance and risk frameworks, the chapter highlights an over-arching unifying factor – ‘the shared dedication and need for change to provide all of us with a dignified death’ (p.190).

From the year 2000 this had led the LCP Central Team in Liverpool to work with colleagues from several countries to implement the use of the LCP. The work prompted the creation of an LCP International Programme focussed, as in the UK itself, on four phases of activity: 1) Induction 2) Implementation 3) Dissemination and 4) Sustainability. In each case there are clear requirements and prescriptions spelled out in the 2011 chapter for how the work should proceed. The orientation is generally towards a specific local organisation in which LCP is to be introduced. There is almost no reference to system change, ‘roll out’ or strategic plans for more extensive settings.

Induction requires ‘top down and bottom up’ approaches and a ‘major cultural shift’ in the relevant organisation. Registration with LCP Central is important, with attention to branding, intellectual property and copyright. A local steering group is likewise key to taking the project forward and establishing the aims of the programme – to empower ‘generic workers’, improve care with demonstrable outcomes for the dying patient and relatives and to see care of the dying as part of the core business of the organisation, with its own quality markers. This in turn requires the endorsement of local translations by LCP Central, in accordance with established procedures. A successful programme will need a robust approach to education and training, which must be locally driven, but supported with materials from the Central team. Success will be more than mere numbers of usage, but must lead to changes in knowledge, skills, attitudes and confidence, as well as the physical environment and associated facilities. Research should be part of assessing this. There should be attention to governance and risk, and rigorous use of the core LCP document, with its goals of care unchanged. Careful documentation of ‘variance’ in achieving the goals is required, including examples where the goals were not deemed to be part of local practice.

The chapter is a remarkably clear statement of purpose, together with detailed *modus operandi.* It highlights three key elements: implementation, dissemination, sustainability.

*Implementation* into pilot sites should ideally follow an algorithm of ‘plan’, ‘do’, ‘study’, ‘act’ to foster continuous learning and some measure of whether improvement has occurred. Eighty percent of local staff should take part in an education programme about LCP before it is first introduced. Periodic status reports should be supplied to LCP Central.

*Dissemination* is key to successful implementation. Data from the first 20 uses of the LCP must be carefully stored, reviewed with the help of the Central team, and then shared. Dissemination should also involve attention to future research, learning and teaching plans, and possible management strategies for extending LCP beyond the pilot site.

*Sustainability,* in keeping with the local emphasis, is seen to occur when the LCP Continuous Quality Improvement Programme, duly agreed with LCP Central, is embedded across the local institution or the ‘local health economy’. But the value of establishing a state, country, or national office for LCP, fully endorsed by LCP Central is also described - perhaps as the pinnacle of sustainability.

Against the background of this 2011 account, we set out in 2017 to dig deeper and to answer a number of questions about the international spread of the LCP. Writing three years later, what lessons can we draw from the sources we have gathered and what light have we shed on the questions posed?

We have presented here the results of our literature review and qualitative interviews. Numerous overlaps and consistencies can be seen in the two analyses, as well as differing preoccupations. Taken separately we regard the two data sets as complementary and mutually reinforcing. In the interview analysis we found six major themes, which also ran through the body of literature we reviewed. We gained insights into the context and motivation of actors and agencies outside the UK for getting involved with the LCP and its implementation in new settings. Both data sets give accounts of the issues of translation and adaptation to new contexts as well as patterns of LCP deployment and resulting diffusion. The research literature, published commentaries and the interviews all give insights into the perceived benefits, as well as the challenges and drawbacks of using the LCP. The later papers and the interviews in general say something about the consequences for other countries that had become involved with it, of LCP withdrawal in the UK. The six themes identified in the interviews in turn echo and overlap with the original research questions that we set out, drawing from Dolowitz and Marsh (2000). We now explore the extent to which these questions have been answered, and in the process we weave in some specific and related questions that arose from our analysis, concerning the international spread of the LCP. Before that however, we acknowledge some limitations to our study.

### Limitations

Our purposive sampling led us largely to a group of LCP enthusiasts. If some had stepped back to evaluate the pathway in detail, it was always from the perspective that it had *prima facie* merit. It is quite possible that critical perspectives on the LCP may exist in the countries we studied. However, with one exception, there was no evidence of this in the literature review. It was difficult therefore to identify a process that would lead us to such dissident voices. Additionally, our interviews covered small numbers of individuals, who arguably, made up the LCP elite in the 14 countries. Their views may well differ from ‘rank and file’ perspectives within the wider clinical and research workforce. Finally, we have interviews from just 14 countries, whereas the literature revealed interest in six other jurisdictions, from which we have no first-hand accounts. Acknowledging all this, our study provides the biggest literature review yet undertaken on the use of the LCP outside the UK (covering 20 countries) and combines it for the first time, with interviews with key actors in the implementation process (from 14 of these countries).

### Who were the key actors involved?

From its point of initiation, the LCP formed part of a discourse concerning ways to improve the generic care of dying people, across multiple care settings. It was about taking the lessons learned in hospices and specialist palliative care settings and ‘scaling up’, possibly across a whole healthcare system, to include hospitals, care homes and domiciliary services. This was the stimulus for policy transfer to other countries, particularly because in the UK the LCP had gained considerable policy traction and become a key element in the national strategy for palliative care.

Despite its underlying policy gaols however, the key actors who became involved in LCP promulgation were typically clinicians, in particular specialists in palliative care, as well as both clinical and non-clinical researchers. The clinicians and researchers were in many cases individuals interested in system change, in finding ways to promulgate palliative care principles in settings where specialist knowledge was often absent. They were not short on enthusiasm about LCP, and can be seen at times evangelising about its benefits, but as we saw in our earlier paper, this could make for vulnerability to hubris and perhaps a disinclination to contemplate the risks or limitations of LCP adoption. Their concerns were mainly with demonstrating the efficacy of the LCP, rather than mitigating the unintended consequences that might result from its deployment. Italy is perhaps an exception to this, where there was a strong view that specialist palliative care teams must be involved in directing and supporting the implementation of LCP in other settings if good results were to be obtained.

The Netherlands was the first country outside the UK to engage in detail with the LCP. This was no accident. The initiator and leader of the LCP programme in the UK, John Ellershaw, had been a visiting professor at the Erasmus University in Rotterdam from September 1997 and had been active in sharing his LCP work with Dutch colleagues. Ellershaw published a paper on LCP in a Dutch language journal in 2002 ^45^ and co-authored abstracts that were presented at the 8 ^th^ Congress of the European Association for Palliative Care (EAPC), held in the Hague in 2003 ^46, 47^. The work on the use of LCP in the Netherlands is probably the only example where Ellershaw was directly involved in the research process and outputs, which were the second most numerous for any single country, and sustained over the longest period of time.

Initially, in many cases, it was this kind of *ad personam* link that stimulated the diffusion and transfer of LCP in other places. Many of the key actors were participants at EAPC and other international congresses and built up an informal network of mutual interest. This quickly became bureaucratised and formalised as ‘LCP Central’ developed a controlling role in transfer and translation, and it was further consolidated through the OPCARE9 collaborative. Only in a few settings, such as Norway and New Zealand, and to some extent Australia and Sweden, was there a sense of LCP being implemented as a result of a prior and wider policy commitment to end of life improvement. In most instances, the actors involved got to work on trying out the LCP in their local context, and where they saw opportunities they then tried to work LCP into the policy language and architecture of their jurisdiction. We might think of the LCP more as a ‘Trojan horse’ than a ‘step change’, seeking to alter a system from within and below, rather than imposing a blueprint from above. LCP Central support seemed more focussed on the clinical organisational setting for implementation than on the wider policy environment. There was a striking absence of policy makers and policy researchers in the authors of the papers we reviewed and the people we interviewed. LCP protagonists could therefore be seen as consistent with Benson and Jordan’s sense of those frustrated with current attempts to develop and implement new policies and who were searching for alternative ways to bring about change ^22^.

Sometimes actors sought strength for this in bi- and tri-lateral collaborations between countries within the overall group of 20. For example: colleagues in Spain and Argentina worked together and produced some joint publications and presentations; a German language group from three countries formed a collaborative; Norway appeared to draw on experience and approaches from New Zealand; colleagues in Belgium and Italy collaborated in detail on research design and publication. In several instances, LCP Central in Liverpool used its networks to create wider collaborations with actors in different countries. Likewise, the existence of receptive networks within a country could help to spread LCP awareness and implementation. This was found in strength in Sweden, Norway, New Zealand and Australia, Italy, Belgium and the Netherlands; it was somewhat apparent in Germany, Switzerland and Austria, as well as Japan; but largely absent in Argentina, Spain, India, China, Ireland, Denmark, Singapore, Hong Kong and Slovenia.

At the same time, we can see evidence of ‘push and pull’ between the UK actors and those in other countries. There was a ‘push’ outwards from the LCP Central team through OPCARE 9 and its attempt to exert ‘version control’ and to standardise reporting processes. Yet there was also a ‘pull’ from the other countries, that is exposed in both elements of our data. In the interviews, various motivations and stimulations were revealed, ranging from widely shared aspirations to improve quality of end of life care by applying a structured approach, to research oriented rationales, where the LCP was seen as an opportunity to expand a research programme. In Italy, there was a desire to evaluate the LCP using a rigorous methodology not previously applied, and to establish whether it had benefits in hospital care. In Belgium, a research funding opportunity seemed to be a key motivation. In the literature review, we can see the ‘pull’ manifested in the enthusiastic marshalling of stakeholders who were eager to try out the LCP in different settings and organise audits and research studies to cast light on the effects.

The key actors involved in the transfer process were therefore to be found in many different roles in varied types of organisation, ranging from hospices, care homes and domiciliary teams to major teaching hospitals. They were primarily clinicians, with some service managers. They also included palliative and end of life care researchers. They in the main did not include policy makers and implementation scientists. As measured by their published outputs, less than half of the countries identified produced research evidence in quantity (81% of the total outputs reviewed) and of significant quality: Japan, Netherlands, Italy, New Zealand, Australia, Sweden, Belgium, Germany, Austria.

### What was transferred?

Steiner ^48^ (p 246, cited in Freeman 2009) notes a ‘radical tension’ in policy transfer between the impulse to implement an exact facsimile of the chosen intervention and the impulse to somehow recreate or modify it. Our study reveals this tension very clearly. As we noted in the review of our interviews, emphases varied from a focus on a precise and exact translation of the LCP to using the LCP as a framework or set of principles, sometimes only referred to in national guidance and not implemented as the pathway itself. Many countries tried to strike a balance between these positions, but few actors seemed to consider the implications of their chosen orientation. We observed in our original paper on LCP, that boundary objects are most effective when subjected to local reinterpretation. In some countries local interpretations were seen as a potential problem (Netherlands, Japan); in others (Switzerland, New Zealand, Australia, Argentina) they were viewed as both necessary and beneficial.

Translation is at the heart of this, both linguistically and culturally. Some struggled to achieve a close translation of LCP into another language, and expended significant energy on the forward and back translation approaches as well as in achieving linguistic integrity, for example where particular verbs or nouns did not exist in translation (Japan, Netherlands). Cultural adaptation was visible in several ways, in the creation of new names and acronyms for the LCP (Belgium, Netherlands) and most notably in Argentina, where the chosen acronym had a distinct relevance (PAMPA) to local culture and imagery. In other cases (Australia, New Zealand), there was no significant issue about linguistic translation as the English language was a common factor, but actors did refer to system adaptation, to strengthen the fit with local healthcare practices and procedures. Hong Kong and Singapore however, seemed to combine all three elements of translation (linguistic, cultural, systemic), by translation into the relevant language and also changing some of the key components for local relevance or significantly cutting the number of goals on the pathway.

We noted in our first paper that LCP in the UK was surrounded by ambivalence about its purpose and make up, namely whether it was a document for close attention and implementation, or a broad approach to caring at the end of life, and we also explored whether it encouraged person centred care or standardisation of care processes across a group of patients. This ambivalence was also seen internationally. In some countries (Italy and Belgium, and to a lesser extent Japan), LCP was clearly framed as a complex intervention with several inter-related components and in which its ‘support’ elements, notably the implementation manual, became crucially important. Actors in other countries focused more narrowly on the introduction of the documentation and its use, without extensive supporting activities, although all were aware that training and education were crucial. These differences were reflected in the types of data gathered for research and evaluation. Some studied its impact on patient or family outcomes primarily, while others focused on audit data, especially: ‘variance’ of recording against the LCP documents or types of document prompted by the introduction of the LCP; rates of use; staff views, experiences, perceptions/ meanings; or process issues such as communication between clinicians.

The LCP was first developed within the context of the care of terminally ill cancer patients, so in its international spread these patients initially figured prominently, especially in implementation and research that took place with the involvement of specialist palliative care teams. Sometimes, as interest in the LCP gained momentum, the patient groups expanded accordingly, to include patients with non-cancer conditions. Others saw LCP from the outset as a vehicle for broadening the reach of patients who could benefit from its structured approach, in particular older people. When this occurred in Belgium, it required significant adaptation to the documentation of LCP. When this was not undertaken, as in Norway, it brought forth criticism from those who considered LCP inappropriate for patients with dementia in nursing homes. In some instances (Germany, Sweden), the paper-based format of the LCP looked outdated and did not facilitate integration with a health care system based entirely on electronic records. 

The LCP as a document was assiduously transferred elsewhere with relatively minor variations, mostly sufficient only to justify adoption in a new context, perhaps marked by the use of a new name of acronym. This integrity was then leveraged to move LCP into a wide range of settings, supported by a wide variety of agencies and actors across the 20 countries. As this happened, the boundaries around the LCP could be more blurred. LCP Central sought to avoid this and maintain its integrity in various ways. In some countries, where no formal abandonment of the LCP took place (in particular Norway and Sweden) it took on its own momentum, and the relevant actors even suggested their work was made simpler in the absence of the controlling hand of LCP Central. Almost universally agreed however, was that LCP contained a structured approach to end of life care, which was successfully transferred to numerous settings, where it gave a framework for successful care in the final days of life. This could in turn have a wider influence, inflecting policy documents and guidelines with its underlying principles or providing content examples for the training of professionals who may never use the LCP but could learn from its content and goals.

### From where were lessons drawn?

We have seen that in several countries, where studies were carried out in the early stages of implementation, there was a tendency to interpret the results, however modest in scope, in a very positive light. Limited evidence did not prevent many key actors from being caught up in what might be termed a *habitus of optimism* that could not countenance poor results or outcomes. The early studies in the Netherlands are good examples of this. Where the research designs were more sophisticated (as in Italy) they could also be prone to over-stating benefit, such as in quasi-experimental before/after studies characterised by two measurement points, one before and one after the intervention, without any external control group, and which were more likely to produce ‘benefit’ from the intervention.

The Italian and Belgian studies (one of LCP, the other of a close derivative) stand out for their systematic approach and a stronger sense of wanting to test the intervention, eventually resulting in a randomised control trial, albeit in both cases with unconvincing results from the LCP perspective. In Belgium there was also a particular emphasis on learning systematically from the LCP experience within the UK. In Italy and Belgium and to a lesser extent Sweden, the Netherlands and Japan, there seems to be no distinctive body of work on the implementation of the LCP that builds knowledge and experience over time. In other settings (Spain, Argentina, Hong Kong, India, Singapore), the publications were limited in number and seem to set up a discussion and a level of interest based on early enquiries into the use of LCP, that was not followed up with further research or documentation (except in Argentina) to indicate how things developed in the longer run.

It is perhaps more useful to see LCP as successful in process terms. By highlighting the need for better care at the end of life and the challenges of diagnosing dying, stimulating education and training, helping to leverage new resources and policy change, forging international collaborations (all useful things), it provided a point of focus in an area otherwise rather challenging and daunting for isolated individuals to make any kind of improvements. Yet against the gold standard of RCT evidence, LCP was unable to provide convincing benefit. In the UK there was much anecdotal commentary that LCP could do harm; but this was not shown or even hinted at in the studies of its international use, though in Germany there was an interest in patients on the LCP who did not die and the clinical implications of this.

Another ‘process’ dimension was the way in which the LCP helped to foster international collaboration for clinicians that was perhaps unavailable to them in any other form in a still emerging and evidence-challenged field of specialisation. The enthusiasm with which they engaged with it, and their disappointment and anger when it was attacked in the UK press and government, is therefore understandable. 

Most countries were unable to engage with the ‘plan, study, do, act’ cycle of implementation and several did not conduct any extensive level of evaluation or research. Rather, they seemed to rely on the wave of international interest in the LCP, seeing this as somehow sufficient as a recommendation for use. They placed a high value on the UK/Liverpool origins of the LCP and its creation by a prominent professor in the field, who had indeed trained with Dame Cicely Saunders, and who in turn had endorsed the LCP in her foreword to the handbook, first published in 2003. Later in the cycle, some, through their own observations or attempts to introduce LCP beyond their own setting, came to realise its limitations, in time reading the ‘Neuberger’ report for ideas that would pre-empt similar issues in their own jurisdiction. Some remained puzzled by what had occurred in the UK and perceived no deleterious consequences for the use of the LCP. But others, like Italy, stopped short of introducing it more widely or actively encouraging its growth, even though its effects seemed useful, if not as much as hoped.

The lessons were drawn from several sources. Initially these focussed on specific experiences in the participating countries, which were shared via LCP Central, OPCARE9, or in wider f *ora* such as conferences and publications. In time a body of publications appeared that individually and severally offered numerous lessons, mainly at the micro-level of implementation. Macro-level lessons were drawn by inference from the UK experience, leading reluctantly to the conclusion that if LCP could not work effectively there, then its long-term chances of success elsewhere were probably slim.

### What restricts or facilitates transfer?

The form of policy transfer we have been describing here is ‘voluntary’ rather than ‘compulsory’ in character. The various sets of actors collaborated through their own motivation, volition and desire to improve palliative care, rather than through any form of requirement, constraint or target to be met. Indeed, as we have seen, their goal was in part to foster a policy mandate for LCP, that if successful would have led to more compulsion about its use.

We need to distinguish between two elements within the ‘transfer’ described here: 1) the diffusion of ideas and practices around the LCP and 2) the ‘spread’ of LCP implementation. Both were mainly confined to the Global North. Successful sharing of ideas and practices however did not lead inevitably to spread.

It was in clinical settings that evidence around ideas and practices was most visible. Clinicians in all the countries we studied can be seen engaging with the LCP in ways that were fostered through international meetings, networks, journal publications and conference presentations. These undoubtedly facilitated aspects of transfer, and the formation of LCP Central in Liverpool and then of OPCARE9 provided a secure framework in which to foster these forms of knowledge exchange, albeit mainly within those defined as members.

It seems evident that a lack of material resources to access these networks served to restrict transfer and those who most easily gained entry to them were in some cases already prominent within the international palliative care field, established as clinicians and academics and members of groupings of collaboration that even preceded the advent of LCP. Only two sets of actors from low and middle income countries (LMICs) - Argentina and India - were able to benefit from such resources or alternatively, to see the LCP as relevant to their work. Additionally, where palliative care specialist knowledge was weakly developed or not widely available, then this generated problem for the successful use of the LCP in hospital wards, care homes and domiciliary settings.

The spread of LCP within a jurisdiction was also dependent on the support of other stakeholders beyond the adopters and champions of LCP. For example, in the Netherlands, critical to the scaling up was endorsement of LCP by the Comprehensive Cancer Centre of the Netherlands and then the ‘roll out’ in 66 regional palliative care networks. Key actors in Bergen gained support from the Norwegian Medical Association and then the local health authorities. In New Zealand and Sweden there were national LCP centres that took a lead. In Switzerland the process involved inserting elements of LCP into the principles and competencies contained in the national strategy for palliative care, rather than a concerted process of diffusion. In many countries there appear to have been examples of organic and un-regulated use of LCP ideas, unconnected to Liverpool Central, and that could be found in bespoke local and usually re-named variants that seemed to be well received and perhaps quite resilient, even when LCP was withdrawn in the UK.

Educational and workforce limitations restricted spread, as did the absence of a national framework for palliative care. Research or its lack did not seem to have a bearing on spread, although when research was conducted in its most rigorous form (in Italy) it eventually led to the conclusion that the LCP was neither sustainable nor safe unless implemented with ongoing and intensive specialist palliative care support, which was unavailable at scale. Actors in other countries reached similar conclusions, albeit through different routes (Denmark). In only one country (Norway) was there evidence of conflict between actors around the use of the LCP.

Transfer of the LCP was restricted by several factors, including high level policy commitment in the most of the ‘recipient’ countries, limited resources and still under-developed systems of palliative care, and the absence of research evidence that could be used to make a convincing argument for its adoption. It was facilitated by the work of LCP Central and the resources for collaboration that it marshalled, as well as the individual qualities and enthusiasm of actors who were eager to bring about system level improvements in end of life care. 

### How did transfer relate to ‘success’ or ‘failure’?

On the eve of the publication of the Neuberger report, John Ellershaw and his colleagues posed the question: ‘… as the debate continues in England, the LCP’s country of origin, could an international perspective provide the next steps in improving care of the dying?’ ^14^. The curious syntax belies the intention. If LCP was discredited in the UK, it might yet survive, modify, even thrive, elsewhere.

Early adopters outside the UK (like the Netherlands, New Zealand, Japan and Hong Kong) did not have the shadow of growing criticism of the LCP to contend with, at least initially. Italy was in the paradoxical position of publishing the most important RCT of the LCP several months after the Neuberger report had recommended its withdrawal. Others (Australia, Austria, India, Germany) were somewhere in between and were proceeding with LCP plans even as a ‘gathering storm’ of criticism was beginning in the UK from September 2009. Late adopters (Sweden, Norway, Belgium) did so even as a torrent of professional and public criticism was being heaped upon the LCP in the UK, from 2012 onwards, yet found success in its use.

It does not appear that the media storm which circulated around the LCP in the UK was replicated elsewhere. It is difficult to determine why this should be the case. As one interviewee noted, the ‘Murdoch press’, seen to be key to the UK attack on the LCP, is also present in Australia, where no such storm occurred. In Norway there was robust opposition to LCP from one quarter, but this did not escalate in the manner seen in the UK. Rather the reactions elsewhere to LCP withdrawal in its home base were disbelief, annoyance, puzzlement and regret. In Italy, LCP seemed poised for wide adoption, but the equivocal results of the RCT, coming hard on the heels of the Neuberger report, led to a considered and regretful decision to abandon it. The main consolation here was that the studies conducted in Italy had demonstrated that research can be done among people in the last stages of life and can produce insights relevant to policy and practice.

There can be no definitive answer to this question of how transfer relates to ‘success’ or ‘failure’.

Across the 20 countries, many successes can be seen to result from engagement with LCP. It fostered new collaborations and partnerships which proved enduring even when the LCP work tailed off. It created significant awareness of the need for a structured approach to end of life care that could be operationalised across skill levels, professional groups, and care settings. It was reported as beneficial in some way by many studies, none of which suggested it did harm. It was widely accepted and liked by caregivers in many capacities, and whom found it supportive.

If it failed it did so on two fronts. First it rarely gained the policy recognition and investment it needed to become established and integrated at the level of the care system. It remained mainly in the domain of those who were enthusiastic about it, but with a few exceptions, it did not diffuse far beyond the pilot sites. Second, it lacked a cumulative evidence base that fully recognised its complexity as an intervention. The highest quality studies were conducted in only three countries, and in no instance was there an overwhelming case for the success of the intervention, but it was only in one of these (Italy) that the pathway was abandoned.

The transfer and translation of the LCP to 20 countries beyond the UK had several underlying properties. It contained elements of reciprocity in the giving and receiving of an idea, in the sharing of its subsequent modification and development, and in the actions needed to evaluate the outcomes. These reciprocal actions were voluntary and not mandated. The actors’ own agency drove the quest for new knowledge, skills and improvement in end of life care. The spread of the LCP took place in defined spaces, mostly in prosperous countries, and was sustained over around 15 years. It took in differing geographies and cultures, and a variety of linguistic, policy and practice contexts. If it did not succeed in a wider transformational goal, it appears to have been well received and perceived as beneficial in many contexts. Those who promoted the international spread of the LCP created a set of values around it akin to a ‘culture’ or ‘movement’ which went beyond its technical dimensions. It was not reported to have done harm and it did not generate major public concern or scandal. Yet LCP was not fully established in any of the 20 countries and in some only achieved the most tentative foothold. But in several settings its influence could be seen in policy documents, inspired ‘spin offs’, or frameworks for education. This also begs the question ‘what is the LCP?’, which nobody seemed to agree upon. Its protagonists, led by the Liverpool team conducted damage limitation after the withdrawal of the LCP in the UK, maintained the interest of the international group members, and quietly re-labelled the work, in particular to avoid the contested idea of an end of life ‘pathway’.

## Conclusion

If 20 countries outside the UK experimented with LCP, it still left more than 170 that did not. This was not just about resources. It is difficult to know why there was no move to adopt the LCP in the USA, given that much ‘pathway’ thinking originated there, though some American clinicians were quick to comment on where LCP had gone wrong in the UK. Nor did countries such as Canada, South Africa, or France (all with long histories of palliative care development) emerge as nodes for LCP implementation. There was however a synergy between interest and development relating to LCP and the overall level of palliative care in a country. A study based on data from 2017 showed 30 countries, mainly in the global north, to be in the highest level of palliative care development. Of these, 15 were countries that had shown interest in LCP, per our literature review ^49^. We might conclude from this that LCP had appeal in rich countries where palliative care was already well developed, but seemed of little attraction where the reverse was the case. Even so, our interviews show that diffusion of LCP *within* the countries studied was also extremely variable. If the LCP in Denmark and Austria was confined to just one specific local service, in Norway, New Zealand, Australia, Sweden and the Netherlands, and sometimes with the support of a national co-ordinating centre, a degree of national spread took place, sometimes within a comprehensive range of patient groups and care settings. Many other countries were somewhere in the middle with a degree of take-up regionally, or locally.

Among the 20 countries included in our review, less than half made significant traction with published outputs and higher level research studies. Yet in these global north settings, with pre-existing palliative care infrastructures, it did prove possible to conduct experiments with LCP and where favourable initial results emerged, to build alliances that would support its implementation within local or even regional jurisdictions. Even where this advanced to a high level, such as in the Netherlands, unlike in the UK, it did not generate wider criticisms or concerns. The LCP never obtained in other countries the level of national policy endorsement that it gained in the UK – and this, paradoxically, may have protected it from criticism. Only in Norway, where LCP came to be widely used in nursing homes, did LCP come under critical scrutiny from specialists in the care of older people, who saw it as unsuitable for use for people with dementia.

If our analysis tells us something about the transfer of palliative care interventions internationally, it demonstrates that such transfer can be achieved with discrete measureable successes, rather than in ways that fully endorse the intervention *in toto*. Our analysis confirms Freeman’s point that in the translation process, a boundary object may allow a wide variety of actors to come together to communicate about a problem, even when – as in end of life care – there is still a lack of consensus about how a solution should be delivered, to who and with what expectations. In this way the LCP provided an international point of focus and action to try to improve end of life care, even though the actors involved often had different ideas about it and used it in a variety of ways.

Central to this was the almost universally recognised point in the literature and among our interviewees that LCP provided a structured approach that could give direction and confidence to practitioners as they entered into the often obscured and clinically charged areas of care in the very last hours and days of life. This manifested itself in new approaches that were given further encouragement by involvement with the LCP, to improve: advance care planning, interdisciplinary communication, and aspects of nursing practice. But there were also drawbacks and costs. Effective implementation of LCP required investment in education and training for those who would use it. There were also funding challenges, as well as issues relating to staff turnover, which could hamper progress. Likewise, difficult decisions had to be made about how far a standardised approach should be taken to LCP, and to what extent its successful implementation required local variation. Sometimes new care ‘plans’ emerged that were inspired by LCP but were often formulated quite differently. Many actors adapted their work on the LCP into the healthcare system in which they were located. This could mean less rigidity about the terms of reference, a more sophisticated approach to implementation, or simply taking the best ideas from the LCP and building them into clinical mentorship, education and training.

Yet ultimately, the structured approach that was so much valued seemed to bring very little measureable improvement when tested out in controlled studies. The entire LCP international initiative saw such studies in only three countries. In each case the measureable benefits were very small, restricted in Sweden to two symptoms (shortness of breath and nausea), in Italy to improvements in respect, dignity and kindness and in the control of breathlessness. In Belgium a negative effect emerged from the RCT, relating to reduced satisfaction with care among family members. Such stark results should perhaps not obscure the evident acceptability of and enthusiasm for LCP on the part of many of the professionals and service users who encountered it, but they do seem a meagre outcome from so much effort. Withdrawal in the UK did not prevent continued use of LCP ideas in many places, but it did kill off the fledgling research agenda, which was about to be taken to the next level, for example in Italy, by focussing on the elements within the LCP which were beneficial or not.

Some of those involved with it internationally were shocked and confused by the withdrawal of LCP in the UK and expressed this in strong terms. Yet in several instances, through the knowledge transfer of OPCARE9 and the wider LCP network, some countries had been able to mitigate against the problems seen in the UK – building in a greater emphasis on training, focussing more on implementation principles, and ensuring good governance. For some the post-Neuberger era even meant a removal from the constraints imposed by LCP Central, and greater freedom for self-determination locally. Only Italy and Japan abandoned the LCP completely after its UK withdrawal.

What then does this analysis tell us that we did not know before about the transfer of palliative care interventions internationally?

Conceptually we can identify five dimensions that shaped the transfer of the LCP from its country of origin to 20 jurisdictions beyond the UK and which need further attention in future work.

1) We need to understand policy transfer in a period of *historical time*, in this case ** when the discipline of palliative care was seeking to gain traction and promote its knowledge and methods beyond the specialist services in which it had originated.
2) The transfer is in turn shaped by particular *actions and processes* that in our worked example sought to promote, implement and validate the LCP as a transformative care pathway for the end of life.
3) To do this LCP had to be lodged within *systems* in which it could operate effectively and deliver measureable change.
4) How this was achieved depended significantly on the *cultures, geographies and settings* in which it was placed, and these could take many forms.
5) Success in all of this required clarity about *goals, outcomes and related consequences*.

These five dimensions may well form a useful checklist for others to consider as they contemplate embarking on the extensive transfer of an end of life care intervention across multiple jurisdictions. They serve as a useful antidote to the more formulaic algorithm of ‘plan’, ‘do’, ‘study’, ‘act’.

If we judge the 20 countries included in this study against the yardstick of the UK, then collectively their encounters with LCP can be regarded as moderately successful. First and foremost, no scandal resulted, as had been the case in the UK. There was no almost evidence for or claims about patient harm. Beyond that helpful alliances and understandings were fostered, as disparate practitioners in varied settings made common cause in a bid to improve the quality of dying and saw the LCP as helpful in this process. Researchers worked diligently to produce robust study designs and useable results. Almost 100 published outputs described aspects of these processes and what had emerged from them. By 2017, the key actors were still working together on improving care in the last days of life, but LCP as a complex intervention, with all its associated systems and machinery, had faded from view. Its legacy remained, some even continued to mourn its passage or to want further discussion of what had occurred, but most, not least its creator, wanted to close that particular chapter in the development of palliative care.

We have noted elsewhere that understanding an end of life intervention should include some account of the motivations of its instigators, the processes of its implementation, the field of discourse in which it is located and the presence or absence of unintended consequences relating to it ^50^. We have tried here to capture these elements in relation to the LCP, through our literature review and our interviews. We remain concerned that the field of palliative care continues to be unduly committed to finding workable models that can be quickly scaled up and transferred across jurisdictions, as seen in the approach taken by a recent Lancet Commission with its ‘essential package of palliative care medicines, basic equipment, and human resources that could alleviate much of avoidable suffering in LMICs’ ^51^. We contend that alongside this approach something more nuanced is also required, which acknowledges actor motivations, takes account of transfer and translation, can make balanced judgements about equivocal research findings, and at the same time gain the ear of policy makers and funders.

Our literature review and our interviews surfaced many statements about the importance of ‘implementation’ when it came to understanding the story of the LCP. Four key elements in a theory of implementation have been mapped out by May ^52^. He describes 1) the involvement of agents in the intentional modification of social systems that occupy a field of action, 2) expressions of agency that shape and are shaped by their context, 3) these in turn interact with endogenous and exogenous contingencies and confounders, and 4) they must then be negotiated by the agents involved who seek to shape them in ways that can govern the process and its outcomes. May’s approach is ‘founded on the notion that implementation expresses “agency” and should be understood and evaluated against the problem of how human agents take action in conditions of complexity and constraint’(p2). To paraphrase this approach, rather than seeing the LCP as a ‘thing’ to be implemented, it is better understood as a set of practices, with varying degrees of workability and multiple opportunities for deployment, and with the strong likelihood that the actors involved will shape expectations surrounding and conditions of its use.

The international initiative around the LCP demonstrates the saliency of all these interacting elements. Overall, it led to changes in policy and practice in certain jurisdictions and it fostered a sustained international collaboration that continued after the abandonment of its use in the UK. LCP was maintained in modified form in certain settings, and it largely avoided accusations of misuse and harm that had occurred in its country of origin. If ultimately it did not succeed in promoting transformational change in the end of life care systems of the countries that engaged with it, nevertheless its influence remained, and as one of our interviewees reported when writing to us just as this paper was completed – in 2020 it even ‘rose out of the ashes’ to influence hastily formulated palliative care strategies for generalist settings, in the face of COVID-19.

## Data availability

### Underlying data

The full underlying transcripts contain many passages that are confusing, in poor English and are difficult to understand. In fairness to our interviewees we therefore opted to include the full analysis of the interviews as underlying data ^28^, rather than the full transcripts. We gave the interviewees an opportunity to check their statements to make sure that the analysis reflected the full interviews performed. Therefore, although the full transcripts are not provided, the analysis contains all relevant information required to reproduce the analysis performed in the study.

Enlighten Research Data: International transfer and translation of an end of life care intervention: the case of the Liverpool Care Pathway for the Dying Patient, http://dx.doi.org/10.5525/gla.researchdata.1067 ^28^.

This project contains the following underlying data:

- Full literature review with full reference list
- Full analysis of interviews

### Extended data

Enlighten Research Data: International transfer and translation of an end of life care intervention: the case of the Liverpool Care Pathway for the Dying Patient, http://dx.doi.org/10.5525/gla.researchdata.1067 ^28^.

This project contains the following extended data:

- Consent for interviewees
- Information sheet for interviewees

Data are available under the terms of the Creative Commons Attribution 4.0 International license (CC-BY 4.0).

## Footnotes

^i^ HARDY, J. R., HABERECHT, J., MARESCO‐PENNISI, D., YATES, P. & AUSTRALIAN BEST CARE OF THE DYING NETWORK, Q. (2007). Audit of the care of the dying in a network of hospitals and institutions in Queensland. *Internal Medicine Journal,* **37**(5), pp. 315–319.

^ii^ LO, S. H., CHAN, C. Y., CHAN, C. H., SZE, W. K., YUEN, K. K., WONG, C. S., NG, T. Y. & TUNG, Y. (2009). The implementation of an end-of-life integrated care pathway in a Chinese population. *International Journal of Palliative Nursing,* **15**(8), pp.384–388.

^iii^ VEERBEEK, L., VAN ZUYLEN, L., GAMBLES, M., SWART, S. J., VAN DER HEIDE, A., VAN DER RIJT, C. C. D. & ELLERSHAW, J. E. (2006). Audit of the Liverpool Care Pathway for the Dying Patient in a Dutch cancer hospital. *Journal of Palliative Care,* **22**(4), pp. 305–308.

^iv^ VEERBEEK, L., VAN DER HEIDE, A., DE VOGEL-VOOGT, E., DE BAKKER, R., D. VAN DER RIJT, C. C., SWART, S. J., VAN DER MAAS, P. J. & VAN ZUYLEN, L. (2008a). Using the LCP: bereaved relatives' assessments of communication and bereavement. *American Journal of Hospice and Palliative Medicine,* **25**(3), pp. 207–214.

^v^ GROSSENBACHER-GSCHWEND, B & EYCHMÜLLER, S (2007). Der Liverpool Care Pathway of the dying. [The Liverpool Care Pathway of the Dying] *Der Onkologe*, **13**(4), pp.343–349.

^vi^ KOON, O. E., NEO HUI SHAN, S., SHIVANANDA, S., YING, T. Y., THANG, A., KYAWT, A. M., SANTOSO, U., YIN, G. P. S., CHUNG PHENG, A. Y. & NEO SOEK HUI, P. (2015). Use of a Modified Liverpool Care Pathway in a Tertiary Asian Hospital: Is There Still a Role for It? *Journal of Palliative Medicine,* **18**(6), pp. 56–512.

^vii^ NAKAJIMA, N. (2010). 一般病棟におけるLCPの使用経験と利用のコツ [Experiences and tips of implementing LCP in general wards]. [Special issue]. 臨牀看護 *Japanese Journal Clinical Nursing*. 36, pp.1862–68.

^viii^ SWART, S., VELUW, H., ZUYLEN, L., GAMBLES, M. & ELLERSHAW, J. (2006). Dutch experiences with the Liverpool Care Pathway. *European Journal of Palliative Care,* **13**(4), pp. 156–159.

^ix^ VEERBEEK, L., VAN ZUYLEN, L., GAMBLES, M., SWART, S. J., VAN DER HEIDE, A., VAN DER RIJT, C. C. D. & ELLERSHAW, J. E. (2006). Audit of the Liverpool Care Pathway for the Dying Patient in a Dutch cancer hospital. *Journal of Palliative Care,* **2**(4), pp. 305–308.

^x^ SIMON, S. T., MARTENS, M., SACHSE, M., BAUSEWEIN, C., EYCHMÜLLER, S. & SCHWARZ-EYWILL, M. (2009). Sterbebegleitung im Krankenhaus–erste Erfahrungen mit dem “Liverpool Care Pathway” (LCP) in Deutschland. *DMW-Deutsche Medizinische Wochenschrift,* 134, pp. 1399–1404.

^xi^ TRIPODORO, V. A., LUXARDO, N., VELOSO, V., ET AL (2013). Implementación del Liverpool Care Pathway en español en Argentina y en España: exploración de las percepciones de los profesionales ante el final de la vida. *Medicina Paliativa,* **22**(3), pp. 16.

^xii^ MACKENZIE, T., INNES, J., BOYD, M., KEANE, B., BOXALL, J. & ALLAN, S. (2011). Evaluating the role and value of a national office to coordinate Liverpool Care Pathway implementation in New Zealand. *International Journal of Evidence-Based Healthcare*, **9**(3), pp. 252–260.

^xiii^ EKESTRÖM, M.-L., OLSSON, M., RUNESDOTTER, S. & FÜRST, C. J. (2014). Family members' experiences of the impact of the LCP in a palliative care unit and a geriatric ward in Sweden. *International Journal of Palliative Nursing,* **20**(8), pp. 381–386.

^xiv^ HØGSNES, L., DANIELSON, E., NORBERGH, K. G., & MELIN-JOHANSSON, C. (2016). Healthcare professionals' documentation in nursing homes when caring for patients with dementia in end of life - a retrospective records review. *Journal Clinical Nursing*, **25**(11–12), pp. 1663–1673.

^xv^ ANDERSSON, S., LINDQVIST, O., FÜRST, C.-J., BRÄNNSTRÖM, M. V. (2018) Care professional's experiences about using Liverpool Care Pathway in end-of-life care in residential care homes. *Scandinavian Journal of Caring Sciences*, **32**(1), pp.299–308.

^xvi^ ANDERSSON, S., LINDQVIST, O., FURST, C. J., & BRANNSTROM, M. (2018b). Family members' experiences of care of the dying in residential care homes where the Liverpool Care Pathway was used *. International Journal of Palliative Nursing*, **24**(4), pp. 194–202.

^xvii^ OLSSON, C., KLING, E., GRUNDEL PERSSON, K., & LARSSON, M. (2019). Impact of the Liverpool Care Pathway on quality end-of- care in residential care homes and home care—Nurses’ perceptions. *Nursing Open*, **6**(4), pp. 1589–1599.

^xviii^ FAROOQ, F SVENDSEN, C OTTESEN, DS (2016) Symptom relief in dying patients – how good are we? Support Care Cancer 24 (Suppl 1):S1–S249 S159. https://link.springer.com/content/pdf/10.1007%2Fs00520-016-3209-z.pdf

^xix^ ICHIHARA, K. (2010). LCPを臨床現場に適用する；緩和ケア病棟におけるLCP日本語版の導入[Applications of LCP in clinical settings: introducing LCP-J in palliative care units]. [Special issue]. 臨牀看護 *Japanese Journal Clinical Nursing*. 36, pp.1838–48.

^xx^ KANNO, Y., SATO, K., HAYAKAWA, Y., TAKITA, Y., AGATSUMA, T., CHIBA, T., HONDA, K., SHIBATA, H., YAMAUCHI, K., TAKAHASHI, S., INOUE, A. AND MIYASHITA, M. (2015). 一般病棟で看取りのケアのクリニカル・パス Liverpool Care Pathway日本語版を導入するための課題―大学病院での使用経験から [The burden of introducing the Japanese language version of the Liverpool Care Pathway（LCP-J）for dying patients in general wards and their families: experience of health care professionals in a university hospital]. *Palliative Care Research*, 10, pp. 318–23.

^xxi^ COSTANTINI, M., BECCARO, M. & DI LEO, S. (2011). Improving quality of end-of-life care. A possible and necessary change. *Epidemiologia e prevenzione,* **35**(3–4), pp. 229–233.

^xxii^ DI LEO, S., BECCARO, M., FINELLI, S., BORREANI, C. & COSTANTINI, M. (2011). Expectations about and impact of the Liverpool Care Pathway for the dying patient in an Italian hospital. *Palliative Medicine,* **25**(4), pp. 293–303.

^xxiii^ COSTANTINI, M., DI LEO, S., & BECCARO, M. (2011). Methodological issues in a before-after study design to evaluate the Liverpool Care Pathway for the Dying Patient in hospital. *Palliative Medicine*, 25(8), 766–773.

^xxiv^ COSTANTINI, M., PELLEGRINI, F., DI LEO, S., BECCARO, M., ROSSI, C., FLEGO, G., ROMOLI, V., GIANNOTTI, M., MORONE, P., IVALDI, G. P., CAVALLO, L., FUSCO, F. & HIGGINSON, I. J. (2014). The Liverpool Care Pathway for cancer patients dying in hospital medical wards: A before–after cluster phase II trial of outcomes reported by family members. *Palliative Medicine,* **28**(1), pp.10–17.

^xxv^ LEO, S. D., BONO, L., ROMOLI, V., WEST, E., AMBROSIO, R., GALLUCCI, M., PILASTRI, P., CIURA, P. L., MORINO, P., PIAZZA, M., VALENTI, D., FRANCESCHINI, C. & COSTANTINI, M. (2013). Implementation of the Liverpool Care Pathway (LCP) for the dying patient in the inpatient Hospice setting: Development and preliminary assessment of the Italian LCP Program. *American Journal of Hospice and Palliative Medicine,* **31**(1), pp. 61–68.

^xxvi^ WEST, E., ROMOLI, V., DI LEO, S., HIGGINSON, I. J., MICCINESI, G. & COSTANTINI, M. (2014). Feasibility of assessing quality of care at the end of life in two cluster trials using an after-death approach with multiple assessments. *BMC Palliative Care,* **13**(1), pp. 1–8.

^xxvii^ DI LEO, S., ROMOLI, V., HIGGINSON, I. J., BULLI, F., FANTINI, S., SGUAZZOTTI, E. & COSTANTINI, M. (2015). ‘Less ticking the boxes, more providing support’: A qualitative study on health professionals’ concerns towards the Liverpool Care of the Dying Pathway. *Palliative Medicine,* **29**(6), pp. 529–537.

^xxviii^ VERHOFSTEDE, R., SMETS, T., COHEN, J., COSTANTINI, M., VAN DEN NOORTGATE, N., HEIDE, A. & DELIENS, L. (2015a) Development of the care programme for the last days of life for older patients in acute geriatric hospital wards: A phase 0-1 study according to the Medical Research Council Framework. BMC Palliative Care, 14(1), pp. 1–10.

^xxix^ COSTANTINI, M., OTTONELLI, S., CANAVACCI, L., PELLEGRINI, F. & BECCARO, M. (2011). The effectiveness of the Liverpool care pathway in improving end of life care for dying cancer patients in hospital. A cluster randomised trial. *BMC Health Services Research,* **11**(1), pp. 13–13.

^xxx^ COSTANTINI, M., ROMOLI, V., DI LEO, S., BECCARO, M., BONO, L., PILASTRI, P., MICCINESI, G., VALENTI, D., PERUSELLI, C., BULLI, F. & FRANCESCHINI, C. (2014b). Liverpool Care Pathway for patients with cancer in hospital: a cluster randomised trial. *The Lancet,* **383**(9913), pp. 226–237.

^xxxi^ COSTANTINI, M. & DI LEO, S. (2014). Comment to the article: Wise J. Five priorities of care for dying people replace Liverpool care pathway. *Italian Journal of Medicine,* **8**(4), pp. 265–267.

^xxxii^ VERHOFSTEDE, R., SMETS, T., COHEN, J., COSTANTINI, M., VAN DEN NOORTGATE, N. & DELIENS, L. (2015b). Improving end-of-life care in acute geriatric hospital wards using the Care Programme for the Last Days of Life: study protocol for a phase 3 cluster randomized controlled trial. *BMC Geriatrics,* **15**(1), pp. 13–13.

^xxxiii^ VERHOFSTEDE, R., SMETS, T., COHEN, J., COSTANTINI, M., VAN DEN NOORTGATE, N. & DELIENS, L. (2016). Implementing the care programme for the last days of life in an acute geriatric hospital ward: a phase 2 mixed method study. *BMC Palliative Care,* **15**(1), pp. 1–12.

^xxxiv^ BEERNAERT, K., SMETS, T., COHEN, J., VERHOFSTEDE, R., COSTANTINI, M., EECLOO, K., VAN DEN NOORTGATE, N. & DELIENS, L. (2017). Improving comfort around dying in elderly people: a cluster randomised controlled trial. The Lancet, 390(10090), pp. 125–134.

^xxxv^ BRÄNNSTRÖM, M., FÜRST, C. J., TISHELMAN, C., PETZOLD, M., LINDQVIST, O., MEDICINSKA, F., UMEÅ, U. & INSTITUTIONEN FÖR, O. (2016). Effectiveness of the Liverpool care pathway for the dying in residential care homes: An exploratory, controlled before-and-after study. *Palliative Medicine,* **30**(1), pp 54–63.

^xxxvi^ SWART, S., VAN VELUW, H., KONINGSWOUD, J., BAAR, F., VAN DER RIJT, C. & VAN ZUYLEN, L. V. (2003). 'Liverpool integrated Care Pathway' naar 'Zorgpad voor de Stervensfase-Rotterdam'. Nederlands tijdschrift voor palliatieve zorg, 1, pp. 12–161.

^xxxviii^ SWART, S., VELUW, H., ZUYLEN, L., GAMBLES, M. & ELLERSHAW, J. (2006). Dutch experiences with the Liverpool Care Pathway. European Journal of Palliative Care, 13(4), pp. 156–159.

^xxxix^ GEIJTEMAN, E., DEKKERS, A. G. W. M. & VAN ZUYLEN, L. (2013). 10 jaar Zorgpad Stervensfase: belangrijke verbeteringen in de zorg in de laatste levensdagen. *Nederlands Tijdschrift voor Geneeskunde,* **157**(37), pp.1–4.

^xl^ RAIJMAKERS, N., DEKKERS, A., GALESLOOT, C., VAN ZUYLEN, L. & VAN DER HEIDE, A. (2015). Barriers and facilitators to implementation of the Liverpool Care Pathway in the Netherlands: a qualitative study. *BMJ Supportive & Palliative Care,* **5**(3), pp. 259–265.

^xli^ AFZAL, N., BUHAGIAR, K., FLOOD, J. AND COSGRAVE, M. (2010). Quality of end-of-life care for dementia patients during acute hospital admission: a retrospective study in Ireland. *General Hospital Psychiatry*, **32**(2), pp.141–146.

^xlii^ GÖSSI, U. (2012). Palliative Care: "we have always done it like that!" Problems and experience in palliative care from the point of view of an internist. *Therapeutische Umschau. Revue therapeutique*, **69**(2), pp. 75.

^xliv^ VOLTZ, R., NUBLING, G. & LORENZL, S. (2013). ‘Care for the dying neurologic patient’ in: Bernat, J and Beresford, R. (ed.) *Ethical and Legal Issues in Neurology*. Amsterdam, Elsevier. pp. 141–145.

^xlv^ ZINNER, M. (2013). *Der "Liverpool Care Pathway": Ein Behandlungspfad zur Begleitung Sterbender,* Munich, GRIN Verlag.

^xlvi^ MULLER, E. (2011). *Der Liverpool care Pathway for the Dying Patient” als Navigationshilfe: Die Vorstellung eines Leitfadens zur Begleitung Sterbender.* Nordensredt, Books on Demand.

^xlvii^ CHAN, R. J., WEBSTER, J., PHILLIPS, J. & CURROW, D. C. (2014). The withdrawal of the Liverpool Care Pathway in the United Kingdom: what are the implications for Australia? *Medical Journal of Australia,* **200**(10), pp. 573–573.

^xlviii^ MENESES, J., FLODGREN, G. & BERG, R. C. (2016). *Bruk av Liverpool Care Pathway ved livets slutt*. Norsk folkehelseinstitutt, Oslo. URL: https://www.fhi.no/en/publ/2016/liverpool-care-pathway-end-of-life/.

^xlix^ HUSEBØ, B. S., FLO, E. & ENGEDAL, K. (2017). The Liverpool Care Pathway: a systematic review discarded in cancer patients but good enough for dying nursing home patients? *BMC Medical Ethics,* **18**(1), pp. 1–13. An Erratum followed the original article: Erratum to: The Liverpool Care Pathway: discarded in cancer patients but good enough for dying nursing home patients? A systematic review BS Husebø, E Flo, K Engedal *BMC Medical Ethics*, 2017 - bmcmedethics.biomedcentral.com After publication of the article, it was brought to the attention of the authors that the wrong title was used on initial publication. The correct title of this article is “The Liverpool Care Pathway: discarded in cancer patients but good enough for dying nursing home patients? A systematic review”. The original version of the article has been updated to reflect this. See: Husebø, B. S., Flo, E., Engedal, K. (2017b). Erratum to: The Liverpool Care Pathway: discarded in cancer patients but good enough for dying nursing home patients? A systematic review. *BMC Med Ethics, 18*(1), 52.

^l^ HONGYAN, NIU., PEIXI, WANG. & XINMING, ZHOU. (2011). The development of hospice nursing in China and the Liverpool Care Pathway. *Chinese Nursing Journal*, **25**(2b): 384–385.

^li^ YAMAGISHI, A., MORITA, T., MIYASHITA, M., AKIZUKI, N., KIZAWA, Y., SHIRAHIGE, Y., AKIYAMA, M., HIRAI, K., KUDO, T., YAMAGUCHI, T., FUKUSHIMA, A. & EGUCHI, K. (2008). Palliative Care in Japan: Current Status and a Nationwide Challenge to Improve Palliative Care by the Cancer Control Act and the Outreach Palliative Care Trial of Integrated Regional Model (OPTIM) Study. *American Journal of Hospice and Palliative Medicine,* **25**(5), pp. 412–418.

^lii^ MIYASHITA, M. (Ed.) (2010). リバプール・ケア・パスウェイを用いた看取りのケアの質向上 [Rethink palliative care: using the Liverpool Care Pathway to improve the quality of palliative care]. [Special issue]. 臨牀看護 *Clinical Nursing*, 36.

^liii^ MIYASHITA, M. (2010). 特集に当たって [About this special issue]. [Special issue]. 臨牀看護 *Japanese Journal Clinical Nursing*. 36, pp.1812–14.

^liv^ FUKUTA, K. (2010). 事例を用いたLCPの実際 [Examples of LCP in practice]. [Special issue]. 臨牀看護 *Japanese Journal Clinical Nursing*. 36, pp.1849–61.

^lv^ OTANI, H (2010). エビデンスに基づいた看取りのケア；看取りの支援小冊子の紹介 [Evidence-based end of life care: introduction to a support booklet of end of life care]. [Special issue]. 臨牀看護Japanese Journal Clinical Nursing. 36, pp.1869–80.

^lvi^ YOSHIKAZU, C. (2016). A medical pathway of end of life care – current status and future of the Japanese version of the Liverpool Care Pathway. *The Japanese Journal of Clinical Research on Death and Dying,* **39** (1), pp. 17–18.

^lvii^ CHINONE, Y. (2016). 看取りのケアのクリニカルパス: Liverpool Care Pathway 日本語版の現在とこれから [A clinical pathway of end of life care: the present and future of the Japanese version of the Liverpool Care Pathway]. *Shinorinsho* 死の臨床. 39, pp.17–8.

^lviii^ TANAKA, M AND KODAMA, S. (2017). End of Life Care. In: M. Tanaka & S Kodama. *Choice of the ending: to think about the end of life care* ( *終の選択：終末期医療を考える*). Tokyo: Keiso Shobo.

^lix^ COSTANTINI, M. & DI LEO, S. (2014). Comment to the article: Wise J. Five priorities of care for dying people replace Liverpool care pathway. *Italian Journal of Medicine,* **8**(4), pp. 265–267.

^lx^ COSTANTINI, M., ALQUATI, S. & DI LEO, S. (2014). End-of-life care: pathways and evidence. Current Opinion in Supportive and Palliative Care, **8**(4), pp.399–404.

## References

[1] Greenhalgh T Robert G Macfarlane F: Diffusion of innovations in service organizations: systematic review and recommendations. *Milbank Q.* 2004;82(4):581–629. 10.1111/j.0887-378X.2004.00325.x 15595944 PMC2690184

[2] See: https://www.bbc.co.uk/programmes/b03859nf, accessed 3 September 2020.

[3] Knights D Wood D Barclay S: The Liverpool Care Pathway for the dying: what went wrong? *Br J Gen Pract.* 2013;63(615):509–10. 10.3399/bjgp13X673559 24152449 PMC3782767

[4] Borgstrom E: What’s in a name? From pathways to plans in end of life care. *BMJ.* 2013;347:f4957. 10.1136/bmj.f4957 23945361

[5] Wrigley A: Ethics and end of life care: the Liverpool Care Pathway and the Neuberger Review. *J Med Ethics.* 2015;41(8):639–43. 10.1136/medethics-2013-101780 24850872

[6] Prentice J Amer Y: The rise and fall of the Liverpool care pathway. *Prog Palliat Care.* 2016;24(2):98–100. 10.1080/09699260.2015.1103465

[7] Jones AR: The Liverpool Care Pathway for the dying patient: Euthanasia through the back door, or the sign of poor death education? *Ethics & Bioethics.* 2020;10(1–2):40–47. 10.2478/ebce-2020-0006

[8] Billings JA Block SD: The Demise of the Liverpool Care Pathway? A Cautionary Tale for Palliative Care. *J Palliat Med.* 2013;16(12):1492–5. 10.1089/jpm.2013.0493 24160672

[9] Currow DC Abernethy AP: Lessons from the Liverpool Care Pathway--evidence is key. *Lancet.* 2014;383(9913):192–3. 10.1016/S0140-6736(13)62039-5 24139707

[10] Stocker R Close H: Assessing the uptake of the Liverpool Care Pathway for dying patients: a systematic review. *BMJ Support Palliat Care.* 2013;3(4):399–404. 10.1136/bmjspcare-2012-000406 24950519

[11] Venkatasalu MR Whiting D Cairnduff K: Life after the Liverpool Care Pathway (LCP): a qualitative study of critical care practitioners delivering end-of-life care. *J Adv Nurs.* 2015;71(9):2108–2118. 10.1111/jan.12680 25974729

[12] See: https://cordis.europa.eu/project/id/202112/reporting, accessed 30 July 2020.

[13] Costantini M Lunder U: OPCARE9 - A European perspective on the last days of life. *European Journal of Palliative Care.* 2012;19(4):175–177. Reference Source

[14] Ellershaw J Fürst CJ Lunder U: Care of the dying and the LCP in England: An international perspective. *Eur J Palliat Care.* 2013;20(3):120–123. Reference Source

[15] Butler I Drakeford M: Scandal, Social Policy and Social Welfare.Bristol: Policy Press. 2005 Reference Source

[16] See: http://www.pcil.org.uk/media/33885/position%20statement.pdfaccessed 30 July 2020.

[17] See: https://bestcareforthedying.org/accessed 30 July 2020.

[18] The signatories to the 2013 position statement, from 12 countries including the UK and almost all of whom are represented as authors to papers discussed here or as participants in our interviews, were: Professor John Ellershaw - MA FRCP Professor of Palliative Medicine, University of Liverpool Director, Marie Curie Palliative Care Institute Liverpool (MCPCIL) Liverpool, UK Professor Carl Johan Furst Palliative Centre, Lund University Lund, Sweden Deborah Murphy Associate Director, Marie Curie Palliative Care Institute Liverpool (MCPCIL) Liverpool, UK Dr Simon G. Allan Director of Palliative Care Arohanui Hospice Palmerston North New Zealand Associate Professor Mark Boughey Director of Palliative Medicine, St Vincent’s Hospital Melbourne, Melbourne, Australia Associate Professor and Co-Deputy Director, The Centre of Palliative Care, University of Melbourne, Australia President, the Australian and New Zealand Society of Palliative Medicine International Collaborative Briefing Paper February 2014 Massimo Costantini - Physician, MD Palliative Care Unit IRCCS Arcispedale S. Maria Nuova Italy Professor Gustavo De Simone Pallium Latinoamerica Buenos Aires, Argentina Steffen Eychmueller - MD, MME Center for Palliative Care, SWAN House University Hospital, Inselspital Berne Bern, Switzerland Dagny Faksvåg Haugen Lead Consultant, Head of Centre Regional Centre of Excellence for Palliative Care, Western Norway Haukeland University Hospital Bergen, Norway Urska Lunder - MD Palliative Care Unit Lead University Clinic for Respiratory and Allergic Diseases Golnik, Slovenia Marisa Martin-Rosello CEO and Medical Director Cudeca Hospice Foundation Centro Cudeca Spain Professor Raymond Voltz Department of Palliative Medicine University Hospital of Cologne Cologne, Germany Helen Walker Program Manager – Palliative Care, Western Australia (WA) Cancer & Palliative Care Network Department of Health WA Perth, Australia Dr. Lia van Zuylen Erasmus MC Dept of Medical Oncology Rotterdam, The Netherlands

[19] Seymour J Clark D: The Liverpool Care Pathway for the Dying Patient: a critical analysis of its rise, demise and legacy in England [version 2; peer review: 2 approved]. *Wellcome Open Res.* 2018;3:15. 10.12688/wellcomeopenres.13940.2 29881785 PMC5963294

[20] See: http://liverpool-care-pathway-a-national-sc.blogspot.com/2013/04/liverpool-care-pathway-dangers-which.htmlaccessed 30 July 2020.

[21] Dolowitz DP Marsh D: Who Learns What from Whom? A Review of the Policy Transfer Literature. *Polit Stud (Oxf).* 1996;44(2):343–57. 10.1111/j.1467-9248.1996.tb00334.x

[22] Benson D Johnson A: What Have We Learned from Policy Transfer Research? Dolowitz and Marsh Revisited. *Political Studies Review.* 2011;9(3):366–378. 10.1111/j.1478-9302.2011.00240.x

[23] Zaman S Inbadas H Whitelaw A: Common or multiple futures for end of life care around the world? Ideas from the ’waiting room of history'. *Soc Sci Med.* 2017;172:72–9. 10.1016/j.socscimed.2016.11.012 27894008 PMC5224187

[24] Freeman R: What is 'Translation'? *Evidence and Policy.* 2009;5(4):429–447. 10.1332/174426409X478770

[25] Star SL Griesemer JR: Institutional ecology, 'translations' and boundary objects: amateurs and professionals in Berkeley's Museum of Vertebrate Zoology, 1907-39. *Soc Stud Sci.* 1989;19:387–420. 10.1177/030631289019003001

[26] Dolowitz DP Marsh D: Learning from abroad: the role of policy transfer in contemporary policy making. *Governance.* 2000;13(1):5–24. 10.1111/0952-1895.00121

[27] We are grateful to Dr Anne Grinyer for this point..

[28] Clark D: International transfer and translation of an end of life care intervention: the case of the Liverpool Care Pathway for the Dying Patient. [Data Collection]. 2020. 10.12688/wellcomeopenres.16321.1 PMC7726848 33344784

[29] Slovenia was the only country for which we could find no published outputs, beyond the description in the LCP handbook chapter..

[30] Greenhalgh T Peacock R: Effectiveness and efficiency of search methods in systematic reviews of complex evidence: audit of primary sources. *BMJ.* 2005;331(7524):1064–1065. 10.1136/bmj.38636.593461.68 16230312 PMC1283190

[31] Gerson K Horowitz R: Observation and interviewing: Options and choices in qualitative research.In: T. May (Ed.), Qualitative research in action. London: Sage Publications. 2002 10.4135/9781849209656.n9

[32] Gale NK Heath G Cameron E: Using the framework method for the analysis of qualitative data in multi-disciplinary health research. *BMC Med Res Methodol.* 2013;13:117. 10.1186/1471-2288-13-117 24047204 PMC3848812

[33] Smeding R Bolger M Ellershaw J: International development of the Liverpool Care Pathway for the Dying Patient (LCP). In J Ellershaw and S Wilkinson (eds). *Care of the Dying: A Pathway to Excellence.*2nd Edition, New York: Oxford University Press.2011;189–205. 10.1093/acprof:oso/9780199550838.003.0011

[34] We wrote to Professor Ellershaw on 15 May 2017 about our project on the international spread of the LCP, seeking his assistance and participation, which he declined..

[35] Clark D Graham F: The Hospice Friendly Hospitals Programme in Ireland: A Narrative History.Dublin: Irish Hospice Foundation.2013; accessed 2 January 2020. Reference Source

[36] See: https://qol.eortc.org/translations/, accessed 30 June 2020.

[37] For details of STAS, see: https://www.kcl.ac.uk/cicelysaunders/research/outcome/pos/stas, accessed 15 September 2020.

[38] Tripodoro VA Goldraij G Daud ML: Análisis de los resultados de un programa de calidad en cuidados paliativos para los últimos días de vida. Diez años de experiencia [Analysis of the results of a palliative care quality program for the last days of life. Ten years of experience]. *Medicina (B Aires).* 2019;79(6):468–476. 31829949

[39] Campbell M Fitzpatrick R Haines A: Framework for the design and evaluation of complex interventions to improve health. *BMJ.* 2000;321(7262):694–6. 10.1136/bmj.321.7262.694 10987780 PMC1118564

[40] Teno JM Clarridge B Casey V: Validation of toolkit after-death bereaved family member interview. *J Pain Symptom Manage.* 2001;22(3):752–758. 10.1016/s0885-3924(01)00331-1 11532588

[41] Hunt KJ Shlomo N Richardson A: VOICES redesign and testing to inform a national end of life care survey.Southampton: University of Southampton.2011 Reference Source

[42] Chan R: End-of-life care pathways for improving outcomes in caring for the dying. *Cochrane Database Syst Rev.* 2010; (1):CD008006. 10.1002/14651858.CD008006.pub2 20091660

[43] Craig P Dieppe P Macintyre S: Developing and evaluating complex interventions: the new medical research council guidance. *BMJ.* 2008;337:a1655. 10.1136/bmj.a1655 18824488 PMC2769032

[44] Aslakson RA Lorenz K: Being CAREFuL about improving end-of-life care in hospitals. *Lancet.* 2017;390(10090):97–98. 10.1016/S0140-6736(17)31325-9 28526494

[45] Ellershaw JE: An integrated care pathway for the dying. *Ned Tijdschr Palliat Zorg.* 2002;5(2):41–4.

[46] Swart SJ Zuylen C van Ellershaw JE: Moving to setting standards of care for the dying.

[47] Zuylen C Swart SJ Veluw H: Outcomes of care in the dying phase: comparison between the United Kingdom and the Netherlands. *8th Congress of the European Association for Palliative Care*, held in the Hague, 2–5 April,2003.

[48] Steiner G: After Babel. Aspects of language and translation. third edition, Oxford: Oxford University Press.1998 Reference Source

[49] Clark D Baur N Clelland D: Mapping levels of palliative care development in 198 countries: the situation in 2017. *J Pain Symptom Manage.* 2020;59(4):794–807.e4. 10.1016/j.jpainsymman.2019.11.009 31760142 PMC7105817

[50] Clark D Inbadas H Colburn B: Interventions at the end of life – a taxonomy for ‘overlapping consensus’ [version 1; peer review: 2 approved, 1 approved with reservations]. *Wellcome Open Res.* 2017;2:7. 10.12688/wellcomeopenres.10722.1 28261674 PMC5336190

[51] Knaul FM Bhadelia A Rodriguez NM: The Lancet Commission on Palliative Care and Pain Relief—findings, recommendations, and future directions. *The Lancet Global Health.* 2018;6:S5–S6. 10.1016/S2214-109X(18)30082-2

[52] May C: Towards a general theory of implementation. *Implement Sci.* 2013;8:18. 10.1186/1748-5908-8-18 23406398 PMC3602092

