# Supplementary material for: International transfer and translation of an end of life care intervention: the case of the Liverpool Care Pathway for the dying patient
Source: Wellcome Open Res. 2020 Oct 27;5:256. [Version 1] doi: 10.12688/wellcomeopenres.16321.1 (PMC7726848; doi:10.12688/wellcomeopenres.16321.1)
Supplement: Peer review history

# Peer review: Reviewer response for version 1

**DOI:** 10.21956/wellcomeopenres.17940.r41550

**Published:** 2020-12-09

**Authors:** Sonja McIlfatrick, Deborah Muldrew

**Affiliations:** 1 Institute of Nursing and Health Research and School of Nursing, Ulster University, Newtownabbey, UK

Thank you for the opportunity to review this important report that seeks to ascertain how and why the Liverpool Care Pathway (LCP) was transferred to 20 countries beyond the UK and with what consequences.

 This report seeks to address a significant question and gap in terms of the literature. As noted by the authors the discontinuation of the LCP in the UK had a significant impact on modern palliative care at the time and yet a gap existed in terms of international context and questions around transferability and transference of such tools and initiatives in practice. The authors suggest this should assist in offering wider lessons about policy transfer in end-of-life care. Given the increasing emphasis on global working and World Health Assembly Resolution in 2014 and recent developments and lessons from COVID-19 pandemic, this would seem to be a timely report. Furthermore, it is important that as a community we do engage in critical reflection about the risks and benefits of transferring such interventions across international boundaries. 

**Overall**, the report offers a critical and thorough review of the transference of the LCP internationally, and is well supported by appropriate and current literature. The aim was to construct a detailed case study of how the LCP was adopted internationally. To do this the authors drew on a theoretical framework derived from policy transfer literature. This posed 6 key questions: 1) who the key actors were involved; 2) what was transferred; 3) from where lessons were drawn; 4) the different degrees of transfer that took place; 5) what restricted or facilitated transfer; and 6) how transfer was related to ‘success’ or ‘failure’. The authors contend that the use of such an approach helps to make sense about the international reach of LCP and assists in deeper understanding and helps to inform the research questions.

 Using this, **the methods** include two aspects: 1) a historical narrative review drawing on published and grey literature relating to LCP in international contexts (n=95) and 2) qualitative interviews (n=18) with key actors in involved in LCP implementation, research or discussion in countries outside the UK. The design and methods flow logically from the research question and are methodologically sound. Sufficient detail is provided, inclusive of practical methodological detail, to allow for replication and assessment of rigour. For example, in Phase 1: narrative review sufficient detail is provided in terms of inclusion and exclusion criteria, clearly outlined that the focus was published articles, conference abstracts and presentations and wider commentaries that focus on the use of the LCP in countries outside of the UK. Whilst phase 2 qualitative interviews employed both purposive and snowball sampling involving a breadth of experts from clinicians with experience of LCP implementation, researchers outside of the UK, policy makers, and global experts in palliative care. A strength of the study was the use of initial sampling frame derived from LCP framework, building to include those who were identified from the narrative review of the literature and from other informants. Conversely, however, the authors acknowledge the limitation with purposive sampling including those who could be viewed as LCP enthusiasts, which may not reflect the wider views of both clinical and research population. Other limitations include only having 14 countries included in the interview process, whereas the literature would indicate interest in 6 other areas. 

**Phase 1 Narrative Review: **The reporting of results for the narrative review is both comprehensive and well structured. The use of a clear structure categorising studies as audit, implementation analysis; controlled studies and commentaries enabled the reader to clearly follow the evidence base and note the conclusions from an international cross-country perspective over time. The overall conclusions from the narrative review are clearly outlined and well justified from the extensive review and evidence base. One limitation of the narrative review to be acknowledged is the challenge of comparing many studies with varying deviances from the original LCP model. Whilst some papers followed the LCP in detail, others only used LCP guiding principles and developed additional documentation, such as handbooks for example, to support the implementation in practice. Therefore, this raises questions around the evidence and whether the successes of each study be directly linked back to the original LCP model or at least in part to the iterations subsequently made.

**Phase 2 Interviews:** Clinical specialists and researchers in palliative care across multiple organisations were identified as the key actors involved in promoting and transferring the LCP. The authors clearly noted those key informants that either declined or were unable to be contacted, again enhancing the transparency and rigour of the approach. The interview questions were informed by the 6 areas related to policy transfer noted previously. It was noteworthy that the nature of “what” was transferred ranged from a precise and exact translation of the LCP to a guiding set of principles underpinning end of life care. In terms of clinical representation from a multidisciplinary perspective it was noted that all but one of the participants was from a medical background, yet statements highlight the importance of ACP for nursing staff and yet their participation in the interview phase was lacking. This could be included within the acknowledged limitations of including those who were identified as ‘LCP enthusiasts’. Questions also remain around why further views from policy makers were not included within this phase of the report.

**The discussion and conclusion** of the report draws on the key points identified in the two phases and data sets, again using policy transfer as a structure. The use of the two complementary and yet distinct data sets help to clearly reinforce and provide evidence for the final conclusions. The key elements of failure were clearly noted and evidenced from the 2 data sets: (a) the lack of policy recognition and investment it needed to become established and integrated at the level of the care system and (b) a lack of cumulative evidence base that fully recognised its complexity as an intervention.

 Lessons drawn from the report in the conclusion section point to the need for caution with interpretation of results, due to a tendency for modest results to be interpreted in a positive light. Despite evidence from studies using rigorous designs that the effect of the LCP in comparison to normal care was mostly not statistically significant, smaller studies reported inflated benefits. Stakeholder support, educational and workforce limitations, and resource availability impacted on transference. Whilst new partnerships and collaborations in clinical practice, clinician awareness of the need for a structured approach to end of life care, and acceptability of the model by caregivers were seen as successes, failures included limited policy recognition and investment, and insufficient gathering of an evidence base to support the LCP. The results and conclusions presented reflect the aims and research questions driving the research and can be clearly supported by the data available.

 The overall report concludes by providing a useful synthesis of the learning gained from the 2 data sets by identifying 5 dimensions that shared the transfer of the LCP beyond the UK. This provides a useful synthesis of the results and a key aspect for future considerations. 

1. We need to understand policy transfer in a period of *historical time*, in this case when the discipline of palliative care was seeking to gain traction and promote its knowledge and methods beyond the specialist services in which it had originated.
2. The transfer is in turn shaped by particular *actions and processes* that in our worked example sought to promote, implement and validate the LCP as a transformative care pathway for the end of life.
3. To do this LCP had to be lodged within *systems* in which it could operate effectively and deliver measurable change.
4. How this was achieved depended significantly on the *cultures, geographies and settings* in which it was placed, and these could take many forms.
5. Success in all this required clarity about *goals, outcomes and related consequences*.

This is an interesting and highly relevant report that adds to the knowledge base and provides a useful platform for future discussions for the discipline of palliative care.

Is the work clearly and accurately presented and does it cite the current literature?

Yes

If applicable, is the statistical analysis and its interpretation appropriate?

Not applicable

Are all the source data underlying the results available to ensure full reproducibility?

Yes

Is the study design appropriate and is the work technically sound?

Yes

Are the conclusions drawn adequately supported by the results?

Yes

Are sufficient details of methods and analysis provided to allow replication by others?

Yes

Reviewer Expertise:

Palliative care, end of life care, review methodology, mixed methods research, intervention research, clinical nursing

We confirm that we have read this submission and believe that we have an appropriate level of expertise to confirm that it is of an acceptable scientific standard.

# Peer review: Reviewer response for version 1

**DOI:** 10.21956/wellcomeopenres.17940.r41168

**Published:** 2020-11-20

**Authors:** Lisbeth Thoresen

**Affiliations:** 1 Department of Interdisciplinary Health Sciences, Institute of Health and Society, University of Oslo, Oslo, Norway

**Reviewer Report:**

 This is indeed a very interesting and important paper. Congratulations to the authors. For all of us who have followed the rise and fall of the UK-based Liverpool Care Pathway (LCP) from a distance, this paper brings new insights into why and how the pathway could actually spread internationally as it did, and how it impacted end-of-life care practices in different countries. Together with a critical paper on the LCP by Jane Seymour and David Clark in 2018 (also published by Wellcome Open Research), the present paper may work as a key reference not only to the story of the LCP but also to knowledge transfer and translation in general.

 The paper is based on a historical narrative literature review on the international implementation of the LCP, together with data from 18 qualitative interviews with 19 people. Those interviewed are described as key actors involved in transferring and translating the LCP into 14 countries. The concept of ‘policy transfer’ is part of the theoretical framework, and implies that knowledge translation is also about transferring ideas and practices internationally, from one country to another. Another theoretical concept is ‘the boundary object’, which was also used earlier in the aforementioned 2018 paper.

 When it comes to the number of published articles included in the literature review, I was particularly interested in contributions from my home country, Norway. The literature search (2000–19) resulted in only three Norwegian publications. I suggest adding an article by (Brattgjerd and Olsen, 2016) ^1^ on the use and experience of the LCP in Norway. This publication is authored by nurses and describes nurses’ experiences of the LCP in nursing homes. Descriptions on how the LCP was implemented and experienced in one municipality ( https://www.utviklingssenter.no/prosjekter/lindrende-behandling/implementering-av-liverpool-care-pathway-i-vestfold), and the closing report from the same municipality ( https://www.sandefjord.kommune.no/globalassets/helse-sosial-og-omsorg/hso-dokumenter/utviklingssenter/administrative-dokumenter/lcp-sluttrapport-sept.-2013-2.pdf), may also shed light on the transfer and translation of the LCP to the Norwegian context. Under the heading ‘Commentaries and other publications’, the authors of an article (Husebø et al., 2017) ^2^ are referred to, stating that no open and critical debate has taken place about the LCP in Norway (or in other Scandinavian countries). This may be the case among researchers and healthcare professionals; however, in my experience, critique has been raised, but mostly in local newspapers.

 The point I want to make for the case of Norway, and which I guess also applies to other countries, is that it is possible to find further outputs, which may inform the conclusions of the historical narrative review. To include newspaper or social media debates on the LCP may be worth considering, perhaps in another paper.

 When it comes to the interviews, I want to make the same point. By including only clinicians, policymakers and global experts in the paper referred to as LCP enthusiasts and the LCP elite, the views and experiences from particular nurses and other healthcare professionals working close to dying patients are missing. The authors describe this as a possible limitation, and my claim is that this is a weakness in the study. Particularly from a Norwegian perspective where, in a short time, the LCP spread to many of the municipalities, nurses played a very important role in transferring and translating the LCP to local, often small, contexts. They were the ones who were practising the LCP on a daily basis. When critique was raised about the LCP in Norway, nurses may have had a hard time explaining and defending the pathway.

 Despite my critical comments, there is no doubt that the paper contributes to a richer understanding of the complexity involved in transferring and translating ideas and practices from one country to another. I hope for further analysis on the same issue.

**Questions:**

- Is the work clearly and accurately presented and does it cite the current literature? **Yes**
- Is the study design appropriate and does the work have academic merit? **Yes**
- Are sufficient details of methods and analysis provided to allow replication by others? **Yes**
- If applicable, is the statistical analysis and its interpretation appropriate? **Not applicable**
- Are all the source data underlying the results available to ensure full reproducibility **? For the literature review: Yes. For the interviews:  the full analysis of the interviews is included as underlying data. **
- Are the conclusions drawn adequately supported by the results? **Yes**

**Approval status:** Approved

Is the work clearly and accurately presented and does it cite the current literature?

Yes

If applicable, is the statistical analysis and its interpretation appropriate?

Not applicable

Are all the source data underlying the results available to ensure full reproducibility?

Partly

Is the study design appropriate and is the work technically sound?

Yes

Are the conclusions drawn adequately supported by the results?

Yes

Are sufficient details of methods and analysis provided to allow replication by others?

Yes

Reviewer Expertise:

My area of research is interdisciplinary studies and perspectives on dying and death

I confirm that I have read this submission and believe that I have an appropriate level of expertise to confirm that it is of an acceptable scientific standard.

## References

[1] : Omsorg ved livets slutt - En kvalitativ studie av sykepleieres erfaringer med bruk av Liverpool Care Pathway i sykehjem. *Tidsskrift for omsorgsforskning*.2016;2(03) : 10.18261/issn.2387-5984-2016-03-05 189-201 10.18261/issn.2387-5984-2016-03-05

[2] : The Liverpool Care Pathway: discarded in cancer patients but good enough for dying nursing home patients? A systematic review. *BMC Medical Ethics*.2017;18(1) : 10.1186/s12910-017-0205-x 10.1186/s12910-017-0205-x 28793905PMC5551006
